# Food for everyone: Differential feeding habits of cryptic bat species inferred from DNA metabarcoding

**DOI:** 10.1111/mec.16073

**Published:** 2021-07-23

**Authors:** Tommy Andriollo, Johan R. Michaux, Manuel Ruedi

**Affiliations:** ^1^ Department of Mammalogy and Ornithology Natural History Museum of Geneva Geneva Switzerland; ^2^ Section of Biology Faculty of Sciences University of Geneva Geneva Switzerland; ^3^ Laboratoire de Génétique de la Conservation Université de Liège, Institut de Botanique B22 Liège Belgium; ^4^ CIRAD, Agirs Unit TA C‐ 22/E‐ Campus international de Baillarguet Montpellier Cedex 5 France

**Keywords:** Bats, cryptic species, diet analysis, metabarcoding, niche partitioning, trophic ecology

## Abstract

Ecological theory postulates that niches of co‐occurring species must differ along some ecological dimensions in order to allow their stable coexistence. Yet, many biological systems challenge this competitive exclusion principle. Insectivorous bats from the Northern Hemisphere typically form local assemblages of multiple species sharing highly similar functional traits and pertaining to identical feeding guilds. Although their trophic niche can be accessed with unprecedented details using genetic identification of prey, the underlying mechanisms of resource partitioning remain vastly unexplored. Here, we studied the differential diet of three closely‐related bat species of the genus *Plecotus* in sympatry and throughout their entire breeding season using DNA metabarcoding. Even at such a small geographic scale, we identified strong seasonal and spatial variation of their diet composition at both intra‐ and interspecific levels. Indeed, while the different bats fed on a distinct array of prey during spring, they showed higher trophic niche overlap during summer and fall, when all three species switched their hunting behaviour to feed on few temporarily abundant moths. By recovering 19 ecological traits for over 600 prey species, we further inferred that each bat species used different feeding grounds and hunting techniques, suggesting that niche partitioning was primarily habitat‐driven. The two most‐closely related bat species exhibited very distinct foraging habitat preferences, while the third, more distantly‐related species was more generalist. These results highlight the need of temporally comprehensive samples to fully understand species coexistence, and that valuable information can be derived from the taxonomic identity of prey obtained by metabarcoding approaches.

## 1 INTRODUCTION

The competitive exclusion principle, or Gause's law, is a central principle in ecology positing that limited resources prevent the stable coexistence of two species relying on their similar use (Hardin, 1960). As a corollary, species living in sympatry are likely to have a differentiate use of limiting available resources –known as resource partitioning– that reduces niche overlap and stabilizes competition. Resource partitioning in co‐occurring species can be achieved along several dimensions of their ecological niche (Hutchinson, 1957). The most important ones are linked to habitat and food use (Schoener, 1974), but many additional processes have been described to complete this Hutchinsonian niche concept (Holt, 2009). Studying ecological niche dimensions can be challenging, especially when working with rare, elusive and morphologically cryptic species or when many closely related forms co‐occur in the same place. Echolocating bats represent such a group, as they can form assemblages of numerous species pertaining to the same feeding guilds, and sharing remarkably similar functional traits such as morphological and echolocation characteristics (Aldridge & Rautenbach, 1987; Mancina et al., 2012; Roswag et al., 2018; Schnitzler & Kalko, 2001; Vesterinen et al., 2018). A growing number of studies also revealed that bat communities are formed by species more closely‐related than expected by chance, a mechanism called phylogenetic underdispersion (Patrick & Stevens, 2014; Presley et al., 2018; Riedinger et al., 2013). Modern approaches to characterize indirectly the potential for interspecific competition within such species assemblages now rely on diet analyses (Salinas‐Ramos et al., 2020). Such studies benefitted from the advent of metabarcoding techniques, which provide unprecedented resolution in taxonomic identifications (Alberdi et al., 2019; Clare, 2014). Despite these technical advances, mechanisms mediating species coexistence in insectivorous bats still remain unclear, as the fitness costs implied by competing members in a community is not amenable to consumer–resource experiments (Letten et al., 2017), nor is it realistic to estimate available food offer under natural conditions for such large‐ranging aerial predators.

Two main measurements of the trophic niche are classically used to characterize feeding ecology of interacting species: the trophic niche breadth, which allows to distinguish generalist from specialist feeding strategies (Lopes et al., 2015; Salinas‐Ramos et al., 2015), and the dietary niche overlap, which measures the extent of shared food resources between species (Aldasoro et al., 2019; Chang et al., 2019; Kartzinel et al., 2015; Leray et al., 2015; Sato et al., 2018). However, such ecological indices consider each prey species as a simple resource type, and do not account for information derived from the ecology of the prey itself (Spitz et al., 2014). Some authors relied on informal considerations about major prey groups or singular prey species hunted by co‐occurring bats (Chang et al., 2019; Salinas‐Ramos et al., 2015; Vesterinen et al., 2018), while others used comparative tests to assess habitat preferences of pairs of predators based on prey species assigned to different ecological categories (Razgour et al., 2011; Roswag, Becker, Drangusch, et al., 2018; Vallejo et al., 2019). More sophisticated approaches aiming to relate characteristics of multiple consumers to habitat or functional traits of their prey with RLQ approaches (Quéméré et al., 2013; Spitz et al., 2014) offer a promising analytical development. In bats, this approach is still seldom employed but was used to compare the diet of two sympatric *Rhinolophus* species in early summer (Arrizabalaga‐Escudero et al., 2018) or at the intraspecific level in *R. euryale* (Arrizabalaga‐Escudero et al., 2019).

Here, we explored a biological system involving three long‐eared bat species that are widely distributed in Western Europe, the brown (*Plecotus auritus*), the grey (*P. austriacus*), and the alpine long‐eared bat (*P. macrobullaris*). *P*. *austriacus* probably diverged from the other two during the Middle Miocene, some 14 million years ago (Ma) (Juste et al., 2004; Spitzenberger et al., 2006). This divergence probably occurred in allopatry, when global climatic cooling events isolated ancestral populations of these poor dispersers into distinct forest refuges in Eurasia (Spitzenberger et al., 2006). The third species, *P*. *macrobullaris*, is more typical of open habitats (Alberdi & Aizpurua, 2018) and is closely related to *P. auritus* from which it diverged during the Pliocene, some 2–3 Ma (Spitzenberger et al., 2006). Two or more of these species can coexist in close vicinity in several areas but do not hybridize (Andriollo et al., 2018). Morphologically, all three species are so similar that they may be challenging to identify in the field (Andriollo & Ruedi, 2018; Ashrafi et al., 2010). Indeed, for a long time they were considered to be a single species until Bauer (1960) showed that *P*. *austriacus* differed biometrically from *P*. *auritus*, and much later when molecular analyses supported the independent species status of a third sympatric taxon, *P*. *macrobullaris* (Kiefer et al., 2002; Spitzenberger et al., 2002). The three species also have nearly identical echolocation call characteristics (Barataud, 2015; Dietrich et al., 2006) and *P*. *auritus* and *P. austriacus* have very similar wing shape (Entwistle et al., 1996) suggesting that all three have similar foraging behaviour as well (Schnitzler & Kalko, 2001). These long‐eared bats are known to feed extensively on tympanate moths (Alberdi et al., 2012; Vaughan, 1997). The diet of *P. auritus* can be more diverse as it includes also many prey from other insect orders (Ashrafi et al., 2011; Motte, 2011; Razgour et al., 2011), but microhistological identification of prey remains indicated that the diet of *P*. *austriacus* and *P*. *macrobullaris* is very similar at order level, suggesting that the latter taxa exploit the same trophic resources (Ashrafi et al., 2011). As these two species exhibit essentially parapatric distributions at the regional scale (Mattei‐Roesli, 2010; Rutishauser et al., 2012), several authors suggested that they occupy the same ecological niche, preventing their stable coexistence in sympatry (Alberdi & Aizpurua, 2018; Ashrafi et al., 2011; Dietrich et al., 2006; Rutishauser et al., 2012). However, this observation is contradicted by the co‐occurrence of both species across wide areas in the Dinaric Alps (Tvrtković et al., 2005), in Corsica (Courtois et al., 2011), in the Pyrenees (Alberdi et al., 2014), in the French Prealps (Arthur & Lemaire, 2015), or in the Geneva region (Gilliéron et al., 2015; Rutishauser et al., 2012). This hypothesis of competitive exclusion is also challenged by the fact that the distribution of *P*. *macrobullaris* is still confined to the higher elevations in the eastern part of its range, where *P*. *austriacus* does not occur (Alberdi et al., 2014).

To explore the extent of niche overlap among these highly similar long‐eared bats, we studied their diet in a unique area of sympatry where multiple colonies of all three species are established in close proximity and potentially exploit overlapping feeding grounds (Gilliéron et al., 2015). Following the optimal foraging theory, we hypothesized that the trophic niche of bats would be wider when availability in preferred resources is low (for moths typically in spring), while a narrower selection for few abundant prey groups would take place during other periods of the year. Since insect abundance and diversity are known to vary seasonally, we designed this study to cover the entire period of activity of the bats in maternity roosts (April–October). Our first goal was to document the spatial and seasonal variation of the diet of all three species simultaneously and with a high‐resolution metabarcoding technique to identify variation in trophic niche overlap. The second goal was to infer indirectly the feeding strategies of these predators using the ecological traits of all prey species identified in their diet. Our final aim was to get better insights into possible factors facilitating the local co‐occurrence of these three morphologically highly similar bat species.

## 2 MATERIALS AND METHODS

### 2.1 Field sampling

**FIGURE 1:**
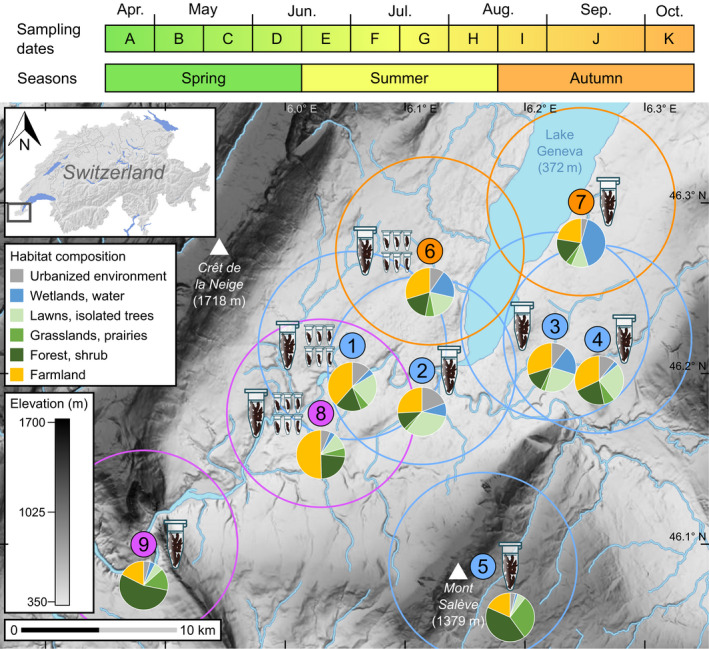
Sampling dates (upper panel) and geographic location of the nine colonies of long‐eared bats studied in the Geneva region. Elevation on the map is shaded from pale (low) to dark grey (high). Blue circles represent colonies of *P*.* auritus*: (1) Satigny, (2) pont Butin, (3) Choulex, (4) Presinge and (5) Sappey; orange ones colonies of *P*.* austriacus*: (6) Collex and (7) Hermance; purple ones colonies of *P*.* macrobullaris*: (8) Cartigny and (9) Léaz. The sampling regime aimed to gather either “community samples” (symbolized by large plastic tubes) or “smaller samples” (small plastic tubes). For each colony. the habitat composition (in a circular buffer of 6 km radius) is displayed in a pie chart. The inset (top left) provides a general view of the study area near the Lake Geneva in southwestern Switzerland

We collected guano samples from nine maternity colonies of long‐eared bats established within a radius of 18 km in the Geneva region (Figure 1). Given that these bats forage up to 6–9 km away from their roosts (Ashrafi et al., 2013; Gilliéron et al., 2015; Preatoni et al., 2011), we considered that members from these roosts could potentially access to all available habitats from this region. Long‐term monitoring (Gilliéron et al., 2015) and molecular identifications (see Andriollo & Ruedi, 2018) carried out throughout the sampling period ensured that these monospecific roosts were occupied exclusively by either brown long‐eared bats *P*. *auritus* (five colonies), grey long‐eared bats *P*. *austriacus* (two colonies) or alpine long‐eared bats *P*. *macrobullaris* (two colonies). The exact count of bats living in the colony of Léaz (number 9 in Figure 1) was unknown, but the other roosts harboured 10 to 50 adults each (Gilliéron et al., 2015). Fresh guano produced in these monospecific roosts was collected from paper sheets placed under each known cluster of bats. Paper sheets were sampled and carefully cleaned every other week, from mid‐April to mid‐October 2015. This timespan covered notably the three major seasons of activity typical of temperate bats, that is, spring (from mid‐April to mid‐June, before pups are born), summer (from mid‐June to mid‐August, when pups are reared) and autumn (from mid‐August to mid‐October, when juveniles are weaned and adults disperse to hibernacula; Figure 1). Sets of faeces were dried and stored in paper envelopes placed at –20°C until DNA extraction.

### 2.2 Extraction, sequencing and prey identification

For each of 11 dates and nine locations, we extracted “community samples” consisting in an aggregate of 15–20 pellets (about 60 mg dry weight) taken randomly from the bulk of guano collected. Such community samples therefore represent a random collection of pellets produced during two weeks by several members from a colony. Community samples are more likely to represent the overall prey spectrum consumed by animals from a given colony, rather than reflecting the hunting preference of a particular individual. This totally unobtrusive design of guano collection allowed repetitive sampling without disturbing maternity colonies and without the need to capture the animals. In addition to the community samples, we also extracted for each date six independent replicates of “smaller samples” consisting in 1–3 pellets each (about 8 mg of faecal material) taken from the same bulk of guano. These series of smaller samples were issued from three neighbouring (i.e., located within a radius of 6.5 km) colonies occupied by a different species of long‐eared bat (numbered 1, 6 and 8 in Figure 1). They were used to calculate percentage of prey occurrence across several samples (wPOO), a semi‐quantitative metrics for estimating prey abundance in faeces (Deagle et al., 2018).

DNA was extracted using QIAamp DNA Stool Mini Kit (Qiagen), following the protocol from Zeale et al. (2011) with modifications to improve yield as described in Andriollo et al. (2019). Negative PCR controls were used to ensure that no major cross‐contamination occurred during laboratory procedures (Taberlet et al., 2018). Additionally, DNA extracts were randomly distributed into sequencing plates to avoid systematical bias due to possible spillover among adjacent wells. A 157 bp‐long fragment of the COI barcode gene was amplified using ZBJ primers (ZBJ‐ArtF1c and ZBJ‐ArtR2c) and a PCR setup detailed in Zeale et al. (2011). The COI barcode is indeed currently the most efficient marker to get species‐level identifications of arthropods, as it covers the most extensive reference sequence database (Tournayre et al., 2020). Although single pairs of primers may exhibit amplification biases for particular prey species (Clarke et al., 2014; Elbrecht et al., 2016, 2019), the ZBJ primers were specifically designed to amplify a large range of arthropods from the diet of European insectivorous bats (Zeale et al., 2011). Also, they are particularly well suited to recover lepidopterans and dipterans that represent the most consumed prey of long‐eared bats (Tournayre et al., 2020).

After library construction and equimolar multiplexing of purified PCR products, the final pool was sequenced on an Illumina Genome Analyser II. Raw sequences were sorted and filtered using a script proposed by André et al. (2017) which combines functions from fastx Toolkit (http://hannonlab.cshl.edu/fastxtoolkit; 23‐09‐16) and usearch (Edgar, 2010). The paired‐end reads were assembled based on overlapping ends (at least 10 bases long and a maximum mismatch of 1), while stretches corresponding to PCR primers were removed. To discard low quality sequences and probably sequencing errors, reads containing <90% high‐quality bases (i.e., with smaller Q30 index) were filtered out, as well as those shorter than 149 bp or represented by less than five reads. Within each sample, sequences represented by less than 0.1‰ of read counts were discarded in order to ensure evenness of sequencing depth across samples and avoiding overrepresentation of very occasional prey. This threshold was chosen since recovery biases of this order of magnitude have been measured in metabarcoding analyses of mock communities (Jusino et al., 2018). Using the software megan (Huson et al., 2007), reads were then clustered into unique molecular operational taxonomic units (MOTUs) allowing for one mutation within each MOTU (Min Percent Identity: 99.0). MOTUs were then submitted to the NCBI BLAST tool (Johnson et al., 2008) for initial taxonomic identification. At first, only MOTUs with at least 98% coverage and 99% identity scores in BLAST were retained for species‐level identifications. The same MOTUs were also identified through the BOLD sequence identification engine (Ratnasingham & Hebert, 2007). This taxonomically well‐curated database allowed gaining taxonomic resolution for some MOTUs, while few others were retained to the genus or family level due to lower identity scores (>96% and >90%, respectively) or to multiple taxonomic hits. A final taxonomic check was performed manually in order to ensure that each identified MOTU indeed corresponded to a species known from local inventories of invertebrates (Andriollo et al., 2019; Merz, 2012) or at least from faunal lists from Switzerland (de Jong et al., 2014). DNA sequences and retained taxonomic assignation are available for all prey species in a public repository (https://doi.org/10.5281/zenodo.5076026.

### 2.3 Measurement of trophic niche

Prey species were treated as percentage of occurrence (wPOO; Deagle et al., 2018), meaning they were weighted for each faecal extract by the number of prey species identified in this extract. This method for describing the diet was preferred as it performs better than other indices when dealing with extremely diversified diets such as that of long‐eared bats (Andriollo, Landry, et al., 2019). To estimate the completeness of the prey spectrum evaluated for each species and season, we used the Chao2 minimum estimator of asymptotic species richness (Chao, 1987; Colwell et al., 2012) computed with the software estimates 9.1.0 (Colwell, 2013). Basic statistical tests were available in the r package stats, and PERMANOVA were performed using the package adonis (Oksanen et al., 2020).

Niche breadth of each predator was measured using the Levins’ index (Levins, 1968), whereas niche overlap between pairs of species and between colonies within species was calculated using the Morisita‐Horn's index (Horn, 1966; Morisita, 1959). Statistical significance of niche overlap was tested using the RA 3 randomization algorithm proposed by Lawlor (1980) and implemented in the package EcoSimR (Gotelli et al., 2013). To visualize niche overlaps among the three bat species, matrices of pairwise niche overlap were projected through multidimensional scaling (MDS) using the principal coordinates analysis (PCoA) function implemented in the r package ade4 (Dray & Dufour, 2007). We also carried out a simpler principal component analysis (PCA) based on the presence absence matrix of all prey species in an attempt to identify the ones best explaining similarity among samples (i.e., directly linked to niche overlap).

### 2.4 Ecological traits of the prey and predators

In order to infer indirectly the hunting habitats of the three species of long‐eared bats from their diet, we first gathered the main ecological preferences of identified prey taxa (Appendix S1) from the entomological literature (Appendix S2). This information was sorted into 17 binary ecological traits related to habitat (rows in Table 1), one categorical variable reflecting the size of the prey species (from 1 to 3), and one binary variable indicating whether the prey species was flying at night or not. This latter category characterizes prey such as syrphid flies or woodlice that must be gleaned from solid surfaces when hunted by night, as they are either strictly diurnal or flightless. For each bat colony, we characterized the habitat found in a circular buffer of 6 km radius into six variables (Appendix S3) using standardized cartographical data taken from the state of Geneva database (https://ge.ch/sitg/.

**TABLE 1:** Fourth‐corner test between taxonomic identity and ecological traits of predator colonies and ecological traits of consumed prey

Ecological traits of prey	Taxonomic and ecological traits of predators								
	*P. auritus*	*P. austriacus*	*P. macrobullaris*	Urbanized environment	Wetlands, water	Lawns, isolated trees	Grasslands, prairies	Forest, shrub	Farmland
Closed habitats	0.182 (0.001)	−0.095 (0.022)	−0.130 (0.001)	0.048 (0.247)	−0.091 (0.019)	0.033 (0.441)	0.050 (0.220)	0.011 (0.805)	−0.030 (0.442)
Woodlands, forests	0.158 (0.001)	−0.079 (0.038)	−0.115 (0.001)	0.042 (0.247)	−0.065 (0.079)	0.031 (0.414)	0.034 (0.334)	0.006 (0.864)	−0.045 (0.222)
Exclusively diurnal or flightless	0.112 (0.001)	−0.061 (0.084)	−0.077 (0.023)	0.048 (0.175)	−0.045 (0.199)	0.034 (0.378)	0.014 (0.665)	−0.001 (0.992)	−0.042 (0.251)
Hedgerows	0.080 (0.012)	−0.036 (0.167)	−0.063 (0.029)	0.022 (0.369)	−0.046 (0.093)	0.014 (0.602)	0.027 (0.305)	0.015 (0.566)	−0.034 (0.177)
Shrublands	0.051 (0.129)	−0.046 (0.105)	−0.018 (0.536)	0.012 (0.680)	−0.063 (0.024)	0.001 (0.991)	0.037 (0.148)	0.033 (0.197)	−0.005 (0.857)
Urban areas	0.029 (0.398)	0.011 (0.644)	−0.045 (0.133)	0.043 (0.073)	0.033 (0.164)	0.058 (0.017)	−0.050 (0.038)	−0.060 (0.015)	−0.012 (0.598)
Semi‐open habitats	0.017 (0.522)	−0.050 (0.054)	0.027 (0.398)	0.018 (0.459)	−0.018 (0.487)	0.003 (0.965)	−0.002 (0.966)	0.014 (0.590)	−0.002 (0.939)
Lowlands	−0.018 (0.550)	0.027 (0.259)	−0.004 (0.932)	0.002 (0.921)	0.022 (0.363)	0.022 (0.391)	−0.024 (0.339)	−0.024 (0.342)	0.010 (0.648)
Cultivated lands	−0.031 (0.284)	0.018 (0.412)	0.021 (0.463)	0.000 (0.991)	0.016 (0.518)	0.008 (0.769)	−0.018 (0.519)	−0.015 (0.563)	0.019 (0.388)
Ubiquitous	−0.041 (0.189)	0.079 (0.001)	−0.025 (0.453)	−0.004 (0.853)	0.087 (0.001)	0.023 (0.391)	−0.057 (0.033)	−0.062 (0.017)	−0.009 (0.687)
Largeness (size)	−0.021 (0.524)	0.066 (0.013)	−0.037 (0.202)	−0.019 (0.461)	0.051 (0.044)	−0.002 (0.964)	−0.011 (0.678)	−0.019 (0.453)	−0.055 (0.024)
Mesophilous areas	−0.069 (0.025)	0.027 (0.274)	0.058 (0.066)	−0.048 (0.068)	0.013 (0.600)	−0.048 (0.079)	0.017 (0.565)	0.030 (0.297)	0.016 (0.474)
Dry areas	−0.035 (0.267)	−0.042 (0.147)	0.082 (0.006)	−0.013 (0.641)	−0.039 (0.197)	−0.025 (0.368)	0.021 (0.473)	0.046 (0.110)	0.033 (0.237)
Screes	−0.059 (0.027)	−0.021 (0.714)	0.091 (0.005)	−0.041 (0.152)	−0.025 (0.430)	−0.060 (0.024)	0.042 (0.147)	0.070 (0.012)	−0.012 (0.636)
Mountainous areas	−0.086 (0.002)	−0.039 (0.229)	0.141 (0.001)	−0.068 (0.030)	−0.042 (0.190)	−0.095 (0.001)	0.068 (0.035)	0.107 (0.003)	−0.003 (0.926)
Slopes	−0.083 (0.004)	−0.047 (0.072)	0.145 (0.001)	−0.033 (0.214)	−0.067 (0.015)	−0.065 (0.014)	0.052 (0.053)	0.083 (0.004)	0.060 (0.015)
Lawns	−0.081 (0.005)	−0.029 (0.311)	0.125 (0.001)	−0.055 (0.027)	−0.027 (0.325)	−0.072 (0.006)	0.040 (0.136)	0.068 (0.009)	0.051 (0.042)
Meadows	−0.111 (0.001)	−0.030 (0.265)	0.163 (0.001)	−0.074 (0.010)	−0.044 (0.115)	−0.095 (0.001)	0.058 (0.027)	0.093 (0.003)	0.073 (0.003)
Open habitats	−0.165 (0.001)	0.034 (0.279)	0.167 (0.001)	−0.078 (0.016)	0.012 (0.689)	−0.080 (0.013)	0.021 (0.538)	0.060 (0.063)	0.054 (0.076)

Notes

For each association, correlation values are given, with adjusted *p*‐values indicated in brackets. Cells corresponding to significant positive or negative associations are coloured in green or red, respectively. Dark or light colours indicate significance levels (*p* < .01 and *p* < .05. respectively); cells corresponding to not significant associations are in white.

In order to relate the bat species, the consumed prey and their ecological traits, we used an RLQ analysis (Dolédec et al., 1996; Legendre et al., 1997) as implemented in the R package ade4. This ordination method summarizes the joint structure of the following three tables: R, containing the bat species and the habitat composition of the colony from which a sample was taken from, Q, containing the 19 ecological traits of the prey species described above, and L, the linking table containing the prey spectrum of each sample for a given date and colony. Since habitat variables characterizing the surroundings of bat colonies in the table R constituted compositional data (they sum to 100%), they were transformed using additive log‐ratio transform (Aitchison, 1982). The fourth‐corner statistics was used to test the significance of relationships between both predator and prey traits (Dray et al., 2014). Both permutation of entire rows (model 2) and columns (model 4) of the linking table were carried out, and outputs of these models were combined (model 6) in order to avoid inflated type I errors (ter Braak et al., 2012; Dray & Legendre, 2008). Significance of these fourth‐corner statistics was assessed by performing 9,999 permutations (α = 0.05), and *p*‐values were adjusted by the false discovery rate method (Benjamini & Hochberg, 1995; Dray et al., 2014).

## 3 RESULTS

### 3.1 Curated data set of consumed prey

The sequenced library of both community and smaller guano samples (284 samples in total) produced 5’016’988 Illumina reads (raw data available at https://doi.org/10.5281/zenodo.5076026, representing 1,349 distinct sequences that were clustered into 883 MOTUs. Of these MOTUs, 125 (14%) did not match to any existing reference DNA sequence and were removed from the data set. A further 46 MOTUs were excluded as they represented obvious environmental contaminants (fungi, algae, bacteria and rotifers), or were arthropod species (21 of them) known to feed on guano but not likely to be preyed upon by bats (e.g., mites and dermestid beetles). Two species of slugs, *Deroceras reticulatum* and *Arion vulgaris*, present in three distinct samples from roosts of *P*. *auritus*, were discarded as well as they were certainly secondary prey of the carabid beetles eaten by the bats, not their actual prey species (see also Galan et al., 2018). Unexpectedly, the DNA of two bat species, *Pipistrellus pipistrellus* and *Eptesicus serotinus*, was detected in five of the 284 faecal samples analysed suggesting that these species may have roosted occasionally with long‐eared bats. The prey composition in these five potentially contaminated faecal samples, however, indicated that these nontargeted bats contributed minimally to exogenous prey. Indeed, most prey species identified in these samples were moth or large flies (typical prey of long‐eared bats), whereas pipistrelles would mostly feed on small dipterans (Swift et al., 1985) and serotines on large beetles (Kervyn & Libois, 2008; Robinson & Stebbings, 1993), that were absent from these particular guano samples.

### 3.2 Molecular diet of long‐eared bats

In the curated data set of consumed prey, including the community samples for nine colonies and the smaller samples for three colonies, a total of 687 distinct MOTUs were retained, 319 (46%) of which occurred in a single guano sample. A total of 602 (88%) of these distinct MOTUs were identified taxonomically to the species level, while the remaining ones were assigned to the genus, family or order level (2%, 7% and 3%, respectively; Appendix S1). Hunted prey were mostly insects (668 MOTUs), but also included 14 spiders, four woodlice and one large springtail species. Woodlice were retained in this data set since they did occur, albeit rarely, in previous guano analyses (Kaňuch et al., 2005; Leelapaibul et al., 2009; Rydell et al., 2016). The springtail was also kept as it is possibly large enough (3 mm) to be targeted by bats or accidentally consumed during grooming (Vesterinen et al., 2013).

In the complete prey spectrum (both community and smaller samples) and as expected, the most represented insect orders were Lepidoptera and Diptera (392 and 193 MOTUs, respectively). Hemiptera, Hymenoptera, Coleoptera and Neuroptera included 14–17 species, while other detected orders (Blattodea, Dermaptera, Mecoptera, Orthoptera, Psocodea, Raphidioptera, and Trichoptera) were only represented by a handful of species. Most species identified in these faecal samples were already recorded in local (Merz, 2012) or national faunal inventories (de Jong et al., 2014), or were probably recent colonizers for the country (Andriollo, Landry, et al., 2019). Interestingly, a number of diurnal or nonflying arthropods (earwigs, orthopterans, scorpionflies, woodlice and most spiders) were only detected in faecal samples of *P*. *auritus*, while other arthropod orders were represented in comparable proportions in the diet of the three long‐eared bat species.

**FIGURE 2:**
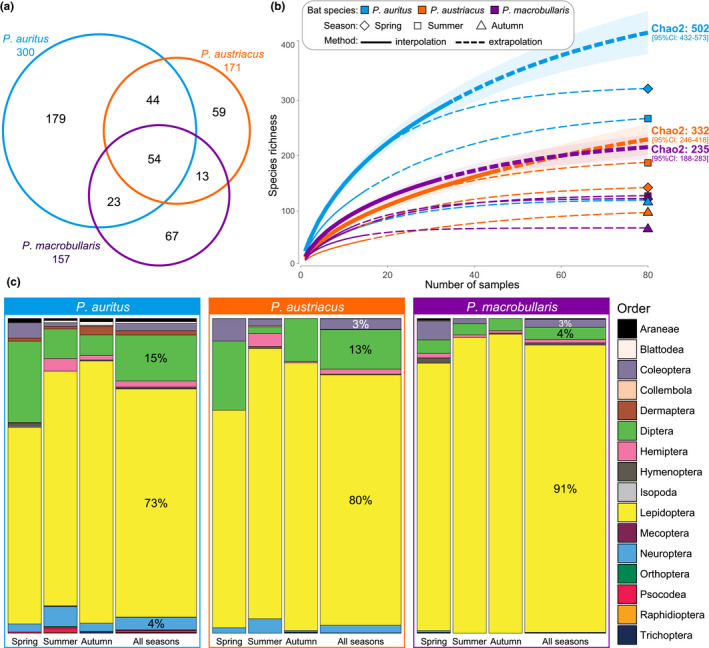
Total number of prey and their frequency of occurrence (wPOO) detected in smaller samples of guano gathered in one colony each of the three bat species. (a) Area‐proportional Euler diagram of the prey species richness detected for the three bat species. (b) Extrapolated accumulation curves indicating the number of detected prey species for each bat species and season. (c) Proportions of arthropod orders found in the diet of bats in the different seasons. Values are indicated for percentage higher than 3%

When focusing on the smaller samples gathered in the three neighbouring colonies, the diet of *P*. *auritus*, *P*. *austriacus* and *P*. *macrobullaris* included 300, 171 and 157 prey species, respectively (Figure 2a). Chao2 extrapolations from accumulation curves suggest that these numbers represent 52 to 66% of the potential prey richness, the diet of *P*. *auritus* being the most underestimated (Figure 2b). These extrapolations also indicated that 98 to 220 smaller samples per species (instead of 66) would have been necessary to detect 95% of the total species richness, which stresses the extreme diversity of the diet of all these bats. Furthermore, given the fact that a single pair of PCR primers was used to amplify prey species, all these figures can be considered as minimal spectra of the arthropods consumed by these long‐eared bats. Only 54 (12%) prey MOTUs were shared by the three bat species (Figure 2a). These shared prey species included nine very common moths (*Agrotis exclamationis*, *Agrotis ipsilon*, *Autographa gamma*, *Hoplodrina ambigua*, *Mythimna albipuncta*, *Mythimna pallens*, *Noctua pronuba*, *Nomophila noctuella* and *Xestia c*‐*nigrum*) that were detected in more than 20% of samples of each bat species. According to wPOO estimates, lepidopterans represented by far the most preferred prey in all three long‐eared bat species (73, 80 and 91% for *P*. *auritus*, *P*. *austriacus* and *P*. *macrobullaris*, respectively), followed by dipterans (15, 13 and 4%; Figure 2c). Guano samples gathered in summer and autumn invariably exhibited more Lepidoptera than the ones from spring, while the proportion of consumed dipterans varied considerably from one season or from one predator species to another. The same seasonal pattern and very similar proportions in arthropod orders were retrieved in the diet of the three bat species recovered in the community samples analysed (Appendix S4). In absolute numbers, however, less prey species were identified in the 96 community samples (Appendix S4), compared to the 186 smaller samples analysed (Figure 2a), as is expected for such unequal sampling effort (Andriollo, Gillet, et al., 2019).

Finally, a cursory review showed that all main prey orders reported so far in the menu of these bats were also recovered in our metabarcoding approach (see Section 3), with proportions very comparable to the ones reported in 17 previous studies (Appendix S5).

### 3.3 Variation of the trophic niche breadth

**FIGURE 3:**
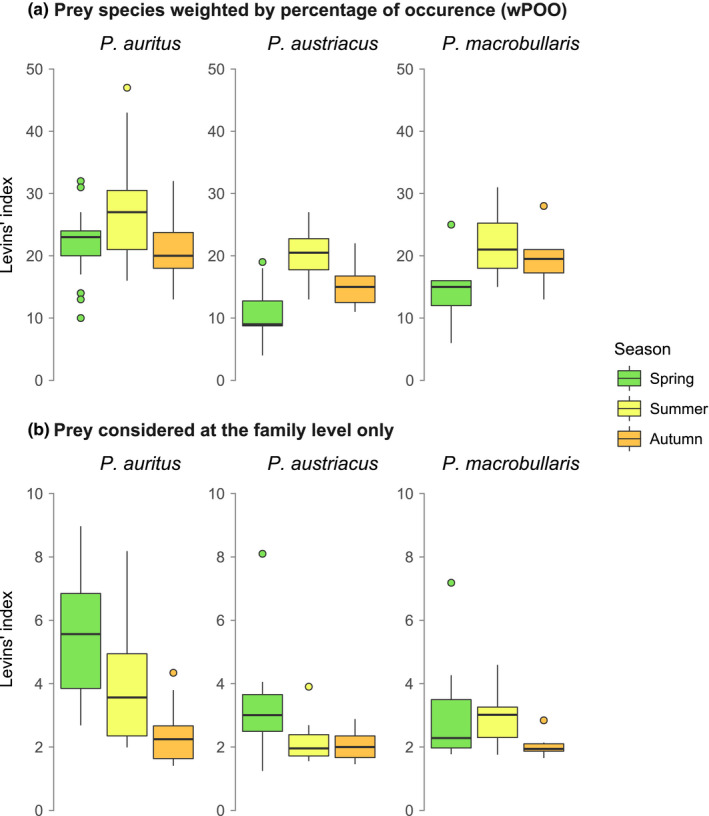
Seasonal trophic niche breadth variation (Levins’ index) measured for the three long‐eared bat species (community samples). (a) Complete data set, with all prey items kept and identified to the species level and considered as weighted occurrence data (wPOO). (b) Prey identified to the family level only (Family level)

The dietary diversity in terms of prey species exhibited the same pattern of seasonal changes for all three bat species. The trophic niche breadth of each long‐eared bat was indeed low during spring and autumn and higher during summer (Figure 3a). This pattern, however, was mostly due to an increase of consumed noctuid and geometrid moths, whose species diversity and abundance peak in summer (Altermatt, 2010). Indeed, when considering prey identified at the family level (Figure 3b), niche breadth was actually decreasing throughout the year for the three bat species. This implies that the predators consumed a restricted taxonomic range of arthropods during the summer and fall seasons. Regarding specific amplitude of the diet, *P*. *auritus* consistently exhibited a broader trophic niche than *P*.* austriacus* and *P. macrobullaris* irrespective of the season (ANOVA; *p* < .001; Figure 3a,b), or whether we focused on smaller or community samples (results not shown). This pattern is not only due to a higher number of colonies of *P. auritus* evaluated (Figure 1), as the Levins’ index measured for smaller samples (i.e., with a same effort of one analysed colony per species) throughout all the sampling period was also higher in *P. auritus* (34.4 ± 9.2) compared to *P. austriacus* (22.7 ± 8.4) or *P. macrobullaris* (22.0 ± 9.5) (Kruskal‐Wallis test; *p* < .005).

### 3.4 Variation in trophic niche overlap

**FIGURE 4:**
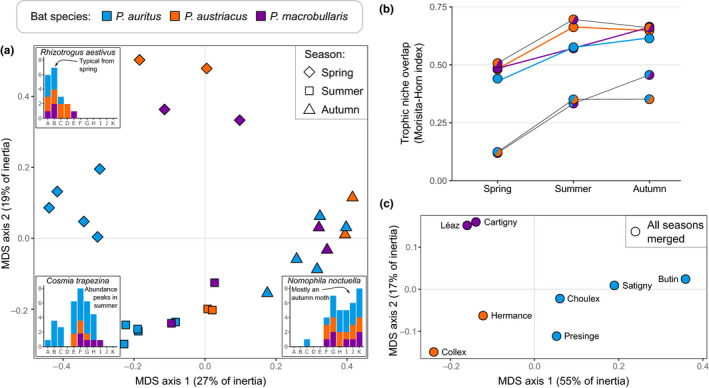
Trophic niche overlap (Morisita‐Horn measure) among *Plecotus* species, colonies and seasons (community samples). (a) Multidimensional scaling (MDS) of trophic niche overlap calculated among faecal samples collected in different colonies and across seasons. Each bat species is represented by a different color and each season by a different symbol. Three inset bar plots represent the observed phenology of prey species appearing in the diet of *Plecotus* bats and characteristic of specific sampling periods. (b) Trophic niche overlap measured throughout three periods of the year among the three long‐eared bat species, and within colonies at the intraspecific level. For each species and pairwise comparison, the lowest values of niche overlap are observed during spring. (c) MDS of trophic niche overlap calculated among faecal samples collected in different colonies regardless of season. Because the colony from Sappey was very distinct owing to its much higher location (Andriollo, Landry, et al., 2019). it was omitted from this analysis

The diet of the three species of long‐eared bats were more similar within a given season than for same‐species comparisons made across seasons (PERMANOVA across all seasons; *p* < .005; Appendix S6), as illustrated by the measure of niche overlap among the different bat roosts (Figure 4a). This was, however, less obvious in spring when the diet from each colony was more dispersed in the MDS representation, particularly those of *P. auritus* (PERMANOVA for spring only; *p* < .05; Appendix S6). This seasonal variation of diet similarity was also recovered in the PCA conducted on the prey composition of samples (Appendix S7). These similarities were driven by insect species with a marked seasonal phenology that were frequently eaten by the three bat species. For instance, the June beetle *Rhizotrogus aestivus* and the spring moth *Korscheltellus lupulinus* were characteristic of the spring samples, whereas summer noctuids such as *Cosmia trapezina* and *Hoplodrina blanda* were most abundant in samples collected during summer, regardless of which predator species ate them. The late‐summer crambid *Nomophila noctuella* and the hepialid *Triodia sylvina* (Poltavsky, 2014; Robineau et al., 2007) were accordingly mostly retrieved in guano samples collected in autumn (Appendix S7).

Dietary similarities were unequal among species and season (Figure 4b). Measures of trophic niche overlap among bat species during spring were indeed lower than those during summer and autumn. The pair *P. austriacus* and *P. macrobullaris* systematically exhibited greater niche overlap than they did with *P. auritus*, comparable to the pattern of diet overlaps measured among colonies within species. The largest values of trophic niche overlap were observed between *P. austriacus* and *P. macrobullaris* (mean value across the three seasons: 0.62). *P. auritus* exhibited much lower niche overlap with *P. austriacus* (0.28) and *P. macrobullaris* (0.30). All values of overlap were statistically higher than expected by chance (*p* < .01).

### 3.5 Relationship between ecological traits of predators and prey

Habitat preferences of the consumed arthropods could be estimated for 607 (88%) MOTUs identified to the species or genus level. The nonevaluated items were either MOTUs identified only to the order or family or few species for which no specific ecological traits could be found in the literature (Appendix S2).

Fourth‐corner statistics indicated that ecological traits of prey significantly (*p* < .01) differed from one bat species to another. *P. auritus* tended to consume prey issued from lowland cluttered habitats (woodlands, hedgerows) rather than from open or mountainous areas (such as meadows and lawns, slopes, screes and rocky areas) and was also positively and significantly associated to diurnality or flightlessness of prey (Table 1). *P. macrobullaris* exhibited opposite preferences in terms of feeding habitats, as it preyed preferentially on arthropods living in open habitats and in dry and mountainous areas (e.g., screes, slopes). Finally, *P. austriacus* showed no marked preference for specialized prey, and fourth‐corner statistics indicated that it was only positively associated to larger and ubiquitous prey species, and negatively so to prey typical of closed habitats. Finally, no direct relationship was observed between the habitat found around the different bat colonies and the habitat of the prey recovered in faeces (Table 1).

### 3.6 Typical prey species

The RLQ analysis (Appendix S8) indicated that the consumed arthropods consistently associated with faecal samples of *P. auritus* included many nonflying (spiders, woodlice, cockroaches, ground beetles, earwigs) or diurnal arthropods such as syrphid flies. Its diet was also associated to neuropterans and several tipulids living in woodlands. Forest tachinids were also detected (e.g., *Cyzenis albicans* and *Eloceria delecta*), although these parasitic flies could be secondary prey contained in moth larvae eaten by bats. Moth species also associated to *P. auritus* included geometrids (e.g., *Camptogramma bilineata* and *Operophtera brumata*) commonly found in woodlands or urban areas, noctuids (e.g., *Anorthoa munda*, *Dichonia aprilina*, *Tiliacea citrago* and *Amphipyra* spp.) typical of deciduous woodlands, and pest tortricid species (e.g., *Archips xylosteana* and *Cydia pomonella*) thriving in orchards or woodlands.

The ubiquitous prey identified in the diet of *P. austriacus* included flies from cultivated lands, such as the chloropid *Thaumatomyia notata* that lives in agricultural fields, meadows and grasslands, the muscid *Musca autumnalis*, a pullulating pest species for cattle and horses, and the calliphorid *Pollenia pediculata* notably found in urban areas. Moth species typical for *P. austriacus* included the noctuids *Caradrina clavipalpis* and *Mamestra brassicae* that can be found in a variety of open habitats.

Finally, the diet of *P. macrobullaris* was characterized by many prey species typically associated to mountainous areas such as dry meadows and lawns or sunny slopes and screes, including the noctuids *Euxoa aquilina* and *Bryophila domestica*, and the geometrids *Gnophos furvata*, *Hemistola chrysoprasaria*, *Horisme radicaria* and *Nychiodes obscuraria*.

## 4 DISCUSSION

Co‐occurring cryptic species constitute ideal models to investigate mechanisms of trophic niche partitioning with fine‐grained resolution. Previous attempts to unravel the diet of sympatric bat species have been carried using DNA metabarcoding, but all either focused on distantly‐related taxa (Emrich et al. 2014; Vesterinen et al., 2018), or on geographically‐distant or time‐limited samplings (Arrizabalaga‐Escudero et al., 2018; Novella‐Fernandez et al., 2020; Razgour et al., 2011). The present study is the first of its kind to characterize the diet of three cryptic long‐eared bats living in narrow geographic vicinity, and to access with high resolution their menu of arthropods throughout an entire season of activity. Results obtained from this design indicate that these closely‐related bats share most of their diet, but exploit distinct foraging habitats at critical periods of the year. Furthermore, marked differences in habitat use and hunting strategies of these bats are indirectly revealed through the analysis of ecological traits of their prey.

### 4.1 Sympatric bats share major components of their diet

High‐resolution techniques are essential for studying the diet composition of insectivorous bats, since many species exhibit similar overall diets when prey resources are identified to the ordinal level (Vesterinen et al., 2018). This is exemplified with the long‐eared bats which are all specialized hunters targeting tympanate moths. We confirmed here that lepidopterans represent the bulk (73%–91%) of the species identified in their diet, followed by dipterans (4%–15%), while all other arthropod groups represented less than four percent of the prey species detected (Figure 2c). These proportions corroborate previous metabarcoding studies (Alberdi et al., 2012; Razgour et al., 2011; Vesterinen et al., 2018) and microhistological analyses of faeces (Andreas, 2002; Ashrafi et al., 2011; Motte, 2011; Robinson, 1990). This concordance among independent studies (see Appendix S5) also suggests that potential recovery biases introduced by the selected primers to amplify arthropods (Zeale et al., 2011) had a limited impact, if any, on the diet assessment of these bats. As all long‐eared bats share this highly specialized menu, our observations also corroborates the high overall similarities of hunting strategies predicted from their striking phenotypic resemblance. Despite such a strong preference for moths, species‐level identification of prey indicated that the diet of these bats was much diversified, with over 680 distinct arthropod MOTUs identified in their faeces (Appendix S1). Only one fifth of moth species appeared in the menu of all three long‐eared bats (Figure 2a) but several common noctuids (*Agrotis* spp., *Mythimna* spp., *Noctua* spp.) were frequently eaten by all three bat species. These abundant and widespread taxa are also frequently hunted by other insectivorous bats and may represent an important component of their diet (Arrizabalaga‐Escudero et al., 2018). These shared prey species were particularly common in the summer diet of long‐eared bats, suggesting that the three species had an opportunistic feeding behaviour to exploit this temporally abundant trophic resource (Arlettaz, 1996; Clare et al., 2011; Cohen et al., 2020). To support such a plastic and opportunistic hunting behaviour of all three species of long‐eared bats, we also showed that the prey content of guano sampled at various times of the year was more similar within any given season (i.e., regardless the identity of the predator) rather than within one bat species across seasons (Figure 4a).

### 4.2 Overlap in trophic niche change with the seasons

Despite parallel shifts in the menu of these three species of bats, levels of trophic niche overlap among them vary across seasons and provided crucial information to understand niche partitioning in long‐eared bats. Trophic niche overlap was lower during spring and then increased notably in summer and early autumn (Figure 4b). This relationship is somewhat counterintuitive as the moth diversity (the main prey of these bats) is also at its highest during the latter seasons (Altermatt, 2010) giving more opportunities to these bats to exploit different resources. Instead, during these summer months, few abundant moths appeared in the menu of all three bat species resulting in a temporary increase in niche overlap*. *This pattern suggests that few, very common insects may provide nearly unlimited food resources during short periods of time, temporarily alleviating the need for niche partitioning. Conversely, the greater partition of trophic niche mostly occurred during spring, when prey availability was certainly lower, both in terms of diversity and abundance. At this time of the year, all three bat species appear to feed on a more diverse range of invertebrates (Figure 2 and Figure 3), including more beetles which were virtually absent from their autumn diet.

Consistent with the predictions of niche partitioning hypothesis, we suggest that the lowest niche overlap observed in spring could have evolved as a result of stronger selective pressure over food exploitation, that is, when availability of insect prey is more limiting. Other highly similar, sibling species showed the same opportunistic behaviour to feed on locally and temporally abundant prey, whereas they relied on partitioned resources at other times of the year (Arlettaz, 1996). This seasonal constraint probably applies more generally to all insectivorous bats from temperate regions exploiting insects with strongly marked seasonal phenologies. Such seasonal niche partitioning can only be detected if the sampling regime covers most of the life cycle of these bats, whereas it could be overlooked with designs focusing on narrower time scales, as has been done in many comparative studies (Arrizabalaga‐Escudero et al., 2018; Roswag, Becker, Drangusch, et al., 2018).

### 4.3 Sympatric long‐eared bats exploit different foraging habitats

Integrating the ecological traits of prey through RLQ analyses of the diet (Appendix S8) suggested the existence of marked differences in the preferred hunting grounds of the three bat species. In particular, we found that prey from closed habitats such as woodlands and hedgerows were overrepresented in the diet of *P. auritus*, whereas that of *P. macrobullaris* included more prey species living in open and rocky or mountainous areas, consistently with the alpine habits of this bat (Alberdi et al.,^2013^, ^2014^). Habitat preferences of the prey consumed by *P. austriacus* were lying between those two extreme patterns, for which the only significant relationship was a positive link to ubiquitous insects and a negative one to those from closed habitats (Table 1). The two sister species *P. auritus* and *P. macrobullaris* appeared to partition their trophic niche through the exploration of distinct hunting grounds, while *P. austriacus* was more generalist with no marked habitat preferences.

These results also shed light on the different trophic niche breadths observed in the three long‐eared bat species, which was higher in *P. auritus* when compared to the other two bat species (Figure 3a). This is not unexpected as *P. auritus* has a wide geographic distribution and is thus adapted to hunt in a variety of habitats, while *P. macrobullaris* appears to be restricted to more specific habitats available over a much smaller geographic area (Alberdi et al., 2012, 2014; Ashrafi et al., 2011; Benda et al., 2006). In this respect, it is perhaps more surprising to find also *P. austriacus* with a comparatively narrow trophic niche, as it is also very widespread in Europe. Its diet in the highly anthropized Geneva region might, however, be unrepresentative for the species elsewhere, as it exploits locally species‐poor habitats such as agricultural lands (Gilliéron et al., 2015).

Interestingly, the presence of particularly well‐forested habitats or of grasslands in the vicinity of a colony did not correlate with an abundance of woodland or of open‐habitat prey in the diet of the bats living in these colonies (Table 1). For instance, while urban areas, lawns and grasslands tended to be overrepresented around colonies of *P. auritus*, their preferred prey were still woodland arthropods. Conversely, there was a marked preference of *P. macrobullaris* for arthropods living in lawns and mountain slopes, despite the availability of extensive forests and shrubs near the two sampled colonies. These observations strongly suggest that the distinct hunting habitats inferred by our RLQ approach truly reflect habitat selection by the different bat species. These indirect inferences of foraging habitats issued for the prey spectrum are further corroborated by previous studies based on direct, but more labor‐intensive radiotracking data (Alberdi et al., 2012; Preatoni et al., 2011; Razgour et al., 2011). The indirect, RLQ approach has thus a strong potential to be applied for reconstructing the foraging ecology of elusive species without the need to capture or actually track them in their natural habitat. Finally, the inferred differences in hunting habitats also corroborates a growing number of studies demonstrating that habitat selection could be a major mechanism for resource partitioning in European insectivorous bats, even when those bats are morphologically highly similar and are syntopic (Arlettaz, 1999; Arrizabalaga‐Escudero et al., 2018; Nicholls & Racey, 2006).

### 4.4 Long‐eared bats adopt distinct foraging strategies

The analysis of prey traits does not only provide information on hunting habitats of their predators, but also suggest that the latter use different hunting techniques. Prey size and locomotion type differed among long‐eared bats (Table 1), as *P. austriacus* tended to feed on larger moths *(p* < .02), while diurnal taxa (e.g., syrphid flies) or nonflying groups (e.g., carabid beetles, spiders and woodlice) were significantly more targeted by *P. auritus* when compared to the other species. These latter prey categories are probably gleaned by bats from solid surfaces, not in flight (Anderson & Racey, 1991). This hunting technique appears to be a more common in *P. auritus* than in *P. austriacus* (Andreas, 2002; Bauerová, 1982; Beck, 1995), whereas nonflying prey was rarely reported in the diet of *P. macrobullaris* (Ashrafi et al., 2011).

Such gleaning behaviour has been classically invoked to account for the presence of wings of diurnal lepidopterans under bat roosts (Barataud, 1990; Meineke, 1991; Motte, 2011). Yet, during over ninety visits of long‐eared bat roosts throughout the year, we only recorded decayed wings of butterflies (*Aglais urticae* and *Inachis io*) in two occasions (under colonies occupied by *P. auritus*). This paucity of butterfly remains in the diet of *Plecotus* bats is corroborated by the metabarcoding approach since no butterfly sequence was identified in the guano samples, despite the high number of other lepidopterans (Appendix S1). Previous molecular analyses of *Plecotus* faeces recorded a single butterfly (*Argynnis paphia*) out of 160 lepidopteran species identified (Vesterinen et al., 2018), or none at all (Alberdi et al., 2012; Razgour et al., 2011; Roswag, Becker, & Encarnação, 2018) stressing that this pattern is a general one. Potential amplification bias can be excluded as many diurnal lepidopterans are commonly recovered in faecal analyses of bats involving the same primer pair (Bohmann et al., 2011; Vesterinen et al., 2018). We suggest that wing remains of butterfly retrieved in attics occupied by long‐eared bats might not reflect insects preyed by them, but could have been eaten by other animals such as spiders or rodents (Olofsson et al., 2011; Wiklund et al., 2008) living in the same attics.

### 4.5 Bat guano may serve for ecosystem monitoring

According to the prey diversity recovered in guano samples, long‐eared bats appeared to be remarkable samplers of the local nocturnal entomofauna. Although we certainly uncovered a modest portion of the total dietary diversity of these bats (Figure 2b), we retrieved more than a fifth of all moth species known from the area sampled, including 17 that were new occurrences for this well‐studied region (Andriollo, Landry, et al., 2019; Merz, 2012). Additionally, six neuropteran species found in the guano of long‐eared bats were not listed among the 26 known to occur in the Geneva province (Andriollo et al., 2016; Hollier, 2012), suggesting again that metabarcoding the bat guano is potentially a valuable method for indirect biodiversity assessment, provided that reliable reference databases of prey exist. In particular, bat guano can be used to monitor the presence of economically‐relevant species such as *Calliphora* and *Lucilia* flies, vectors of diseases for human and cattle, or the alien fruit fly *Drosophila suzukii*, a major pest of economic concern for fruit crops (Calabria et al., 2012; Mazzi et al., 2017).

Furthermore, as insectivorous bats are known to selectively shift their diet to feed on pest species (see above and Baroja et al., 2019; Blažek et al., 2021; Cohen et al., 2020; Kolkert et al., 2020), they prevent major agricultural losses through insect control (Boyles et al., 2011; Brown et al., 2015; Kunz et al., 2011). Although the contribution of long‐eared bats to pest control has not been quantified, we noticed that 57 species considered as agricultural pests were identified in their faeces. In particular, the noctuids *Agrotis exclamationis*, *Agrotis ipsilon*, and *Helicoverpa armigera* that are known to cause major damages to crops were detected in 61, 43, and 22% of samples, respectively, suggesting a very common consumption of these pests. These are good reasons to promote conservation efforts to maintain bats in rural areas.

### AUTHOR CONTRIBUTIONS

T.A. and M.R. conceived and designed the experiments and conducted the sampling. T.A. and J.R.M. performed the laboratory work. T. A. analysed the data and wrote the first draft. T.A., M.R. and J.R.M. contributed to the final manuscript.

## Supporting information

Supplementary Material

## Data Availability

DNA sequences of prey with complete sampling information and taxonomic assignations are available on Zenodo: https://doi.org/10.5281/zenodo.5076026 Matrices of ecological traits for all metabarcoded prey species and traits of predators are provided in the Supporting Information file, along with bibliographic references.
