# Supplementary material for: Food for everyone: Differential feeding habits of cryptic bat species inferred from DNA metabarcoding
Source: Mol Ecol. 2021 Jul 23;30(18):4584–600. doi: 10.1111/mec.16073 (PMC8518853; doi:10.1111/mec.16073)
Supplement: Supplementary file 1 — Supplementary Material [file MEC-30-4584-s001.pdf]

MOLECULAR ECOLOGY

Supplemental Information for:

Food for everyone: differential feeding habits of cryptic bat species inferred from DNA metabarcoding

Tommy Andriollo, Johan R. Michaux & Manuel Ruedi

Table of Contents:

**Appendix 1. Identity and occurrence of prey species**	Pages 2–12
**Appendix 2. Prey traits table**	Pages 13–27
**Appendix 3. Predator traits table**	Page 28
**Appendix 4. Prey composition for community samples**	Page 28
**Appendix 5. Principal component analysis of long-eared bats' diet reported in various studies**	Page 29
**Appendix 6. Analysis of variance, using residual randomization permutation procedure**	Page 30
**Appendix 7. Principal component analysis of seasonal prey composition of diet**	Page 31
**Appendix 8. RLQ analysis of prey habitat traits and their occurrence in the diet of the different *Plecotus* species**	Page 32

Appendix 1. Identity and occurrence of prey species

Taxonomic list of the 687 MOTUs recovered in fecal material from the nine *Plecotus* breeding colonies, summarized for each bat species. The number of occurrences in community samples versus small replicates is given, followed by the percentage of the samples (per bat species) in which the MOTU was recovered.

Class	Order	Family	MOTU	*P. auritus* (n = 53/66)	*P. austriacus* (n = 22/66)	*P. macrobullaris* (n = 21/54)
Arachnida	Araneae	Agelenidae	*Coelotes terrestris*	2 / 0 (2%)	0 / 0 (0%)	0 / 0 (0%)
		Amaurobiidae	*Amaurobius fenestralis*	2 / 0 (2%)	0 / 0 (0%)	0 / 0 (0%)
		Anyphaenidae	*Anyphaena accentuata*	5 / 3 (7%)	0 / 0 (0%)	0 / 0 (0%)
		Araneidae	*Gibbaranea gibbosa*	2 / 1 (3%)	0 / 0 (0%)	0 / 0 (0%)
			Nuctenea umbratica	3 / 0 (3%)	0 / 0 (0%)	0 / 0 (0%)
		Clubionidae	*Clubiona corticalis*	1 / 2 (3%)	0 / 0 (0%)	0 / 0 (0%)
			Clubiona pallidula	1 / 1 (2%)	0 / 0 (0%)	0 / 0 (0%)
		Eutichuridae	*Cheiracanthium mildei*	1 / 0 (1%)	0 / 0 (0%)	0 / 0 (0%)
		Philodromidae	*Philodromus aureolus*	0 / 0 (0%)	1 / 0 (1%)	0 / 0 (0%)
			Philodromus buxi	0 / 0 (0%)	0 / 0 (0%)	0 / 1 (1%)
			Philodromus margaritatus	1 / 0 (1%)	0 / 0 (0%)	0 / 0 (0%)
		Salticidae	*Salticus zebraneus*	0 / 1 (1%)	0 / 0 (0%)	0 / 0 (0%)
		Thomisidae	*Diaea dorsata*	1 / 0 (1%)	0 / 0 (0%)	0 / 0 (0%)
			Xysticus lanio	0 / 1 (1%)	0 / 0 (0%)	0 / 0 (0%)
Entognatha	Collembola	Sminthuridae	*Sminthurus viridis*	0 / 0 (0%)	0 / 1 (1%)	0 / 0 (0%)
Insecta	Blattodea	Ectobiidae	*Ectobius sylvestris*	5 / 0 (4%)	0 / 0 (0%)	0 / 1 (1%)
			Ectobius vittiventris	7 / 5 (10%)	1 / 1 (2%)	0 / 0 (0%)
	Coleoptera	Carabidae	*Amara similata*	0 / 1 (1%)	0 / 0 (0%)	0 / 0 (0%)
			Bembidion quadrimaculatum	1 / 0 (1%)	0 / 0 (0%)	0 / 0 (0%)
			Harpalus rufipes	0 / 0 (0%)	0 / 0 (0%)	2 / 3 (7%)
		Cerambycidae	*Acanthocinus griseus*	0 / 0 (0%)	0 / 0 (0%)	0 / 1 (1%)
			Mesosa nebulosa	0 / 1 (1%)	0 / 0 (0%)	0 / 0 (0%)
		Cleridae	*Opilo mollis*	1 / 1 (2%)	0 / 0 (0%)	0 / 0 (0%)
		Curculionidae	*Curculio glandium*	0 / 1 (1%)	0 / 0 (0%)	0 / 0 (0%)
			Dorytomus longimanus	1 / 1 (2%)	0 / 0 (0%)	0 / 0 (0%)
		Elateridae	*Athous* sp.	0 / 0 (0%)	0 / 0 (0%)	0 / 2 (3%)
			Stenagostus rhombeus	0 / 0 (0%)	0 / 0 (0%)	0 / 2 (3%)
		Melyridae	*Dasytes aeratus*	0 / 1 (1%)	0 / 1 (1%)	0 / 0 (0%)
		Scarabaeidae	*Amphimallon majale*	0 / 1 (1%)	1 / 4 (6%)	0 / 3 (4%)
			Rhizotrogus aestivus	8 / 11 (16%)	8 / 13 (24%)	3 / 1 (5%)
			Serica brunnea	3 / 3 (5%)	4 / 0 (5%)	2 / 4 (8%)
		Staphylinidae	*Eusphalerum signatum*	1 / 0 (1%)	0 / 0 (0%)	0 / 0 (0%)
		Unknown	Coleoptera sp.	1 / 1 (2%)	0 / 0 (0%)	0 / 0 (0%)
	Dermaptera	Forficulidae	*Chelidurella guentheri*	5 / 4 (8%)	0 / 0 (0%)	0 / 0 (0%)
			Forficula auricularia	11 / 10 (18%)	0 / 0 (0%)	0 / 0 (0%)
	Diptera	Anisopodidae	*Sylvicola cinctus*	0 / 4 (3%)	0 / 0 (0%)	0 / 0 (0%)
			Sylvicola stackelbergi	0 / 3 (3%)	0 / 0 (0%)	1 / 0 (1%)
		Anthomyiidae	*Anthomyia* sp.	0 / 1 (1%)	0 / 0 (0%)	0 / 0 (0%)
			Botanophila fugax	0 / 2 (2%)	0 / 0 (0%)	0 / 0 (0%)
			Delia florilega	1 / 0 (1%)	0 / 0 (0%)	0 / 0 (0%)
			Delia platura	1 / 1 (2%)	0 / 1 (1%)	0 / 0 (0%)
			Delia radicum	0 / 1 (1%)	0 / 0 (0%)	0 / 0 (0%)
			Hydrophoria silvicola	2 / 1 (3%)	0 / 0 (0%)	0 / 0 (0%)
			Pegoplata infirma	1 / 0 (1%)	0 / 0 (0%)	0 / 0 (0%)
		Bibionidae	*Dilophus febrilis*	0 / 1 (1%)	0 / 0 (0%)	0 / 0 (0%)
		Calliphoridae	*Bellardia viarum*	1 / 0 (1%)	0 / 0 (0%)	0 / 0 (0%)
			Bellardia vulgaris	1 / 0 (1%)	0 / 0 (0%)	0 / 0 (0%)
			Calliphora vicina	1 / 2 (3%)	0 / 0 (0%)	0 / 0 (0%)
			Calliphora vomitoria	1 / 0 (1%)	0 / 0 (0%)	0 / 0 (0%)
			Lucilia caesar	0 / 1 (1%)	0 / 0 (0%)	0 / 0 (0%)
			Lucilia sericata	1 / 3 (3%)	0 / 1 (1%)	0 / 0 (0%)
			Pollenia hungarica	5 / 2 (6%)	0 / 1 (1%)	0 / 0 (0%)
			Pollenia pediculata	4 / 0 (3%)	0 / 12 (14%)	0 / 1 (1%)
			Pollenia rudis	6 / 1 (6%)	0 / 4 (5%)	0 / 2 (3%)
			Pollenia vagabunda	1 / 0 (1%)	0 / 0 (0%)	0 / 0 (0%)
			Calliphoridae sp.	2 / 0 (2%)	0 / 0 (0%)	0 / 0 (0%)

Class	Order	Family	MOTU	***P. auritus*** **(n = 53/66)**	***P. austriacus*** **(n = 22/66)**	***P. macrobullaris*** **(n = 21/54)**
		Carnidae	*Meoneura neottiophila*	0 / 0 (0%)	1 / 0 (1%)	0 / 0 (0%)
		Cecidomyiidae	*Asteromyia* sp.	1 / 0 (1%)	0 / 0 (0%)	0 / 0 (0%)
			Mayetiola destructor	0 / 1 (1%)	0 / 0 (0%)	0 / 0 (0%)
			Cecidomyiidae sp. 1	0 / 1 (1%)	0 / 0 (0%)	0 / 0 (0%)
			Cecidomyiidae sp. 2	0 / 1 (1%)	0 / 0 (0%)	0 / 0 (0%)
			Cecidomyiidae sp. 3	1 / 0 (1%)	0 / 0 (0%)	0 / 0 (0%)
		Ceratopogonidae	*Culicoides chiopterus*	1 / 0 (1%)	0 / 0 (0%)	0 / 0 (0%)
		Chironomidae	*Conchapelopia melanops*	1 / 0 (1%)	0 / 0 (0%)	0 / 0 (0%)
			Orthocladius rubicundus	0 / 0 (0%)	0 / 1 (1%)	0 / 0 (0%)
			Paratrichocladius rufiventris	0 / 1 (1%)	0 / 0 (0%)	0 / 0 (0%)
			Polypedilum convictum	0 / 1 (1%)	0 / 0 (0%)	0 / 0 (0%)
			Polypedilum sp.	1 / 0 (1%)	0 / 0 (0%)	0 / 0 (0%)
			Chironomidae sp. 1	0 / 0 (0%)	1 / 0 (1%)	0 / 0 (0%)
			Chironomidae sp. 2	1 / 0 (1%)	0 / 0 (0%)	0 / 0 (0%)
		Chloropidae	*Thaumatomyia notata*	0 / 0 (0%)	2 / 1 (3%)	0 / 0 (0%)
		Culicidae	*Culex pipiens*	0 / 1 (1%)	0 / 0 (0%)	0 / 0 (0%)
		Drosophilidae	*Drosophila melanogaster*	0 / 1 (1%)	0 / 0 (0%)	0 / 0 (0%)
			Drosophila subobscura	0 / 1 (1%)	0 / 0 (0%)	0 / 0 (0%)
			Drosophila suzukii	1 / 0 (1%)	0 / 0 (0%)	0 / 0 (0%)
			Phortica variegata	0 / 1 (1%)	0 / 0 (0%)	0 / 0 (0%)
		Empididae	*Empis bicuspidata*	0 / 1 (1%)	0 / 0 (0%)	0 / 0 (0%)
			Empis chioptera	0 / 2 (2%)	0 / 0 (0%)	0 / 0 (0%)
			Empis ciliata	1 / 1 (2%)	0 / 0 (0%)	0 / 0 (0%)
			Empis stercorea	1 / 0 (1%)	0 / 0 (0%)	0 / 0 (0%)
			Empis tessellata	1 / 0 (1%)	0 / 0 (0%)	0 / 0 (0%)
			Empis trigramma	1 / 1 (2%)	0 / 0 (0%)	0 / 0 (0%)
			Empis variegata	3 / 4 (6%)	0 / 0 (0%)	0 / 0 (0%)
			Hilara sp.	0 / 0 (0%)	0 / 1 (1%)	0 / 0 (0%)
			Rhamphomyia crassirostris	0 / 2 (2%)	0 / 0 (0%)	0 / 0 (0%)
			Rhamphomyia sp.	0 / 2 (2%)	0 / 0 (0%)	0 / 0 (0%)
			Empididae sp. 1	0 / 1 (1%)	0 / 0 (0%)	0 / 0 (0%)
			Empididae sp. 2	1 / 2 (3%)	0 / 0 (0%)	0 / 0 (0%)
			Empididae sp. 3	0 / 1 (1%)	0 / 0 (0%)	0 / 0 (0%)
		Fanniidae	*Fannia pallitibia*	2 / 0 (2%)	0 / 0 (0%)	0 / 0 (0%)
			Fannia polychaeta	0 / 1 (1%)	0 / 0 (0%)	0 / 0 (0%)
			Fannia scalaris	1 / 0 (1%)	0 / 0 (0%)	0 / 0 (0%)
			Fannia sp.	0 / 1 (1%)	0 / 0 (0%)	0 / 0 (0%)
			Fanniidae sp.	0 / 2 (2%)	0 / 0 (0%)	0 / 0 (0%)
		Heleomyzidae	*Suillia bicolor*	0 / 0 (0%)	0 / 0 (0%)	0 / 2 (3%)
			Heleomyzidae sp.	0 / 1 (1%)	0 / 0 (0%)	0 / 0 (0%)
		Lauxaniidae	Lauxaniidae sp. 1	1 / 0 (1%)	0 / 0 (0%)	0 / 0 (0%)
			Lauxaniidae sp. 2	0 / 2 (2%)	0 / 0 (0%)	0 / 0 (0%)
		Limoniidae	*Antocha vitripennis*	1 / 0 (1%)	0 / 0 (0%)	0 / 0 (0%)
			Cheilotrichia cinerascens	1 / 0 (1%)	0 / 0 (0%)	0 / 0 (0%)
			Dicranomyia tristis	1 / 1 (2%)	0 / 0 (0%)	0 / 0 (0%)
			Dicranomyia sp.	2 / 4 (5%)	0 / 2 (2%)	0 / 0 (0%)
			Helius flavus	0 / 0 (0%)	0 / 0 (0%)	1 / 1 (3%)
			Limonia nigropunctata	1 / 0 (1%)	0 / 0 (0%)	0 / 0 (0%)
			Limonia nubeculosa	8 / 3 (9%)	0 / 0 (0%)	0 / 0 (0%)
			Limonia phragmitidis	0 / 0 (0%)	1 / 0 (1%)	0 / 0 (0%)
			Limonia sp.	1 / 2 (3%)	1 / 1 (2%)	0 / 0 (0%)
			Metalimnobia bifasciata	0 / 1 (1%)	0 / 0 (0%)	0 / 0 (0%)
			Phylidorea ferruginea	1 / 0 (1%)	1 / 0 (1%)	0 / 0 (0%)
			Rhipidia maculata	3 / 0 (3%)	0 / 0 (0%)	0 / 0 (0%)
		Lonchaeidae	Lonchaeidae sp.	1 / 0 (1%)	0 / 0 (0%)	0 / 0 (0%)
		Muscidae	*Atherigona orientalis*	0 / 1 (1%)	0 / 0 (0%)	0 / 0 (0%)
			Coenosia testacea	0 / 0 (0%)	1 / 0 (1%)	0 / 0 (0%)
			Hebecnema fumosa	0 / 1 (1%)	0 / 0 (0%)	0 / 0 (0%)
			Helina abdominalis	0 / 1 (1%)	0 / 0 (0%)	0 / 0 (0%)
			Helina evecta	0 / 1 (1%)	0 / 0 (0%)	0 / 0 (0%)
			Helina impuncta	11 / 1 (10%)	0 / 0 (0%)	0 / 2 (3%)
			Helina lasiophthalma	1 / 0 (1%)	0 / 1 (1%)	0 / 0 (0%)
			Helina pertusa	1 / 0 (1%)	0 / 0 (0%)	0 / 0 (0%)
			Helina reversio	1 / 0 (1%)	0 / 0 (0%)	0 / 0 (0%)

Class	Order	Family	MOTU	*P. auritus* (n = 53/66)	*P. austriacus* (n = 22/66)	*P. macrobullaris* (n = 21/54)
			Helina sp.	1 / 0 (1%)	0 / 0 (0%)	0 / 0 (0%)
			Hydrotaea irritans	5 / 0 (4%)	0 / 0 (0%)	0 / 0 (0%)
			Musca autumnalis	6 / 3 (8%)	2 / 14 (18%)	0 / 0 (0%)
			Muscina levida	3 / 1 (3%)	0 / 1 (1%)	0 / 0 (0%)
			Muscina pascuorum	1 / 0 (1%)	1 / 2 (3%)	0 / 0 (0%)
			Muscina prolapsa	1 / 0 (1%)	0 / 0 (0%)	0 / 0 (0%)
			Phaonia errans	2 / 1 (3%)	0 / 0 (0%)	0 / 0 (0%)
			Phaonia pallida	1 / 0 (1%)	0 / 0 (0%)	0 / 0 (0%)
			Phaonia rufiventris	1 / 0 (1%)	0 / 0 (0%)	0 / 0 (0%)
			Phaonia subventa	2 / 0 (2%)	0 / 0 (0%)	0 / 0 (0%)
			Phaonia trimaculata	1 / 0 (1%)	0 / 0 (0%)	0 / 0 (0%)
			Phaonia tuguriorum	3 / 0 (3%)	0 / 0 (0%)	0 / 0 (0%)
			Phaonia sp.	1 / 0 (1%)	0 / 0 (0%)	0 / 0 (0%)
			Polietes lardarius	4 / 0 (3%)	1 / 0 (1%)	0 / 0 (0%)
			Stomoxys calcitrans	0 / 1 (1%)	0 / 0 (0%)	0 / 0 (0%)
			Muscidae sp.	0 / 0 (0%)	0 / 1 (1%)	0 / 0 (0%)
		Mycetophilidae	*Leia fascipennis*	2 / 0 (2%)	0 / 0 (0%)	0 / 0 (0%)
		Opomyzidae	*Geomyza tripunctata*	1 / 0 (1%)	0 / 0 (0%)	0 / 0 (0%)
		Psilidae	Psilidae sp. 1	1 / 0 (1%)	0 / 0 (0%)	0 / 0 (0%)
			Psilidae sp. 2	1 / 0 (1%)	0 / 0 (0%)	0 / 0 (0%)
		Rhagionidae	*Chrysopilus asiliformis*	1 / 0 (1%)	0 / 0 (0%)	0 / 0 (0%)
		Rhiniidae	*Stomorhina lunata*	1 / 0 (1%)	0 / 0 (0%)	0 / 0 (0%)
		Sarcophagidae	*Sarcophaga agnata*	1 / 0 (1%)	0 / 0 (0%)	0 / 0 (0%)
			Sarcophaga carnaria	10 / 6 (13%)	0 / 0 (0%)	0 / 0 (0%)
			Sarcophaga incisilobata	1 / 0 (1%)	0 / 0 (0%)	0 / 0 (0%)
			Sarcophaga subvicina	0 / 5 (4%)	0 / 0 (0%)	0 / 0 (0%)
			Sarcophaga sp.	0 / 1 (1%)	0 / 0 (0%)	0 / 0 (0%)
		Scathophagidae	*Scathophaga stercoraria*	2 / 0 (2%)	0 / 0 (0%)	0 / 0 (0%)
		Sciaridae	*Bradysia trivittata*	1 / 0 (1%)	0 / 2 (2%)	0 / 0 (0%)
		Simuliidae	*Simulium vernum*	0 / 0 (0%)	0 / 1 (1%)	0 / 0 (0%)
		Stratiomyidae	*Sargus bipunctatus*	2 / 0 (2%)	0 / 0 (0%)	0 / 0 (0%)
		Syrphidae	*Dasysyrphus albostriatus*	1 / 0 (1%)	0 / 0 (0%)	0 / 0 (0%)
			Episyrphus balteatus	2 / 2 (3%)	0 / 0 (0%)	0 / 0 (0%)
			Eristalis pertinax	2 / 0 (2%)	0 / 0 (0%)	0 / 0 (0%)
			Eupeodes lapponicus	1 / 0 (1%)	0 / 0 (0%)	0 / 0 (0%)
			Melanostoma mellinum/scalare	0 / 2 (2%)	0 / 0 (0%)	0 / 0 (0%)
			Melanostoma scalare	1 / 1 (2%)	0 / 0 (0%)	0 / 0 (0%)
			Meliscaeva auricollis	1 / 0 (1%)	0 / 0 (0%)	0 / 0 (0%)
			Meliscaeva cinctella	2 / 0 (2%)	0 / 0 (0%)	0 / 0 (0%)
			Parasyrphus punctulatus	0 / 1 (1%)	0 / 0 (0%)	0 / 0 (0%)
			Platycheirus scutatus	0 / 1 (1%)	0 / 0 (0%)	0 / 0 (0%)
			Syrphus ribesii	3 / 0 (3%)	0 / 0 (0%)	0 / 0 (0%)
			Syrphus torvus	3 / 0 (3%)	0 / 0 (0%)	0 / 0 (0%)
			Syrphus vitripennis	5 / 1 (5%)	0 / 0 (0%)	0 / 0 (0%)
			Syrphidae sp.	1 / 0 (1%)	0 / 0 (0%)	0 / 0 (0%)
		Tabanidae	*Atylotus loewianus*	1 / 0 (1%)	0 / 0 (0%)	0 / 0 (0%)
			Tabanus bromius	1 / 0 (1%)	0 / 0 (0%)	0 / 0 (0%)
		Tachinidae	*Allophorocera ferruginea*	1 / 0 (1%)	0 / 0 (0%)	0 / 0 (0%)
			Blepharomyia pagana	1 / 1 (2%)	0 / 0 (0%)	0 / 0 (0%)
			Blondelia nigripes	2 / 1 (3%)	0 / 0 (0%)	0 / 1 (1%)
			Cyzenis albicans	8 / 12 (17%)	2 / 2 (5%)	0 / 0 (0%)
			Eloceria delecta	5 / 3 (7%)	0 / 0 (0%)	0 / 0 (0%)
			Kirbya moerens	1 / 0 (1%)	1 / 0 (1%)	0 / 0 (0%)
			Linnaemya tessellans	1 / 1 (2%)	0 / 0 (0%)	2 / 1 (4%)
			Loewia foeda	1 / 0 (1%)	0 / 0 (0%)	0 / 0 (0%)
			Lypha dubia	2 / 1 (3%)	0 / 0 (0%)	0 / 0 (0%)
			Meigenia mutabilis	0 / 0 (0%)	0 / 1 (1%)	0 / 0 (0%)
			Ocytata pallipes	1 / 0 (1%)	0 / 0 (0%)	0 / 0 (0%)
			Oswaldia muscaria	1 / 0 (1%)	1 / 0 (1%)	0 / 0 (0%)
			Pales pavida	0 / 1 (1%)	0 / 0 (0%)	0 / 0 (0%)
			Peribaea tibialis	0 / 0 (0%)	0 / 0 (0%)	2 / 1 (4%)
			Phorocera assimilis	1 / 1 (2%)	0 / 0 (0%)	0 / 0 (0%)
			Phryno vetula	7 / 0 (6%)	0 / 1 (1%)	0 / 0 (0%)
			Phryxe vulgaris	2 / 2 (3%)	0 / 1 (1%)	2 / 1 (4%)

Class	Order	Family	MOTU	*P. auritus* (n = 53/66)	*P. austriacus* (n = 22/66)	*P. macrobullaris* (n = 21/54)
			Pseudoperichaeta nigrolineata	0 / 1 (1%)	0 / 0 (0%)	0 / 0 (0%)
			Ramonda prunaria	0 / 0 (0%)	0 / 0 (0%)	1 / 1 (3%)
			Ramonda spathulata	1 / 0 (1%)	0 / 0 (0%)	0 / 0 (0%)
			Siphona geniculata	1 / 0 (1%)	0 / 0 (0%)	0 / 0 (0%)
			Triarthria setipennis	1 / 0 (1%)	0 / 0 (0%)	0 / 0 (0%)
			Voria ruralis	0 / 1 (1%)	1 / 1 (2%)	0 / 1 (1%)
			Zaira cinerea	0 / 1 (1%)	0 / 0 (0%)	0 / 0 (0%)
			Tachinidae sp. 1	0 / 0 (0%)	0 / 1 (1%)	0 / 0 (0%)
			Tachinidae sp. 2	1 / 0 (1%)	0 / 0 (0%)	0 / 0 (0%)
			Tachinidae sp. 3	1 / 0 (1%)	0 / 0 (0%)	0 / 0 (0%)
		Tephritidae	*Anomoia purmunda*	0 / 1 (1%)	0 / 0 (0%)	0 / 0 (0%)
		Tipulidae	*Nephrotoma appendiculata*	10 / 6 (13%)	2 / 7 (10%)	1 / 0 (1%)
			Nephrotoma flavescens	0 / 0 (0%)	0 / 0 (0%)	0 / 2 (3%)
			Nephrotoma scalaris	2 / 3 (4%)	0 / 0 (0%)	0 / 1 (1%)
			Tipula fascipennis	0 / 2 (2%)	0 / 0 (0%)	0 / 0 (0%)
			Tipula flavolineata	1 / 0 (1%)	0 / 0 (0%)	0 / 0 (0%)
			Tipula fulvipennis	1 / 0 (1%)	0 / 0 (0%)	0 / 0 (0%)
			Tipula helvola	0 / 1 (1%)	0 / 0 (0%)	0 / 0 (0%)
			Tipula cf. *hortorum*	3 / 0 (3%)	0 / 0 (0%)	0 / 0 (0%)
			Tipula lateralis	2 / 0 (2%)	0 / 0 (0%)	0 / 0 (0%)
			Tipula cf. *limbata*	2 / 0 (2%)	0 / 0 (0%)	0 / 0 (0%)
			Tipula luna	1 / 0 (1%)	0 / 0 (0%)	0 / 0 (0%)
			Tipula lunata	4 / 3 (6%)	1 / 0 (1%)	0 / 0 (0%)
			Tipula cf. *magnicauda*	1 / 1 (2%)	0 / 0 (0%)	1 / 3 (5%)
			Tipula maxima	1 / 0 (1%)	0 / 0 (0%)	0 / 0 (0%)
			Tipula oleracea/recondita	8 / 10 (15%)	3 / 9 (14%)	2 / 3 (7%)
			Tipula pagana	2 / 0 (2%)	0 / 0 (0%)	0 / 0 (0%)
			Tipula paludosa	0 / 0 (0%)	0 / 0 (0%)	1 / 3 (5%)
			Tipula peliostigma	0 / 1 (1%)	0 / 0 (0%)	0 / 0 (0%)
			Tipula scripta	3 / 0 (3%)	0 / 0 (0%)	0 / 0 (0%)
			Tipula submarmorata	1 / 0 (1%)	0 / 0 (0%)	0 / 0 (0%)
			Tipula cf. *truncorum*	1 / 0 (1%)	1 / 0 (1%)	0 / 0 (0%)
			Tipula vernalis	7 / 4 (9%)	3 / 9 (14%)	0 / 0 (0%)
			Tipula sp. 1	1 / 0 (1%)	0 / 0 (0%)	0 / 0 (0%)
			Tipula sp. 2	1 / 0 (1%)	0 / 0 (0%)	0 / 0 (0%)
			Tipula sp. 3	1 / 0 (1%)	0 / 0 (0%)	0 / 0 (0%)
		Ulidiidae	*Physiphora alceae*	0 / 0 (0%)	1 / 0 (1%)	0 / 0 (0%)
		Unknown	Diptera sp. 1	0 / 1 (1%)	0 / 0 (0%)	0 / 0 (0%)
			Diptera sp. 2	0 / 2 (2%)	0 / 0 (0%)	1 / 0 (1%)
			Diptera sp. 3	0 / 0 (0%)	0 / 0 (0%)	0 / 1 (1%)
			Diptera sp. 4	0 / 1 (1%)	0 / 0 (0%)	0 / 0 (0%)
			Diptera sp. 5	1 / 0 (1%)	0 / 0 (0%)	0 / 0 (0%)
			Diptera sp. 6	0 / 1 (1%)	0 / 0 (0%)	0 / 0 (0%)
			Diptera sp. 7	1 / 0 (1%)	0 / 0 (0%)	0 / 0 (0%)
	Hemiptera	Acanthosomatidae	Acanthosomatidae sp.	0 / 0 (0%)	0 / 0 (0%)	0 / 1 (1%)
		Aphididae	*Tuberculatus querceus*	1 / 0 (1%)	0 / 0 (0%)	0 / 0 (0%)
		Aphrophoridae	*Aphrophora alni*	0 / 3 (3%)	0 / 0 (0%)	0 / 0 (0%)
		Cercopidae	*Philaenus spumarius*	0 / 1 (1%)	0 / 0 (0%)	0 / 0 (0%)
		Cicadellidae	*Iassus lanio*	6 / 5 (9%)	0 / 5 (6%)	0 / 0 (0%)
			Cicadellidae sp.	0 / 1 (1%)	0 / 2 (2%)	0 / 0 (0%)
		Miridae	*Adelphocoris lineolatus*	2 / 1 (3%)	0 / 3 (3%)	1 / 5 (8%)
			Adelphocoris seticornis	0 / 0 (0%)	0 / 1 (1%)	0 / 1 (1%)
			Lygus cf. *pratensis*	4 / 4 (7%)	2 / 5 (8%)	0 / 2 (3%)
			Neolygus viridis	1 / 0 (1%)	0 / 1 (1%)	0 / 0 (0%)
			Orthops kalmii	0 / 0 (0%)	1 / 0 (1%)	0 / 0 (0%)
			Orthotylus prasinus	1 / 0 (1%)	0 / 0 (0%)	0 / 0 (0%)
			Orthotylus viridinervis	0 / 1 (1%)	0 / 0 (0%)	0 / 0 (0%)
			Phytocoris dimidiatus	1 / 0 (1%)	0 / 0 (0%)	0 / 0 (0%)
			Phytocoris longipennis	1 / 0 (1%)	0 / 0 (0%)	0 / 0 (0%)
			Phytocoris tiliae	2 / 1 (3%)	0 / 0 (0%)	0 / 0 (0%)
			Pinalitus cervinus	0 / 0 (0%)	1 / 1 (2%)	0 / 0 (0%)
	Hymenoptera	Braconidae	*Charmon* sp.	1 / 0 (1%)	0 / 0 (0%)	0 / 0 (0%)
			Microplitis sp. 1	0 / 1 (1%)	0 / 0 (0%)	0 / 0 (0%)
			Microplitis sp. 2	1 / 0 (1%)	0 / 0 (0%)	0 / 0 (0%)

Class	Order	Family	MOTU	*P. auritus* (n = 53/66)	*P. austriacus* (n = 22/66)	*P. macrobullaris* (n = 21/54)
			Braconidae sp. 1	0 / 0 (0%)	1 / 0 (1%)	1 / 1 (3%)
			Braconidae sp. 2	0 / 0 (0%)	0 / 1 (1%)	0 / 0 (0%)
		Cynipidae	*Andricus quercusradicis*	1 / 0 (1%)	0 / 0 (0%)	0 / 0 (0%)
		Ichneumonidae	*Campoplex rothii*	0 / 0 (0%)	0 / 0 (0%)	1 / 0 (1%)
			Diphyus sp.	0 / 0 (0%)	0 / 0 (0%)	0 / 1 (1%)
			Enicospilus ramidulus	0 / 0 (0%)	0 / 1 (1%)	0 / 2 (3%)
			Ichneumonidae sp. 1	2 / 1 (3%)	0 / 0 (0%)	0 / 0 (0%)
			Ichneumonidae sp. 2	0 / 1 (1%)	0 / 0 (0%)	0 / 0 (0%)
			Ichneumonidae sp. 3	0 / 0 (0%)	1 / 0 (1%)	0 / 0 (0%)
		Perilampidae	Perilampidae sp.	1 / 0 (1%)	0 / 0 (0%)	0 / 0 (0%)
		Pteromalidae	Pteromalidae sp.	0 / 1 (1%)	0 / 0 (0%)	0 / 0 (0%)
		Tenthredinidae	*Periclista pubescens*	0 / 1 (1%)	0 / 0 (0%)	0 / 0 (0%)
		Vespidae	*Polistes dominula*	1 / 0 (1%)	0 / 0 (0%)	0 / 0 (0%)
	Lepidoptera	Adelidae	*Nematopogon swammerdamella*	0 / 2 (2%)	0 / 0 (0%)	0 / 0 (0%)
		Blastobasidae	*Blastobasis phycidella*	0 / 0 (0%)	0 / 0 (0%)	1 / 0 (1%)
		Chimabachidae	*Diurnea fagella*	1 / 0 (1%)	0 / 0 (0%)	0 / 0 (0%)
		Depressariidae	*Carcina quercana*	1 / 0 (1%)	0 / 0 (0%)	0 / 0 (0%)
			Depressaria douglasella	1 / 0 (1%)	0 / 0 (0%)	0 / 1 (1%)
		Drepanidae	*Cymatophorina diluta*	1 / 3 (3%)	0 / 0 (0%)	0 / 0 (0%)
			Habrosyne pyritoides	0 / 0 (0%)	0 / 1 (1%)	0 / 0 (0%)
			Polyploca ridens	2 / 3 (4%)	1 / 1 (2%)	0 / 0 (0%)
			Tethea ocularis	1 / 0 (1%)	0 / 0 (0%)	0 / 0 (0%)
			Tethea or	0 / 0 (0%)	1 / 0 (1%)	0 / 0 (0%)
			Thyatira batis	0 / 1 (1%)	0 / 0 (0%)	0 / 0 (0%)
		Erebidae	*Aedia funesta*	0 / 0 (0%)	0 / 1 (1%)	0 / 0 (0%)
			Arctia caja	0 / 0 (0%)	0 / 2 (2%)	3 / 3 (8%)
			Catephia alchymista	0 / 0 (0%)	1 / 1 (2%)	1 / 0 (1%)
			Catocala nupta	5 / 2 (6%)	0 / 0 (0%)	1 / 0 (1%)
			Catocala promissa	3 / 4 (6%)	1 / 0 (1%)	0 / 0 (0%)
			Catocala sponsa	5 / 4 (8%)	3 / 3 (7%)	0 / 0 (0%)
			Coscinia cribraria	0 / 0 (0%)	0 / 0 (0%)	1 / 0 (1%)
			Diacrisia sannio	0 / 0 (0%)	1 / 0 (1%)	5 / 4 (12%)
			Diaphora mendica	2 / 0 (2%)	1 / 1 (2%)	0 / 0 (0%)
			Dysgonia algira	0 / 0 (0%)	0 / 1 (1%)	0 / 0 (0%)
			Euplagia quadripunctaria	0 / 0 (0%)	0 / 0 (0%)	1 / 0 (1%)
			Euproctis chrysorrhoea	0 / 0 (0%)	1 / 0 (1%)	1 / 1 (3%)
			Herminia tarsicrinalis	1 / 0 (1%)	0 / 0 (0%)	0 / 2 (3%)
			Herminia tarsipennalis	0 / 0 (0%)	1 / 0 (1%)	0 / 0 (0%)
			Hypena proboscidalis	1 / 0 (1%)	0 / 0 (0%)	0 / 0 (0%)
			Laspeyria flexula	3 / 1 (3%)	0 / 1 (1%)	0 / 0 (0%)
			Lygephila pastinum	0 / 0 (0%)	1 / 0 (1%)	0 / 0 (0%)
			Paracolax tristalis	0 / 0 (0%)	0 / 1 (1%)	0 / 0 (0%)
			Phragmatobia fuliginosa	1 / 4 (4%)	1 / 1 (2%)	2 / 5 (9%)
			Polypogon plumigeralis	1 / 0 (1%)	0 / 0 (0%)	0 / 0 (0%)
			Polypogon strigilata	0 / 1 (1%)	0 / 0 (0%)	0 / 0 (0%)
			Rivula sericealis	4 / 2 (5%)	0 / 1 (1%)	2 / 0 (3%)
			Scoliopteryx libatrix	4 / 1 (4%)	2 / 3 (6%)	0 / 0 (0%)
			Spilarctia lutea	1 / 1 (2%)	3 / 0 (3%)	1 / 0 (1%)
			Spilosoma lubricipeda	1 / 0 (1%)	1 / 0 (1%)	2 / 1 (4%)
			Trisateles emortualis	0 / 1 (1%)	0 / 0 (0%)	0 / 0 (0%)
			Tyta luctuosa	0 / 0 (0%)	0 / 0 (0%)	1 / 2 (4%)
		Gelechiidae	*Bryotropha terrella*	1 / 0 (1%)	0 / 0 (0%)	0 / 0 (0%)
			Dichomeris limosella	0 / 0 (0%)	0 / 0 (0%)	4 / 1 (7%)
			Gelechia turpella	2 / 0 (2%)	0 / 0 (0%)	0 / 0 (0%)
		Geometridae	*Abraxas grossulariata*	0 / 0 (0%)	1 / 0 (1%)	0 / 0 (0%)
			Agriopis leucophaearia	0 / 1 (1%)	0 / 0 (0%)	0 / 0 (0%)
			Agriopis marginaria	1 / 3 (3%)	0 / 0 (0%)	0 / 0 (0%)
			Alcis repandata	3 / 0 (3%)	1 / 0 (1%)	0 / 0 (0%)
			Aplocera plagiata	0 / 0 (0%)	0 / 0 (0%)	0 / 1 (1%)
			Apocheima hispidaria	1 / 1 (2%)	0 / 0 (0%)	0 / 0 (0%)
			Cabera exanthemata	0 / 0 (0%)	0 / 0 (0%)	0 / 2 (3%)
			Cabera pusaria	1 / 0 (1%)	0 / 0 (0%)	0 / 0 (0%)
			Campaea margaritata	0 / 2 (2%)	0 / 0 (0%)	1 / 0 (1%)
			Camptogramma bilineata	12 / 6 (15%)	0 / 2 (2%)	1 / 1 (3%)

Class	Order	Family	MOTU	*P. auritus* (n = 53/66)	*P. austriacus* (n = 22/66)	*P. macrobullaris* (n = 21/54)
			Charissa italohelveticus	0 / 0 (0%)	0 / 0 (0%)	1 / 0 (1%)
			Chiasmia clathrata	1 / 0 (1%)	0 / 2 (2%)	4 / 2 (8%)
			Chlorissa viridata	1 / 0 (1%)	0 / 0 (0%)	0 / 0 (0%)
			Chloroclysta siterata	0 / 0 (0%)	0 / 0 (0%)	1 / 0 (1%)
			Colotois pennaria	4 / 3 (6%)	0 / 0 (0%)	0 / 0 (0%)
			Crocallis elinguaria	1 / 0 (1%)	0 / 0 (0%)	0 / 0 (0%)
			Cyclophora linearia	0 / 0 (0%)	0 / 1 (1%)	0 / 0 (0%)
			Cyclophora punctaria	1 / 0 (1%)	0 / 0 (0%)	0 / 0 (0%)
			Deileptenia ribeata	1 / 0 (1%)	0 / 0 (0%)	0 / 0 (0%)
			Dysstroma truncata	1 / 0 (1%)	0 / 0 (0%)	0 / 0 (0%)
			Ectropis crepuscularia	1 / 0 (1%)	1 / 0 (1%)	0 / 0 (0%)
			Elophos dilucidaria	0 / 0 (0%)	0 / 0 (0%)	1 / 0 (1%)
			Ematurga atomaria	0 / 0 (0%)	0 / 1 (1%)	0 / 0 (0%)
			Ennomos quercinaria	1 / 2 (3%)	0 / 0 (0%)	0 / 0 (0%)
			Epirrhoe alternata	0 / 2 (2%)	0 / 0 (0%)	1 / 0 (1%)
			Epirrita christyi	1 / 0 (1%)	0 / 0 (0%)	0 / 0 (0%)
			Erannis defoliaria	1 / 1 (2%)	0 / 0 (0%)	0 / 0 (0%)
			Eupithecia abbreviata	1 / 1 (2%)	1 / 0 (1%)	0 / 0 (0%)
			Eupithecia dodoneata	1 / 0 (1%)	0 / 0 (0%)	0 / 0 (0%)
			Eupithecia haworthiata	1 / 0 (1%)	0 / 0 (0%)	0 / 0 (0%)
			Eupithecia tantillaria	3 / 0 (3%)	0 / 0 (0%)	0 / 0 (0%)
			Gnophos furvata	0 / 0 (0%)	0 / 0 (0%)	1 / 0 (1%)
			Hemistola chrysoprasaria	0 / 0 (0%)	0 / 0 (0%)	1 / 0 (1%)
			Hemithea aestivaria	0 / 4 (3%)	0 / 0 (0%)	0 / 0 (0%)
			Horisme radicaria	0 / 0 (0%)	0 / 0 (0%)	1 / 1 (3%)
			Hydriomena furcata	1 / 0 (1%)	0 / 0 (0%)	0 / 0 (0%)
			Hypomecis roboraria	0 / 0 (0%)	1 / 0 (1%)	0 / 0 (0%)
			Idaea aversata	4 / 6 (8%)	1 / 1 (2%)	0 / 0 (0%)
			Idaea dimidiata	0 / 0 (0%)	0 / 1 (1%)	0 / 0 (0%)
			Idaea fuscovenosa	1 / 0 (1%)	0 / 1 (1%)	0 / 0 (0%)
			Idaea humiliata	0 / 0 (0%)	1 / 0 (1%)	0 / 0 (0%)
			Idaea ochrata	0 / 0 (0%)	0 / 1 (1%)	0 / 0 (0%)
			Idaea rusticata	1 / 0 (1%)	0 / 0 (0%)	1 / 0 (1%)
			Idaea seriata	0 / 0 (0%)	0 / 0 (0%)	0 / 1 (1%)
			Idaea straminata	1 / 1 (2%)	0 / 1 (1%)	0 / 1 (1%)
			Jodis lactearia	1 / 0 (1%)	0 / 0 (0%)	0 / 0 (0%)
			Ligdia adustata	0 / 0 (0%)	0 / 0 (0%)	1 / 0 (1%)
			Lobophora halterata	0 / 0 (0%)	1 / 0 (1%)	0 / 0 (0%)
			Lomaspilis marginata	0 / 0 (0%)	1 / 0 (1%)	0 / 0 (0%)
			Lomographa temerata	1 / 0 (1%)	0 / 0 (0%)	0 / 0 (0%)
			Lycia hirtaria	1 / 1 (2%)	0 / 0 (0%)	0 / 0 (0%)
			Macaria liturata	1 / 0 (1%)	0 / 0 (0%)	0 / 0 (0%)
			Melanthia procellata	0 / 0 (0%)	1 / 0 (1%)	0 / 0 (0%)
			Menophra abruptaria	0 / 0 (0%)	0 / 0 (0%)	1 / 0 (1%)
			Nychiodes obscuraria	0 / 0 (0%)	0 / 0 (0%)	1 / 0 (1%)
			Odontopera bidentata	1 / 0 (1%)	0 / 0 (0%)	0 / 0 (0%)
			Operophtera brumata	4 / 8 (10%)	0 / 0 (0%)	0 / 0 (0%)
			Operophtera fagata	2 / 0 (2%)	0 / 0 (0%)	0 / 0 (0%)
			Opisthograptis luteolata	1 / 1 (2%)	0 / 2 (2%)	0 / 0 (0%)
			Pasiphila chloerata	0 / 1 (1%)	0 / 0 (0%)	0 / 0 (0%)
			Pasiphila rectangulata	1 / 1 (2%)	0 / 0 (0%)	0 / 0 (0%)
			Pelurga comitata	1 / 0 (1%)	1 / 1 (2%)	0 / 0 (0%)
			Peribatodes rhomboidaria	5 / 4 (8%)	3 / 0 (3%)	1 / 1 (3%)
			Peribatodes secundaria	1 / 0 (1%)	0 / 0 (0%)	0 / 0 (0%)
			Plagodis dolabraria	0 / 0 (0%)	0 / 1 (1%)	0 / 0 (0%)
			Rhodometra sacraria	0 / 1 (1%)	0 / 1 (1%)	2 / 0 (3%)
			Scopula immorata	0 / 0 (0%)	0 / 1 (1%)	0 / 1 (1%)
			Scopula immutata	1 / 2 (3%)	0 / 0 (0%)	2 / 3 (7%)
			Scopula virgulata	0 / 0 (0%)	0 / 0 (0%)	0 / 3 (4%)
			Selenia tetralunaria	0 / 1 (1%)	0 / 0 (0%)	0 / 0 (0%)
			Stegania trimaculata	0 / 0 (0%)	0 / 0 (0%)	1 / 1 (3%)
			Thalera fimbrialis	0 / 0 (0%)	1 / 1 (2%)	0 / 0 (0%)
			Timandra comae	2 / 1 (3%)	0 / 2 (2%)	1 / 0 (1%)
			Triphosa dubitata	0 / 0 (0%)	0 / 1 (1%)	0 / 0 (0%)

Class	Order	Family	MOTU	*P. auritus* (n = 53/66)	*P. austriacus* (n = 22/66)	*P. macrobullaris* (n = 21/54)
			Xanthorhoe ferrugata	1 / 1 (2%)	1 / 2 (3%)	1 / 1 (3%)
			Xanthorhoe fluctuata	0 / 0 (0%)	0 / 0 (0%)	0 / 1 (1%)
			Geometridae sp.	0 / 0 (0%)	0 / 0 (0%)	0 / 1 (1%)
		Hepialidae	*Korscheltellus lupulinus*	10 / 2 (10%)	1 / 3 (5%)	8 / 18 (35%)
			Triodia sylvina	12 / 13 (21%)	6 / 10 (18%)	6 / 11 (23%)
		Lasiocampidae	*Lasiocampa quercus*	1 / 1 (2%)	0 / 3 (3%)	0 / 0 (0%)
			Malacosoma neustria	1 / 4 (4%)	0 / 0 (0%)	1 / 1 (3%)
		Limacodidae	*Apoda limacodes*	0 / 6 (5%)	0 / 0 (0%)	0 / 0 (0%)
		Noctuidae	*Abrostola triplasia*	4 / 2 (5%)	4 / 3 (8%)	1 / 2 (4%)
			Acronicta psi	0 / 0 (0%)	0 / 2 (2%)	0 / 0 (0%)
			Acronicta rumicis	7 / 2 (8%)	1 / 4 (6%)	2 / 1 (4%)
			Actinotia polyodon	1 / 0 (1%)	0 / 1 (1%)	1 / 0 (1%)
			Agrochola circellaris	3 / 1 (3%)	0 / 0 (0%)	0 / 0 (0%)
			Agrochola litura	2 / 1 (3%)	0 / 0 (0%)	0 / 0 (0%)
			Agrochola lota	2 / 0 (2%)	1 / 0 (1%)	0 / 0 (0%)
			Agrochola lunosa	1 / 0 (1%)	0 / 0 (0%)	0 / 0 (0%)
			Agrochola lychnidis	1 / 1 (2%)	1 / 0 (1%)	0 / 0 (0%)
			Agrochola pistacinoides	4 / 1 (4%)	0 / 1 (1%)	1 / 4 (7%)
			Agrotis bigramma	0 / 0 (0%)	2 / 1 (3%)	1 / 0 (1%)
			Agrotis exclamationis	32 / 32 (54%)	15 / 44 (67%)	17 / 32 (65%)
			Agrotis ipsilon	27 / 18 (38%)	12 / 36 (55%)	14 / 15 (39%)
			Agrotis segetum	14 / 5 (16%)	10 / 14 (27%)	9 / 14 (31%)
			Allophyes oxyacanthae	2 / 1 (3%)	1 / 0 (1%)	0 / 0 (0%)
			Ammoconia caecimacula	0 / 0 (0%)	0 / 0 (0%)	3 / 4 (9%)
			Amphipoea oculea	0 / 0 (0%)	0 / 0 (0%)	1 / 0 (1%)
			Amphipyra berbera	5 / 11 (13%)	0 / 1 (1%)	1 / 0 (1%)
			Amphipyra pyramidea	15 / 16 (26%)	3 / 2 (6%)	1 / 0 (1%)
			Anaplectoides prasina	0 / 0 (0%)	0 / 1 (1%)	0 / 0 (0%)
			Anarta trifolii	2 / 1 (3%)	1 / 3 (5%)	1 / 3 (5%)
			Anorthoa munda	9 / 8 (14%)	0 / 0 (0%)	0 / 0 (0%)
			Apamea aquila	0 / 0 (0%)	1 / 0 (1%)	0 / 0 (0%)
			Apamea crenata	0 / 0 (0%)	0 / 1 (1%)	2 / 0 (3%)
			Apamea epomidion	1 / 0 (1%)	0 / 0 (0%)	0 / 0 (0%)
			Apamea lithoxylaea	2 / 1 (3%)	1 / 1 (2%)	0 / 0 (0%)
			Apamea monoglypha	8 / 3 (9%)	2 / 6 (9%)	2 / 0 (3%)
			Apamea scolopacina	1 / 0 (1%)	2 / 0 (2%)	0 / 0 (0%)
			Apamea sordens	2 / 1 (3%)	0 / 4 (5%)	0 / 0 (0%)
			Aporophyla lueneburgensis	2 / 0 (2%)	1 / 0 (1%)	2 / 4 (8%)
			Asteroscopus sphinx	1 / 2 (3%)	0 / 0 (0%)	0 / 0 (0%)
			Atethmia centrago	1 / 0 (1%)	0 / 0 (0%)	0 / 0 (0%)
			Athetis gluteosa	0 / 0 (0%)	0 / 0 (0%)	1 / 0 (1%)
			Autographa gamma	33 / 35 (57%)	15 / 35 (57%)	15 / 32 (63%)
			Autographa pulchrina	1 / 0 (1%)	0 / 0 (0%)	0 / 0 (0%)
			Axylia putris	7 / 8 (13%)	2 / 3 (6%)	5 / 7 (16%)
			Bryophila domestica	0 / 0 (0%)	0 / 0 (0%)	1 / 0 (1%)
			Calophasia lunula	0 / 0 (0%)	0 / 0 (0%)	1 / 0 (1%)
			Caradrina clavipalpis	1 / 0 (1%)	2 / 3 (6%)	0 / 0 (0%)
			Charanyca trigrammica	6 / 3 (8%)	1 / 1 (2%)	8 / 14 (29%)
			Conistra erythrocephala	0 / 2 (2%)	0 / 0 (0%)	0 / 0 (0%)
			Conistra rubiginea	1 / 0 (1%)	0 / 1 (1%)	1 / 0 (1%)
			Conistra rubiginosa	2 / 0 (2%)	0 / 0 (0%)	0 / 0 (0%)
			Conistra vaccinii	4 / 5 (8%)	0 / 1 (1%)	0 / 0 (0%)
			Cosmia affinis	3 / 1 (3%)	1 / 0 (1%)	0 / 0 (0%)
			Cosmia pyralina	0 / 1 (1%)	0 / 0 (0%)	0 / 1 (1%)
			Cosmia trapezina	27 / 18 (38%)	5 / 6 (13%)	5 / 1 (8%)
			Craniophora ligustri	2 / 1 (3%)	1 / 1 (2%)	1 / 1 (3%)
			Cryphia algae	7 / 0 (6%)	0 / 0 (0%)	0 / 0 (0%)
			Cucullia chamomillae	0 / 0 (0%)	0 / 0 (0%)	1 / 0 (1%)
			Cucullia umbratica	3 / 6 (8%)	4 / 2 (7%)	4 / 9 (17%)
			Diachrysia stenochrysis	1 / 0 (1%)	1 / 0 (1%)	0 / 2 (3%)
			Dichonia aprilina	5 / 3 (7%)	0 / 0 (0%)	0 / 0 (0%)
			Dicycla oo	0 / 1 (1%)	0 / 0 (0%)	2 / 0 (3%)
			Dryobotodes eremita	5 / 2 (6%)	0 / 0 (0%)	0 / 0 (0%)
			Egira conspicillaris	1 / 0 (1%)	0 / 0 (0%)	0 / 0 (0%)

Class	Order	Family	MOTU	*P. auritus* (n = 53/66)	*P. austriacus* (n = 22/66)	*P. macrobullaris* (n = 21/54)
			Elaphria venustula	0 / 0 (0%)	0 / 0 (0%)	1 / 0 (1%)
			Emmelia trabealis	0 / 0 (0%)	0 / 0 (0%)	1 / 2 (4%)
			Episema glaucina	0 / 0 (0%)	0 / 0 (0%)	1 / 0 (1%)
			Eugnorisma depuncta	2 / 2 (3%)	1 / 0 (1%)	1 / 0 (1%)
			Eugnorisma glareosa	2 / 2 (3%)	0 / 0 (0%)	2 / 2 (5%)
			Eupsilia transversa	1 / 1 (2%)	0 / 0 (0%)	0 / 0 (0%)
			Euxoa aquilina	0 / 0 (0%)	0 / 0 (0%)	2 / 0 (3%)
			Euxoa decora	0 / 0 (0%)	0 / 0 (0%)	1 / 0 (1%)
			Euxoa recussa	0 / 0 (0%)	0 / 0 (0%)	1 / 0 (1%)
			Hadena bicruris	0 / 0 (0%)	1 / 1 (2%)	0 / 1 (1%)
			Hadena compta	2 / 0 (2%)	0 / 0 (0%)	0 / 0 (0%)
			Hecatera bicolorata	1 / 0 (1%)	0 / 0 (0%)	0 / 0 (0%)
			Hecatera dysodea	1 / 1 (2%)	0 / 0 (0%)	0 / 0 (0%)
			Helicoverpa armigera	11 / 18 (24%)	9 / 11 (23%)	4 / 8 (16%)
			Heliothis peltigera	8 / 6 (12%)	3 / 15 (20%)	3 / 3 (8%)
			Hoplodrina ambigua	16 / 19 (29%)	9 / 25 (39%)	11 / 18 (39%)
			Hoplodrina blanda	9 / 0 (8%)	2 / 2 (5%)	3 / 1 (5%)
			Hoplodrina octogenaria	3 / 0 (3%)	1 / 1 (2%)	0 / 0 (0%)
			Lacanobia oleracea	0 / 1 (1%)	0 / 1 (1%)	0 / 0 (0%)
			Lacanobia suasa	0 / 1 (1%)	0 / 0 (0%)	0 / 0 (0%)
			Lithophane ornitopus	0 / 1 (1%)	1 / 0 (1%)	1 / 0 (1%)
			Lithophane semibrunnea	2 / 0 (2%)	0 / 0 (0%)	1 / 1 (3%)
			Lithophane socia	3 / 2 (4%)	0 / 1 (1%)	0 / 1 (1%)
			Luperina dumerilii	0 / 0 (0%)	0 / 0 (0%)	3 / 11 (19%)
			Macdunnoughia confusa	4 / 6 (8%)	2 / 3 (6%)	5 / 7 (16%)
			Mamestra brassicae	9 / 7 (13%)	5 / 8 (15%)	0 / 1 (1%)
			Melanchra persicariae	1 / 0 (1%)	1 / 0 (1%)	0 / 0 (0%)
			Mesapamea secalis/secalella/didyma	24 / 15 (33%)	8 / 12 (23%)	5 / 1 (8%)
			Mesoligia furuncula	7 / 1 (7%)	0 / 1 (1%)	0 / 0 (0%)
			Mniotype satura	1 / 0 (1%)	1 / 0 (1%)	0 / 0 (0%)
			Mormo maura	1 / 0 (1%)	1 / 0 (1%)	0 / 0 (0%)
			Mythimna albipuncta	39 / 31 (59%)	14 / 40 (61%)	11 / 23 (45%)
			Mythimna conigera	1 / 0 (1%)	0 / 0 (0%)	0 / 0 (0%)
			Mythimna ferrago	0 / 0 (0%)	0 / 0 (0%)	1 / 0 (1%)
			Mythimna impura	1 / 0 (1%)	0 / 0 (0%)	0 / 0 (0%)
			Mythimna l-album	2 / 0 (2%)	1 / 2 (3%)	0 / 0 (0%)
			Mythimna loreyi	1 / 0 (1%)	0 / 0 (0%)	0 / 0 (0%)
			Mythimna pallens	7 / 18 (21%)	4 / 15 (22%)	6 / 9 (20%)
			Mythimna unipuncta	0 / 2 (2%)	0 / 0 (0%)	0 / 0 (0%)
			Mythimna vitellina	6 / 5 (9%)	1 / 7 (9%)	2 / 4 (8%)
			Noctua comes	9 / 4 (11%)	1 / 7 (9%)	2 / 2 (5%)
			Noctua fimbriata	3 / 3 (5%)	0 / 0 (0%)	1 / 0 (1%)
			Noctua janthe	1 / 0 (1%)	0 / 1 (1%)	0 / 1 (1%)
			Noctua janthina	1 / 0 (1%)	1 / 0 (1%)	0 / 0 (0%)
			Noctua orbona	0 / 0 (0%)	0 / 1 (1%)	0 / 0 (0%)
			Noctua pronuba	21 / 26 (39%)	12 / 35 (53%)	7 / 15 (29%)
			Ochropleura plecta	10 / 7 (14%)	3 / 7 (11%)	3 / 5 (11%)
			Oligia latruncula	8 / 4 (10%)	1 / 1 (2%)	3 / 6 (12%)
			Oligia strigilis	1 / 2 (3%)	2 / 1 (3%)	1 / 0 (1%)
			Oligia versicolor	2 / 0 (2%)	0 / 1 (1%)	2 / 0 (3%)
			Orthosia cerasi	1 / 2 (3%)	1 / 0 (1%)	0 / 0 (0%)
			Orthosia gothica	1 / 0 (1%)	0 / 0 (0%)	0 / 0 (0%)
			Orthosia gracilis/opima	0 / 1 (1%)	0 / 0 (0%)	0 / 0 (0%)
			Orthosia incerta	0 / 0 (0%)	0 / 0 (0%)	1 / 0 (1%)
			Parastichtis ypsillon	2 / 3 (4%)	0 / 0 (0%)	0 / 0 (0%)
			Peridroma saucia	7 / 5 (10%)	2 / 4 (7%)	3 / 6 (12%)
			Phlogophora meticulosa	11 / 8 (16%)	4 / 10 (16%)	1 / 0 (1%)
			Polia nebulosa	1 / 1 (2%)	0 / 0 (0%)	0 / 0 (0%)
			Polyphaenis sericata	2 / 0 (2%)	0 / 0 (0%)	0 / 0 (0%)
			Protodeltote pygarga	0 / 0 (0%)	0 / 1 (1%)	0 / 0 (0%)
			Pyrrhia umbra	1 / 1 (2%)	0 / 0 (0%)	3 / 1 (5%)
			Rusina ferruginea	0 / 0 (0%)	0 / 0 (0%)	0 / 1 (1%)
			Sideridis reticulata	0 / 0 (0%)	0 / 0 (0%)	0 / 1 (1%)
			Spodoptera exigua	7 / 7 (12%)	3 / 2 (6%)	2 / 3 (7%)

Class	Order	Family	MOTU	*P. auritus* (n = 53/66)	*P. austriacus* (n = 22/66)	*P. macrobullaris* (n = 21/54)
			Thalpophila matura	8 / 3 (9%)	2 / 3 (6%)	4 / 8 (16%)
			Tholera cespitis	0 / 0 (0%)	0 / 0 (0%)	3 / 3 (8%)
			Tholera decimalis	1 / 1 (2%)	0 / 0 (0%)	4 / 12 (21%)
			Tiliacea aurago	3 / 3 (5%)	0 / 0 (0%)	0 / 0 (0%)
			Tiliacea citrago	5 / 6 (9%)	0 / 0 (0%)	0 / 0 (0%)
			Trachea atriplicis	11 / 4 (13%)	3 / 7 (11%)	4 / 5 (12%)
			Trichoplusia ni	3 / 1 (3%)	4 / 3 (8%)	1 / 1 (3%)
			Xanthia gilvago	3 / 1 (3%)	1 / 0 (1%)	0 / 0 (0%)
			Xanthia icteritia	1 / 2 (3%)	0 / 0 (0%)	0 / 0 (0%)
			Xanthia ocellaris	1 / 0 (1%)	1 / 0 (1%)	0 / 0 (0%)
			Xestia baja	1 / 0 (1%)	0 / 0 (0%)	0 / 0 (0%)
			Xestia castanea	0 / 0 (0%)	0 / 0 (0%)	1 / 2 (4%)
			Xestia c-nigrum	22 / 10 (27%)	7 / 17 (27%)	5 / 16 (28%)
			Xestia rhomboidea	2 / 0 (2%)	0 / 0 (0%)	0 / 0 (0%)
			Xestia sexstrigata	0 / 0 (0%)	0 / 0 (0%)	1 / 0 (1%)
			Xestia xanthographa	10 / 9 (16%)	1 / 11 (14%)	6 / 15 (28%)
			Xylena sp.	1 / 0 (1%)	0 / 0 (0%)	0 / 0 (0%)
			Noctuidae sp. 1	0 / 0 (0%)	0 / 0 (0%)	1 / 0 (1%)
			Noctuidae sp. 2	1 / 0 (1%)	0 / 0 (0%)	0 / 0 (0%)
			Noctuidae sp. 3	1 / 0 (1%)	0 / 0 (0%)	0 / 0 (0%)
			Noctuidae sp. 4	0 / 0 (0%)	0 / 0 (0%)	0 / 1 (1%)
			Noctuidae sp. 5	0 / 0 (0%)	0 / 0 (0%)	0 / 1 (1%)
			Noctuidae sp. 6	0 / 0 (0%)	0 / 0 (0%)	0 / 1 (1%)
			Noctuidae sp. 7	0 / 2 (2%)	1 / 0 (1%)	0 / 0 (0%)
		Nolidae	*Bena bicolorana*	1 / 0 (1%)	1 / 1 (2%)	0 / 0 (0%)
			Meganola strigula	0 / 1 (1%)	0 / 0 (0%)	0 / 0 (0%)
			Pseudoips prasinana	5 / 9 (12%)	1 / 0 (1%)	0 / 0 (0%)
		Notodontidae	*Drymonia dodonaea*	0 / 1 (1%)	0 / 0 (0%)	1 / 0 (1%)
			Drymonia ruficornis	2 / 5 (6%)	1 / 1 (2%)	0 / 0 (0%)
			Furcula furcula	0 / 0 (0%)	0 / 1 (1%)	0 / 0 (0%)
			Harpyia milhauseri	1 / 0 (1%)	0 / 0 (0%)	0 / 0 (0%)
			Phalera bucephala	0 / 1 (1%)	1 / 0 (1%)	0 / 0 (0%)
			Pheosia tremula	2 / 0 (2%)	0 / 0 (0%)	0 / 0 (0%)
			Pterostoma palpina	0 / 0 (0%)	1 / 0 (1%)	1 / 0 (1%)
			Ptilodon capucina	0 / 0 (0%)	0 / 0 (0%)	0 / 1 (1%)
			Stauropus fagi	0 / 1 (1%)	0 / 0 (0%)	0 / 0 (0%)
			Thaumetopoea pityocampa	0 / 0 (0%)	0 / 1 (1%)	0 / 0 (0%)
			Thaumetopoea processionea	1 / 1 (2%)	1 / 3 (5%)	0 / 0 (0%)
		Oecophoridae	*Batia lunaris*	0 / 0 (0%)	0 / 0 (0%)	1 / 1 (3%)
			Epicallima formosella	0 / 0 (0%)	0 / 0 (0%)	1 / 2 (4%)
			Hofmannophila pseudospretella	1 / 0 (1%)	0 / 0 (0%)	0 / 0 (0%)
			Metalampra italica	0 / 0 (0%)	0 / 0 (0%)	0 / 2 (3%)
		Praydidae	*Prays fraxinella*	1 / 0 (1%)	0 / 0 (0%)	0 / 0 (0%)
		Pterophoridae	*Emmelina monodactyla*	1 / 0 (1%)	0 / 0 (0%)	0 / 0 (0%)
		Pyralidae	*Acrobasis advenella*	2 / 0 (2%)	0 / 0 (0%)	0 / 0 (0%)
			Acrobasis repandana	1 / 0 (1%)	0 / 0 (0%)	0 / 0 (0%)
			Acrobasis tumidana	0 / 1 (1%)	0 / 0 (0%)	0 / 0 (0%)
			Aglossa pinguinalis	0 / 0 (0%)	0 / 1 (1%)	0 / 0 (0%)
			Agriphila geniculea	0 / 1 (1%)	2 / 0 (2%)	1 / 1 (3%)
			Agriphila inquinatella	1 / 0 (1%)	0 / 0 (0%)	1 / 2 (4%)
			Agriphila selasella	1 / 1 (2%)	1 / 0 (1%)	0 / 2 (3%)
			Agriphila straminella	1 / 0 (1%)	0 / 2 (2%)	0 / 0 (0%)
			Agriphila tristella	1 / 2 (3%)	1 / 0 (1%)	4 / 9 (17%)
			Anania coronata	1 / 0 (1%)	0 / 0 (0%)	0 / 0 (0%)
			Anania hortulata	0 / 0 (0%)	0 / 0 (0%)	0 / 1 (1%)
			Aphomia sociella	3 / 1 (3%)	1 / 4 (6%)	0 / 0 (0%)
			Calamotropha paludella	1 / 0 (1%)	0 / 0 (0%)	0 / 0 (0%)
			Catoptria falsella	0 / 0 (0%)	0 / 1 (1%)	0 / 0 (0%)
			Catoptria mytilella	0 / 0 (0%)	0 / 0 (0%)	1 / 0 (1%)
			Catoptria permutatellus	0 / 0 (0%)	0 / 0 (0%)	1 / 0 (1%)
			Catoptria verellus	1 / 0 (1%)	0 / 0 (0%)	0 / 0 (0%)
			Chrysoteuchia culmella	0 / 0 (0%)	1 / 5 (7%)	5 / 4 (12%)
			Crambus perlella	1 / 4 (4%)	0 / 0 (0%)	3 / 4 (9%)
			Crambus pratella	0 / 0 (0%)	0 / 0 (0%)	1 / 0 (1%)

Class	Order	Family	MOTU	*P. auritus* (n = 53/66)	*P. austriacus* (n = 22/66)	*P. macrobullaris* (n = 21/54)
			Cydalima perspectalis	6 / 2 (7%)	2 / 3 (6%)	1 / 2 (4%)
			Dioryctria abietella	2 / 1 (3%)	0 / 1 (1%)	0 / 0 (0%)
			Duponchelia fovealis	0 / 0 (0%)	0 / 0 (0%)	1 / 0 (1%)
			Endotricha flammealis	1 / 5 (5%)	0 / 0 (0%)	0 / 0 (0%)
			Eudonia delunella	2 / 2 (3%)	0 / 0 (0%)	0 / 0 (0%)
			Eudonia lacustrata	0 / 1 (1%)	0 / 0 (0%)	0 / 0 (0%)
			Eudonia mercurella	0 / 2 (2%)	0 / 0 (0%)	0 / 0 (0%)
			Galleria mellonella	2 / 2 (3%)	1 / 2 (3%)	0 / 0 (0%)
			Homoeosoma sinuella	0 / 0 (0%)	0 / 0 (0%)	1 / 2 (4%)
			Hypsopygia costalis	0 / 1 (1%)	0 / 0 (0%)	0 / 0 (0%)
			Nephopterix angustella	1 / 0 (1%)	0 / 1 (1%)	0 / 0 (0%)
			Nomophila noctuella	18 / 16 (29%)	11 / 21 (36%)	7 / 10 (23%)
			Oncocera semirubella	8 / 7 (13%)	0 / 3 (3%)	7 / 17 (32%)
			Ostrinia nubilalis	1 / 0 (1%)	1 / 0 (1%)	0 / 0 (0%)
			Paracorsia repandalis	0 / 0 (0%)	1 / 0 (1%)	0 / 0 (0%)
			Pediasia contaminella	3 / 0 (3%)	0 / 0 (0%)	0 / 4 (5%)
			Phycita roborella	4 / 3 (6%)	1 / 1 (2%)	2 / 1 (4%)
			Phycitodes inquinatella	0 / 0 (0%)	0 / 0 (0%)	0 / 1 (1%)
			Pleuroptya ruralis	1 / 0 (1%)	1 / 2 (3%)	0 / 0 (0%)
			Pyralis farinalis	1 / 0 (1%)	0 / 0 (0%)	0 / 0 (0%)
			Pyrausta despicata	1 / 1 (2%)	1 / 0 (1%)	4 / 6 (13%)
			Pyrausta purpuralis	1 / 0 (1%)	1 / 2 (3%)	5 / 2 (9%)
			Sciota rhenella	0 / 0 (0%)	0 / 0 (0%)	0 / 1 (1%)
			Scoparia ambigualis/basistrigalis	1 / 0 (1%)	0 / 0 (0%)	0 / 0 (0%)
			Scoparia conicella	0 / 0 (0%)	1 / 0 (1%)	0 / 0 (0%)
			Scoparia pyralella	1 / 0 (1%)	0 / 0 (0%)	0 / 0 (0%)
			Selagia spadicella	0 / 0 (0%)	0 / 0 (0%)	0 / 5 (7%)
			Sitochroa palealis	1 / 0 (1%)	0 / 0 (0%)	0 / 0 (0%)
			Sitochroa verticalis	2 / 0 (2%)	0 / 1 (1%)	0 / 4 (5%)
			Synaphe punctalis	1 / 0 (1%)	0 / 0 (0%)	2 / 0 (3%)
			Udea ferrugalis	0 / 0 (0%)	0 / 1 (1%)	0 / 0 (0%)
			Pyralidae sp. 1	0 / 1 (1%)	0 / 0 (0%)	0 / 0 (0%)
			Pyralidae sp. 2	0 / 0 (0%)	0 / 0 (0%)	0 / 1 (1%)
			Pyralidae sp. 3	0 / 0 (0%)	0 / 0 (0%)	0 / 1 (1%)
			Pyralidae sp. 4	0 / 0 (0%)	0 / 0 (0%)	0 / 2 (3%)
		Sphingidae	*Deilephila porcellus*	0 / 3 (3%)	0 / 0 (0%)	0 / 0 (0%)
			Hyloicus pinastri	0 / 0 (0%)	0 / 1 (1%)	0 / 0 (0%)
			Laothoe populi	2 / 0 (2%)	0 / 2 (2%)	0 / 0 (0%)
			Macroglossum stellatarum	2 / 0 (2%)	0 / 0 (0%)	0 / 0 (0%)
			Mimas tiliae	1 / 2 (3%)	0 / 0 (0%)	0 / 0 (0%)
		Tineidae	*Montescardia tessulatellus*	0 / 0 (0%)	0 / 2 (2%)	1 / 1 (3%)
			Nemapogon granella	0 / 0 (0%)	0 / 0 (0%)	1 / 0 (1%)
		Tortricidae	*Acleris cristana*	0 / 1 (1%)	0 / 0 (0%)	0 / 0 (0%)
			Acleris hastiana	0 / 0 (0%)	0 / 0 (0%)	1 / 0 (1%)
			Ancylis achatana	0 / 0 (0%)	0 / 0 (0%)	1 / 0 (1%)
			Archips crataegana	1 / 4 (4%)	0 / 0 (0%)	0 / 0 (0%)
			Archips oporana	0 / 0 (0%)	0 / 0 (0%)	0 / 2 (3%)
			Archips rosana	0 / 0 (0%)	0 / 0 (0%)	0 / 2 (3%)
			Archips xylosteana	1 / 10 (9%)	0 / 0 (0%)	1 / 0 (1%)
			Celypha rivulana	0 / 0 (0%)	0 / 0 (0%)	1 / 2 (4%)
			Celypha striana	1 / 0 (1%)	3 / 6 (10%)	3 / 4 (9%)
			Cnephasia asseclana	1 / 0 (1%)	0 / 0 (0%)	0 / 0 (0%)
			Cnephasia communana	1 / 0 (1%)	0 / 0 (0%)	0 / 0 (0%)
			Cnephasia cupressivorana	1 / 2 (3%)	0 / 0 (0%)	0 / 0 (0%)
			Cnephasia incertana	2 / 1 (3%)	0 / 0 (0%)	0 / 1 (1%)
			Cnephasia pasiuana	0 / 0 (0%)	0 / 1 (1%)	0 / 0 (0%)
			Cnephasia stephensiana	3 / 2 (4%)	0 / 1 (1%)	3 / 2 (7%)
			Cydia fagiglandana	1 / 0 (1%)	0 / 0 (0%)	0 / 0 (0%)
			Cydia pomonella	4 / 4 (7%)	0 / 0 (0%)	0 / 0 (0%)
			Cydia splendana	1 / 1 (2%)	0 / 0 (0%)	0 / 0 (0%)
			Epinotia tenerana	1 / 0 (1%)	0 / 0 (0%)	0 / 0 (0%)
			Eudemis porphyrana	1 / 0 (1%)	0 / 0 (0%)	0 / 0 (0%)
			Eudemis profundana	2 / 2 (3%)	0 / 0 (0%)	0 / 0 (0%)
			Gypsonoma dealbana	0 / 2 (2%)	0 / 0 (0%)	0 / 1 (1%)

Class	Order	Family	MOTU	*P. auritus* (n = 53/66)	*P. austriacus* (n = 22/66)	*P. macrobullaris* (n = 21/54)
			Hedya nubiferana	0 / 0 (0%)	0 / 1 (1%)	0 / 0 (0%)
			Pandemis cerasana	1 / 6 (6%)	0 / 0 (0%)	1 / 0 (1%)
			Rhyacionia buoliana	0 / 0 (0%)	0 / 0 (0%)	0 / 1 (1%)
			Tortrix viridana	3 / 4 (6%)	0 / 0 (0%)	1 / 2 (4%)
			Zeiraphera griseana	1 / 0 (1%)	0 / 0 (0%)	0 / 0 (0%)
			Zeiraphera isertana	2 / 9 (9%)	1 / 2 (3%)	0 / 0 (0%)
		Yponomeutidae	*Yponomeuta* sp.	1 / 2 (3%)	0 / 0 (0%)	0 / 0 (0%)
		Ypsolophidae	*Ypsolopha parenthesella*	1 / 0 (1%)	0 / 0 (0%)	0 / 0 (0%)
			Ypsolopha ustella	0 / 0 (0%)	0 / 0 (0%)	1 / 1 (3%)
		Unknown	Lepidoptera sp. 1	5 / 2 (6%)	0 / 0 (0%)	2 / 1 (4%)
			Lepidoptera sp. 2	3 / 3 (5%)	0 / 0 (0%)	0 / 0 (0%)
			Lepidoptera sp. 3	0 / 0 (0%)	0 / 0 (0%)	1 / 0 (1%)
			Lepidoptera sp. 4	0 / 0 (0%)	0 / 0 (0%)	0 / 2 (3%)
			Lepidoptera sp. 5	0 / 0 (0%)	0 / 0 (0%)	0 / 1 (1%)
			Lepidoptera sp. 6	0 / 0 (0%)	1 / 0 (1%)	0 / 0 (0%)
			Lepidoptera sp. 7	0 / 0 (0%)	0 / 1 (1%)	0 / 0 (0%)
			Lepidoptera sp. 8	0 / 1 (1%)	0 / 0 (0%)	0 / 1 (1%)
			Lepidoptera sp. 9	0 / 0 (0%)	0 / 1 (1%)	0 / 0 (0%)
			Lepidoptera sp. 10	1 / 2 (3%)	0 / 0 (0%)	0 / 0 (0%)
			Lepidoptera sp. 11	0 / 0 (0%)	1 / 0 (1%)	0 / 0 (0%)
			Lepidoptera sp. 12	0 / 0 (0%)	0 / 0 (0%)	1 / 0 (1%)
	Mecoptera	Panorpidae	*Panorpa germanica*	0 / 1 (1%)	0 / 0 (0%)	0 / 0 (0%)
	Neuroptera	Chrysopidae	*Chrysoperla carnea*	19 / 20 (33%)	4 / 14 (20%)	0 / 0 (0%)
			Chrysotropia ciliata	1 / 1 (2%)	0 / 0 (0%)	0 / 0 (0%)
			Nineta flava	2 / 3 (4%)	0 / 1 (1%)	0 / 0 (0%)
			Nothochrysa capitata	1 / 0 (1%)	0 / 0 (0%)	0 / 0 (0%)
			Nothochrysa fulviceps	2 / 0 (2%)	0 / 0 (0%)	0 / 0 (0%)
			Peyerimhoffina gracilis	2 / 1 (3%)	0 / 0 (0%)	0 / 0 (0%)
			Pseudomallada flavifrons	1 / 1 (2%)	0 / 0 (0%)	0 / 0 (0%)
			Pseudomallada prasinus	0 / 0 (0%)	0 / 0 (0%)	1 / 0 (1%)
			Chrysopidae sp.	0 / 2 (2%)	0 / 1 (1%)	0 / 0 (0%)
		Hemerobiidae	*Drepanepteryx phalaenoides*	1 / 0 (1%)	0 / 0 (0%)	0 / 0 (0%)
			Hemerobius humulinus	5 / 8 (11%)	0 / 2 (2%)	0 / 2 (3%)
			Hemerobius micans	1 / 3 (3%)	0 / 1 (1%)	0 / 0 (0%)
			Hemerobius pini	2 / 0 (2%)	0 / 0 (0%)	0 / 0 (0%)
		Myrmeleontidae	*Distoleon tetragrammicus*	0 / 0 (0%)	0 / 1 (1%)	0 / 0 (0%)
	Orthoptera	Acrididae	*Chorthippus parallelus*	1 / 0 (1%)	0 / 0 (0%)	0 / 0 (0%)
		Tettigoniidae	*Leptophyes punctatissima*	2 / 0 (2%)	0 / 0 (0%)	0 / 0 (0%)
			Meconema thalassinum	5 / 2 (6%)	0 / 0 (0%)	0 / 0 (0%)
	Psocodea	Caeciliusidae	*Caecilius fuscopterus*	0 / 1 (1%)	0 / 0 (0%)	0 / 0 (0%)
			Valenzuela flavidus	0 / 1 (1%)	0 / 0 (0%)	0 / 0 (0%)
		Psocidae	*Loensia pearmani*	0 / 0 (0%)	1 / 0 (1%)	0 / 0 (0%)
			Metylophorus nebulosus	0 / 2 (2%)	0 / 0 (0%)	0 / 0 (0%)
			Psococerastis gibbosa	0 / 4 (3%)	0 / 0 (0%)	0 / 0 (0%)
	Raphidioptera	Raphidiidae	*Subilla confinis*	0 / 1 (1%)	0 / 0 (0%)	0 / 0 (0%)
	Trichoptera	Hydropsychidae	*Hydropsyche instabilis*	0 / 0 (0%)	0 / 0 (0%)	1 / 0 (1%)
			Hydropsyche pellucidula	0 / 0 (0%)	0 / 0 (0%)	0 / 1 (1%)
		Leptoceridae	*Ceraclea annulicornis*	0 / 0 (0%)	0 / 1 (1%)	0 / 0 (0%)
		Limnephilidae	*Stenophylax mitis*	1 / 0 (1%)	0 / 0 (0%)	0 / 0 (0%)
		Polycentropodidae	*Plectrocnemia conspersa*	1 / 0 (1%)	0 / 0 (0%)	1 / 0 (1%)
		Rhyacophilidae	*Rhyacophila dorsalis*	0 / 1 (1%)	0 / 0 (0%)	0 / 0 (0%)
Malacostraca	Isopoda	Oniscidae	*Oniscus asellus*	0 / 1 (1%)	0 / 0 (0%)	0 / 0 (0%)
		Porcellionidae	*Porcellio scaber*	1 / 0 (1%)	0 / 0 (0%)	0 / 0 (0%)
			Porcellio spinicornis	1 / 0 (1%)	0 / 0 (0%)	0 / 0 (0%)
		Unknown	Isopoda sp.	0 / 1 (1%)	0 / 0 (0%)	0 / 0 (0%)

Appendix 2. Prey traits table

Matrix summarizing ecological traits for all the 687 MOTUs identified in the feces of *Plecotus* bats.

MOTU	Pest (1) vector (2)	Parasitoid (1)	*Unknown*	Closed habitats	Woodlands	Semi-open habitats	Hedgerows	Shrublands	Open habitats	Cultivated lands	Meadows	Lawns	Mountainous areas	Screes	Slopes	Urban areas	Lowlands	Mesophilous areas	Ubiquitous	Dry areas	Diurnal or flightless	Largeness (size)	**References**
Coelotes terrestris	0	0	0	1	1	0	0	0	0	0	0	0	0	0	0	0	0	0	0	0	1	2	[1]
Amaurobius fenestralis	0	0	0	1	1	1	0	1	1	1	0	0	0	0	0	0	0	0	0	0	1	3	[1]
Anyphaena accentuata	0	0	0	1	1	1	0	1	0	0	0	0	0	0	0	0	0	0	0	0	1	2	[1]
Gibbaranea gibbosa	0	0	0	1	1	1	1	0	1	0	0	0	0	0	0	0	0	0	0	0	1	3	[1]
Nuctenea umbratica	0	0	0	1	1	1	0	0	1	0	0	0	0	0	0	1	0	0	0	0	1	3	[1]
Clubiona corticalis	0	0	0	1	1	1	0	0	0	0	0	0	0	0	0	1	0	0	0	0	1	2	[1]
Clubiona pallidula	0	0	0	1	1	1	1	0	0	0	0	0	0	0	0	0	0	0	0	0	1	2	[1]
Cheiracanthium mildei	0	0	0	1	1	0	0	1	1	0	0	0	0	0	0	0	0	0	0	0	1	2	[2]
Philodromus aureolus	0	0	0	1	1	1	1	1	1	0	0	0	0	0	0	0	0	0	0	0	1	1	[1]
Philodromus buxi	0	0	0	0	0	1	0	1	0	0	0	0	0	0	0	0	0	0	0	0	1	1	[1]
Philodromus margaritatus	0	0	0	1	1	0	0	0	0	0	0	0	0	0	0	0	0	0	0	0	1	1	[1]
Salticus zebraneus	0	0	0	1	1	0	0	0	1	0	0	0	0	0	0	0	0	0	0	0	1	1	[1]
Diaea dorsata	0	0	0	1	1	0	0	0	0	0	0	0	0	0	0	0	0	0	0	0	1	2	[1]
Xysticus lanio	1	0	0	1	1	0	0	0	0	0	0	0	0	0	0	0	0	0	0	0	1	2	[1]
Sminthurus viridis	0	0	0	0	0	0	0	0	1	1	1	0	0	0	0	0	0	0	0	1	1	1	[3]
Ectobius sylvestris	0	0	0	1	1	0	0	0	0	0	0	0	0	0	0	0	0	0	0	0	1	2	[4]
Ectobius vittiventris	0	0	0	1	1	0	0	0	0	0	0	0	0	0	0	0	0	0	0	0	1	1	[4]
Amara similata	0	0	0	0	0	0	0	0	1	1	0	0	0	0	0	0	0	0	0	0	1	2	[5]
Bembidion quadrimaculatum	1	0	0	0	0	0	0	0	0	0	0	0	0	0	0	0	0	0	0	0	1	2	[6]
Harpalus rufipes	0	0	0	0	0	1	0	0	0	0	0	0	0	0	0	1	0	0	1	0	1	2	[5]
Acanthocinus griseus	0	0	0	1	1	0	0	0	0	0	0	0	0	0	0	0	0	0	0	0	0	2	[7]
Mesosa nebulosa	0	0	0	1	1	0	0	0	0	0	0	0	0	0	0	0	0	0	0	0	0	2	[7]
Opilo mollis	0	0	0	1	1	0	0	0	0	0	0	0	0	0	0	0	0	0	0	0	0	2	[8]
Curculio glandium	0	0	0	1	1	0	0	0	0	0	0	0	0	0	0	0	0	0	0	0	0	1	[9]
Dorytomus longimanus	0	0	0	0	0	1	0	0	0	0	0	0	0	0	0	0	0	0	0	0	0	1	[9]
Athous sp.	0	0	1	0	0	0	0	0	0	0	0	0	0	0	0	0	0	0	0	0	0	2	n.a.
Stenagostus rhombeus	0	0	0	1	1	0	0	0	0	0	0	0	0	0	0	0	0	0	0	0	0	2	[10]
Dasytes aeratus	1	0	0	1	1	0	0	0	0	0	0	0	0	0	0	0	0	0	0	0	0	1	[11, 12]
Amphimallon majale	1	0	0	0	0	0	0	0	1	0	0	0	0	0	0	0	0	0	0	0	0	2	[13, 14]
Rhizotrogus aestivus	1	0	0	0	0	0	0	0	1	0	0	0	0	0	0	0	0	0	0	0	0	2	[15]
Serica brunnea	0	0	0	0	0	0	0	0	1	0	0	0	0	0	0	0	0	0	0	0	0	2	[15 , 16]
Eusphalerum signatum	0	0	0	1	1	0	0	0	0	0	0	0	0	0	0	0	0	0	0	0	0	1	[17]
Coleoptera sp.	0	0	1	0	0	0	0	0	0	0	0	0	0	0	0	0	0	0	0	0	0	2	n.a.
Chelidurella guentheri	1	0	0	1	1	0	0	0	0	0	0	0	0	0	0	0	0	0	0	0	1	3	[18]
Forficula auricularia	0	0	0	1	1	1	0	0	1	1	0	0	0	0	0	0	0	0	0	0	1	3	[18]
Sylvicola cinctus	0	0	0	1	1	0	0	0	0	0	0	0	0	0	0	0	0	0	0	0	0	1	[19]
Sylvicola stackelbergi	0	0	0	1	1	0	0	0	0	0	0	0	0	0	0	0	0	0	0	0	0	1	[19]
Anthomyia sp.	1	0	1	0	0	0	0	0	0	0	0	0	0	0	0	0	0	0	0	0	0	1	n.a.
Botanophila fugax	1	0	0	0	0	0	0	0	1	0	1	0	0	0	0	0	0	0	0	0	0	1	[20]
Delia florilega	1	0	0	0	0	0	0	0	1	1	0	0	0	0	0	0	0	0	0	0	0	1	[20]
Delia platura	1	0	0	1	1	0	0	0	1	1	1	0	0	0	0	0	0	0	0	0	0	1	[21]
Delia radicum	0	0	0	0	0	0	0	0	1	1	1	0	0	0	0	0	0	0	0	0	0	1	[21]
Hydrophoria silvicola	0	0	0	1	1	0	0	0	0	0	0	0	0	0	0	0	0	0	0	0	0	1	[22]
Pegoplata infirma	1	0	0	0	0	1	0	0	1	1	0	0	0	0	0	0	0	0	0	0	0	1	[23]
Dilophus febrilis	0	0	0	0	0	1	0	0	0	0	1	0	0	0	0	0	0	0	0	0	0	1	[24]
Bellardia viarum	0	0	0	0	0	0	0	0	1	1	1	0	0	0	0	0	0	0	0	0	0	1	[25, 26]
Bellardia vulgaris	2	0	0	0	0	0	0	0	1	1	1	0	0	0	0	0	0	0	0	0	0	1	[25, 26]
Calliphora vicina	2	0	0	0	0	0	0	0	1	1	0	0	0	0	0	1	0	0	0	0	0	2	[25, 26]
Calliphora vomitoria	0	0	0	1	1	0	0	0	1	1	0	0	0	0	0	1	0	0	0	0	0	2	[25, 26]
Lucilia caesar	2	0	0	1	1	0	0	0	0	0	0	0	0	0	0	1	0	0	0	0	0	1	[25, 26]
Lucilia sericata	0	0	0	0	0	0	0	0	1	1	0	0	0	0	0	1	0	0	0	0	0	1	[25, 26]

MOTU	Pest (1) vector (2)	Parasitoid (1)	*Unknown*	Closed habitats	Woodlands	Semi-open habitats	Hedgerows	Shrublands	Open habitats	Cultivated lands	Meadows	Lawns	Mountainous areas	Screes	Slopes	Urban areas	Lowlands	Mesophilous areas	Ubiquitous	Dry areas	Diurnal or flightless	Largeness (size)	**References**
Pollenia hungarica	0	0	0	0	0	0	0	0	0	0	0	0	0	0	0	1	0	0	1	0	0	2	[25]
Pollenia pediculata	0	0	0	0	0	0	0	0	0	0	0	0	0	0	0	1	0	0	1	0	0	2	[25, 26]
Pollenia rudis	0	0	0	0	0	0	0	0	0	0	0	0	0	0	0	1	0	0	1	0	0	2	[25, 26]
Pollenia vagabunda	0	0	0	0	0	0	0	0	0	0	0	0	0	0	0	0	0	0	1	0	0	2	[25, 26]
Calliphoridae sp.	0	0	1	0	0	0	0	0	0	0	0	0	0	0	0	0	0	0	0	0	0	2	n.a.
Meoneura neottiophila	0	0	1	0	0	0	0	0	0	0	0	0	0	0	0	0	0	0	0	0	0	1	n.a.
Asteromyia sp.	0	0	1	0	0	0	0	0	0	0	0	0	0	0	0	0	0	0	0	0	0	1	n.a.
Mayetiola destructor	1	0	0	0	0	0	0	0	1	1	0	0	0	0	0	0	0	0	0	0	0	1	
Cecidomyiidae sp. 1	0	0	1	0	0	0	0	0	0	0	0	0	0	0	0	0	0	0	0	0	0	1	n.a.
Cecidomyiidae sp. 2	0	0	1	0	0	0	0	0	0	0	0	0	0	0	0	0	0	0	0	0	0	1	n.a.
Cecidomyiidae sp. 3	1	0	1	0	0	0	0	0	0	0	0	0	0	0	0	0	0	0	0	0	0	1	n.a.
Culicoides chiopterus	0	0	0	0	0	0	0	0	1	0	0	0	0	0	0	0	0	0	0	0	0	1	[27]
Conchapelopia melanops	0	0	0	0	0	0	0	0	1	0	0	0	0	0	0	0	0	0	0	0	0	1	[28]
Orthocladius rubicundus	0	0	0	0	0	0	0	0	1	0	0	0	0	0	0	0	0	0	0	0	0	1	[29]
Paratrichocladius rufiventris	0	0	0	0	0	0	0	0	1	0	0	0	0	0	0	0	0	0	0	0	0	1	[30]
Polypedilum convictum	0	0	0	0	0	0	0	0	1	0	0	0	0	0	0	0	0	0	0	0	0	1	[30]
Polypedilum sp.	0	0	0	0	0	0	0	0	1	0	0	0	0	0	0	0	0	0	0	0	0	1	[30]
Chironomidae sp. 1	0	0	1	0	0	0	0	0	0	0	0	0	0	0	0	0	0	0	0	0	0	1	n.a.
Chironomidae sp. 2	0	0	1	0	0	0	0	0	0	0	0	0	0	0	0	0	0	0	0	0	0	1	n.a.
Thaumatomyia notata	0	0	0	0	0	0	0	0	1	1	1	0	0	0	0	0	0	0	0	0	0	1	[31]
Culex pipiens	0	0	0	1	1	0	0	0	0	0	0	0	0	0	0	1	0	0	0	0	0	1	
Drosophila melanogaster	0	0	0	0	0	1	0	0	1	1	0	0	0	0	0	0	0	0	0	0	0	1	[31]
Drosophila subobscura	1	0	0	0	0	0	0	0	1	1	0	0	0	0	0	0	0	0	0	0	0	1	
Drosophila suzukii	0	0	0	0	0	0	0	0	1	1	0	0	0	0	0	0	0	0	0	0	0	1	
Phortica variegata	0	0	0	1	1	0	0	0	0	0	0	0	0	0	0	0	0	0	0	0	0	1	[31]
Empis bicuspidata	0	0	0	0	0	1	0	1	1	0	1	0	0	0	0	0	0	0	0	0	0	1	[32, 33]
Empis chioptera	0	0	0	0	0	1	0	1	1	0	1	0	0	0	0	0	0	0	0	0	0	1	[32, 33]
Empis ciliata	0	0	0	0	0	1	1	0	1	0	1	0	0	0	0	0	0	0	0	0	0	1	[32, 34]
Empis stercorea	0	0	0	0	0	1	0	0	1	1	0	0	0	0	0	0	0	0	0	0	0	1	[32, 35]
Empis tessellata	0	0	0	0	0	1	0	0	1	1	0	0	0	0	0	0	0	0	0	0	0	1	[32, 35]
Empis trigramma	0	0	0	0	0	1	0	1	1	0	1	0	0	0	0	0	0	0	0	0	0	1	[32, 33]
Empis variegata	0	0	0	1	1	1	0	0	0	0	0	0	0	0	0	0	0	0	0	0	0	1	[32, 34]
Hilara sp.	0	0	1	0	0	0	0	0	0	0	0	0	0	0	0	0	0	0	0	0	0	1	n.a.
Rhamphomyia crassirostris	0	0	0	1	1	1	1	0	0	0	0	0	0	0	0	0	0	0	0	0	0	1	[36]
Rhamphomyia sp.	0	0	0	1	1	1	1	0	0	0	0	0	0	0	0	0	0	0	0	0	0	1	n.a.
Empididae sp. 1	0	0	1	0	0	0	0	0	0	0	0	0	0	0	0	0	0	0	0	0	0	1	n.a.
Empididae sp. 2	0	0	1	0	0	0	0	0	0	0	0	0	0	0	0	0	0	0	0	0	0	1	n.a.
Empididae sp. 3	0	0	1	0	0	0	0	0	0	0	0	0	0	0	0	0	0	0	0	0	0	1	n.a.
Fannia pallitibia	0	0	0	1	1	0	0	0	0	0	0	0	0	0	0	0	0	0	0	0	0	1	[37]
Fannia polychaeta	0	0	0	1	1	0	0	0	0	0	0	0	0	0	0	0	0	0	0	0	0	1	[37]
Fannia scalaris	0	0	0	1	1	0	0	0	0	0	0	0	0	0	0	0	0	0	0	0	0	1	[37]
Fannia sp.	0	0	0	1	1	0	0	0	0	0	0	0	0	0	0	0	0	0	0	0	0	1	[37]
Fanniidae sp.	0	0	0	1	1	0	0	0	0	0	0	0	0	0	0	0	0	0	0	0	0	1	[37]
Suillia bicolor	0	0	0	1	1	0	0	0	0	0	0	0	0	0	0	0	0	0	0	0	0	1	[38]
Heleomyzidae sp.	0	0	1	0	0	0	0	0	0	0	0	0	0	0	0	0	0	0	0	0	0	1	n.a.
Lauxaniidae sp. 1	0	0	1	0	0	0	0	0	0	0	0	0	0	0	0	0	0	0	0	0	0	1	n.a.
Lauxaniidae sp. 2	0	0	1	0	0	0	0	0	0	0	0	0	0	0	0	0	0	0	0	0	0	1	n.a.
Antocha vitripennis	0	0	0	1	1	0	0	0	1	0	1	0	0	0	0	0	0	0	0	0	0	2	[39]
Cheilotrichia cinerascens	0	0	0	1	1	1	0	1	0	0	0	0	0	0	0	0	0	0	0	0	0	2	[39]
Dicranomyia tristis	0	0	0	1	1	0	0	0	0	0	0	0	0	0	0	0	0	0	0	0	0	2	[39]
Dicranomyia sp.	0	0	0	1	1	0	0	0	0	0	0	0	0	0	0	0	0	0	0	0	0	2	n.a.
Helius flavus	0	0	0	1	1	1	0	1	1	0	0	0	0	0	0	0	0	0	0	0	0	2	[39]
Limonia nigropunctata	0	0	0	1	1	0	0	0	0	0	0	0	0	0	0	0	0	0	0	0	0	2	[39]
Limonia nubeculosa	0	0	0	1	1	0	0	0	0	0	0	0	0	0	0	0	0	0	0	0	0	2	[39]
Limonia phragmitidis	0	0	0	1	1	0	0	0	1	0	0	0	0	0	0	0	0	0	0	0	0	2	[39]
Limonia sp.	0	0	1	0	0	0	0	0	0	0	0	0	0	0	0	0	0	0	0	0	0	2	n.a.

MOTU	Pest (1) vector (2)	Parasitoid (1)	*Unknown*	Closed habitats	Woodlands	Semi-open habitats	Hedgerows	Shrublands	Open habitats	Cultivated lands	Meadows	Lawns	Mountainous areas	Screes	Slopes	Urban areas	Lowlands	Mesophilous areas	Ubiquitous	Dry areas	Diurnal or flightless	Largeness (size)	**References**
Metalimnobia bifasciata	0	0	0	1	1	0	0	0	0	0	0	0	0	0	0	0	0	0	0	0	0	2	[39]
Phylidorea ferruginea	0	0	0	1	1	0	0	0	0	0	0	0	0	0	0	0	0	0	0	0	0	2	[40]
Rhipidia maculata	0	0	0	1	1	0	0	0	1	0	0	0	0	0	0	0	0	0	0	0	0	2	[39]
Lonchaeidae sp.	1	0	1	0	0	0	0	0	0	0	0	0	0	0	0	0	0	0	0	0	0	1	n.a.
Atherigona orientalis	2	0	1	0	0	0	0	0	0	0	0	0	0	0	0	0	0	0	0	0	0	2	[41]
Coenosia testacea	0	0	0	0	0	1	1	0	0	0	0	0	0	0	0	0	0	0	0	0	0	2	[34]
Hebecnema fumosa	0	0	0	1	1	1	0	0	0	0	0	0	0	0	0	0	0	0	0	0	0	2	[34]
Helina abdominalis	0	0	0	0	0	1	1	0	0	0	0	0	0	0	0	0	0	0	0	0	0	2	[34]
Helina evecta	0	0	0	1	1	1	0	0	0	0	0	0	0	0	0	0	0	0	0	0	0	2	[34]
Helina impuncta	0	0	0	0	0	1	1	0	1	0	1	0	0	0	0	0	0	0	0	0	0	2	[34]
Helina lasiophthalma	0	0	0	1	1	1	1	0	0	0	0	0	0	0	0	0	0	0	0	0	0	2	[34]
Helina pertusa	0	0	0	1	1	0	0	0	0	0	0	0	0	0	0	0	0	0	0	0	0	2	n.a.
Helina reversio	0	0	0	0	0	1	1	0	0	0	0	0	0	0	0	0	0	0	0	0	0	2	[34]
Helina sp.	0	0	1	0	0	0	0	0	0	0	0	0	0	0	0	0	0	0	0	0	0	2	n.a.
Hydrotaea irritans	0	0	0	1	1	1	0	0	1	0	1	0	0	0	0	0	0	0	0	0	0	2	[34]
Musca autumnalis	0	0	0	0	0	0	0	0	1	0	1	0	0	0	0	1	0	0	0	0	0	2	[34]
Muscina levida	0	0	0	0	0	0	0	0	1	0	0	0	0	0	0	0	0	0	0	0	0	2	[38]
Muscina pascuorum	0	0	0	1	1	0	0	0	0	0	0	0	0	0	0	0	0	0	0	0	0	2	[42]
Muscina prolapsa	0	0	0	0	0	0	0	0	0	0	0	0	0	0	0	1	0	0	0	0	0	2	[38]
Phaonia errans	0	0	0	0	0	1	1	0	0	0	0	0	0	0	0	0	0	0	0	0	0	2	[34]
Phaonia pallida	0	0	0	1	1	0	0	0	1	0	0	0	0	0	0	0	0	0	0	0	0	2	[38]
Phaonia rufiventris	0	0	0	0	0	1	1	0	0	0	0	0	0	0	0	0	0	0	0	0	0	2	[34]
Phaonia subventa	0	0	0	1	1	0	0	0	0	0	0	0	0	0	0	0	0	0	0	0	0	2	[38]
Phaonia trimaculata	0	0	0	1	1	1	1	0	0	0	0	0	0	0	0	0	0	0	0	0	0	2	[34]
Phaonia tuguriorum	0	0	0	0	0	0	0	0	0	0	0	0	0	0	0	1	0	0	0	0	0	2	[38]
Phaonia sp.	0	0	1	0	0	0	0	0	0	0	0	0	0	0	0	0	0	0	0	0	0	2	n.a.
Polietes lardarius	0	0	0	1	1	1	1	0	1	0	1	0	0	0	0	0	0	0	0	0	0	2	[34]
Stomoxys calcitrans	0	0	0	0	0	1	1	0	1	0	1	0	0	0	0	1	0	0	0	0	0	2	[34]
Muscidae sp.	0	0	1	0	0	0	0	0	0	0	0	0	0	0	0	0	0	0	0	0	0	2	n.a.
Leia fascipennis	0	0	0	1	1	0	0	0	0	0	0	0	0	0	0	0	0	0	0	0	0	1	[43]
Geomyza tripunctata	0	0	0	0	0	0	0	0	1	0	0	0	0	0	0	0	0	0	0	0	0	1	[31]
Psilidae sp. 1	0	0	1	0	0	0	0	0	0	0	0	0	0	0	0	0	0	0	0	0	0	1	n.a.
Psilidae sp. 2	0	0	1	0	0	0	0	0	0	0	0	0	0	0	0	0	0	0	0	0	0	1	n.a.
Chrysopilus asiliformis	0	1	1	0	0	0	0	0	0	0	0	0	0	0	0	0	0	0	0	0	0	1	n.a.
Stomorhina lunata	0	1	0	0	0	0	0	0	1	0	1	0	0	0	0	0	0	0	0	0	0	2	[34]
Sarcophaga agnata	0	1	0	0	0	0	0	0	1	0	0	0	0	0	0	0	0	0	0	0	0	2	[44, 45]
Sarcophaga carnaria	0	1	0	0	0	1	1	0	1	0	1	0	0	0	0	0	0	0	0	0	0	2	[44, 38]
Sarcophaga incisilobata	0	1	0	1	1	0	0	0	0	0	0	0	0	0	0	1	0	0	1	0	0	2	[44, 45]
Sarcophaga subvicina	0	0	0	0	0	0	0	0	1	0	0	0	0	0	0	1	0	0	0	0	0	2	[44, 38]
Sarcophaga sp.	0	1	1	0	0	0	0	0	0	0	0	0	0	0	0	0	0	0	0	0	0	2	[44]
Scathophaga stercoraria	0	0	0	0	0	0	0	0	1	1	1	0	0	0	0	0	0	0	0	0	0	2	n.a.
Bradysia trivittata	0	0	0	0	0	0	0	0	1	1	0	0	0	0	0	0	0	0	1	0	0	1	[46]
Simulium vernum	0	0	1	0	0	0	0	0	0	0	0	0	0	0	0	0	0	0	0	0	0	1	n.a.
Sargus bipunctatus	0	0	0	0	0	0	0	0	1	0	1	0	0	0	0	0	0	0	0	0	0	1	[47]
Dasysyrphus albostriatus	0	0	0	1	1	0	0	0	0	0	0	0	0	0	0	0	0	0	0	0	0	2	[48]
Episyrphus balteatus	0	0	0	0	0	0	0	0	0	0	0	0	0	0	0	0	0	0	1	0	0	2	[48]
Eristalis pertinax	0	0	0	1	1	1	0	0	1	1	0	0	0	0	0	0	0	0	0	0	0	2	[48]
Eupeodes lapponicus	0	0	0	1	1	0	0	0	0	0	0	0	0	0	0	0	0	0	0	0	0	2	[48]
Melanostoma mellinum/scalare	0	0	0	0	0	1	0	0	1	0	0	0	0	0	0	0	0	0	0	0	0	2	[48]
Melanostoma scalare	0	0	0	1	1	0	0	0	0	0	0	0	0	0	0	0	0	0	0	0	0	2	[48]
Meliscaeva auricollis	0	0	0	1	1	0	0	0	0	0	0	0	0	0	0	0	0	0	0	0	0	2	[48]
Meliscaeva cinctella	0	0	0	1	1	1	0	1	0	0	0	0	0	0	0	0	0	0	0	0	0	2	[48]
Parasyrphus punctulatus	0	0	0	1	1	1	0	0	0	0	0	0	0	0	0	0	0	0	0	0	0	2	[48]
Platycheirus scutatus	0	0	0	1	1	1	0	1	0	0	0	0	0	0	0	0	0	0	0	0	0	2	[48]
Syrphus ribesii	0	0	0	1	1	1	0	0	1	1	0	0	0	0	0	0	0	0	0	0	0	2	[48]
Syrphus torvus	0	0	0	1	1	1	0	0	0	0	0	0	0	0	0	0	0	0	0	0	0	2	[48]

**MOTU**	Pest (1) vector (2)	Parasitoid (1)	*Unknown*	Closed habitats	Woodlands	Semi-open habitats	Hedgerows	Shrublands	Open habitats	Cultivated lands	Meadows	Lawns	Mountainous areas	Screes	Slopes	Urban areas	Lowlands	Mesophilous areas	Ubiquitous	Dry areas	Diurnal or flightless	Largeness (size)	**References**
Syrphus vitripennis	0	0	0	1	1	1	0	0	0	0	0	0	0	0	0	0	0	0	0	0	0	2	[48]
Syrphidae sp.	0	0	1	0	0	0	0	0	0	0	0	0	0	0	0	0	0	0	0	0	0	2	n.a.
Atylotus loewianus	0	0	1	0	0	0	0	0	0	0	0	0	0	0	0	0	0	0	0	0	0	2	n.a.
Tabanus bromius	0	1	1	0	0	0	0	0	0	0	0	0	0	0	0	0	0	0	0	0	0	2	n.a.
Allophorocera ferruginea	0	1	0	1	1	1	1	0	1	0	1	0	0	0	0	0	0	0	0	0	0	2	[49, 34]
Blepharomyia pagana	0	1	0	0	0	0	0	0	0	0	0	0	0	0	0	0	0	0	0	0	0	2	[49]
Blondelia nigripes	0	1	0	0	0	1	1	1	1	0	1	0	0	0	0	0	0	0	0	0	0	2	[49, 50]
Cyzenis albicans	0	1	0	1	1	1	1	1	0	0	0	0	0	0	0	0	0	0	0	0	0	2	[49]
Eloceria delecta	0	1	0	1	1	1	0	1	0	0	0	0	0	0	0	0	0	0	0	1	0	2	[49]
Kirbya moerens	0	1	0	1	1	1	0	0	1	0	1	0	0	0	0	0	0	0	0	0	0	2	[49, 50]
Linnaemya tessellans	0	1	0	1	1	1	1	1	1	0	1	0	0	0	0	0	0	0	0	0	0	2	[49, 34]
Loewia foeda	0	1	0	1	1	1	0	1	1	0	1	0	0	0	0	0	0	0	0	0	0	2	[49]
Lypha dubia	0	1	0	1	1	1	1	0	0	0	0	0	0	0	0	0	0	0	0	0	0	2	[49, 34]
Meigenia mutabilis	0	1	0	1	1	1	1	1	0	0	0	0	0	0	0	0	0	0	0	0	0	2	[49]
Ocytata pallipes	0	1	0	1	1	1	0	1	1	0	1	0	0	0	0	0	0	0	0	0	0	2	[49]
Oswaldia muscaria	0	1	0	1	1	1	1	0	0	0	0	0	0	0	0	0	0	0	0	0	0	2	[49]
Pales pavida	0	1	0	0	0	1	1	1	1	0	1	0	0	0	0	0	0	0	0	0	0	2	[49]
Peribaea tibialis	0	1	0	1	1	1	0	1	1	0	1	0	0	0	0	0	0	0	0	1	0	2	[49, 51]
Phorocera assimilis	0	1	0	1	1	1	0	0	0	0	0	0	0	0	0	0	0	0	0	0	0	2	[49]
Phryno vetula	0	1	0	1	1	1	0	1	0	0	0	0	0	0	0	0	0	0	0	0	0	2	[49, 34]
Phryxe vulgaris	0	1	0	0	0	1	1	1	1	1	1	0	0	0	0	0	0	0	0	0	0	2	[49, 34]
Pseudoperichaeta nigrolineata	0	1	0	1	1	1	0	1	0	0	0	0	0	0	0	0	0	0	0	0	0	2	[49]
Ramonda prunaria	0	1	0	1	1	1	0	1	0	0	0	0	1	0	0	0	0	0	0	1	0	2	[49]
Ramonda spathulata	0	1	0	1	1	1	0	1	1	0	1	0	0	0	0	0	0	0	0	0	0	2	[49]
Siphona geniculata	0	1	0	1	1	1	1	0	1	0	1	0	0	0	0	0	0	0	0	0	0	2	[49, 51]
Triarthria setipennis	0	1	0	1	1	1	1	1	0	0	0	0	0	0	0	0	0	0	0	0	0	2	[49, 34]
Voria ruralis	0	1	0	1	1	1	1	1	1	0	1	0	0	0	0	0	0	0	0	0	0	2	[49, 34]
Zaira cinerea	0	1	0	0	0	1	1	0	1	0	1	0	0	0	0	0	0	0	0	0	0	2	[49, 34]
Tachinidae sp. 1	0	1	1	0	0	0	0	0	0	0	0	0	0	0	0	0	0	0	0	0	0	2	[51]
Tachinidae sp. 2	0	1	1	0	0	0	0	0	0	0	0	0	0	0	0	0	0	0	0	0	0	2	[51]
Tachinidae sp. 3	0	1	1	0	0	0	0	0	0	0	0	0	0	0	0	0	0	0	0	0	0	2	[51]
Anomoia purmunda	0	0	1	0	0	0	0	0	0	0	0	0	0	0	0	0	0	0	0	0	0	1	n.a.
Nephrotoma appendiculata	0	0	0	1	1	0	0	0	0	0	0	0	0	0	0	0	0	0	0	0	0	2	[39]
Nephrotoma flavescens	0	0	0	1	1	0	0	0	0	0	0	0	0	0	0	0	0	0	0	0	0	2	[39]
Nephrotoma scalaris	0	0	0	1	1	0	0	0	0	0	0	0	0	0	0	0	0	0	0	0	0	2	[39]
Tipula fascipennis	0	0	0	1	1	0	0	0	1	0	1	0	0	0	0	0	0	0	0	0	0	2	[39]
Tipula flavolineata	0	0	0	1	1	0	0	0	0	0	0	0	0	0	0	0	0	0	0	0	0	2	[39]
Tipula fulvipennis	0	0	0	1	1	0	0	0	0	0	0	0	0	0	0	0	0	0	0	0	0	2	[39]
Tipula helvola	0	0	0	1	1	0	0	0	0	0	0	0	0	0	0	0	0	0	0	1	0	2	[39]
Tipula cf. *hortorum*	0	0	0	1	1	0	0	0	0	0	0	0	0	0	0	0	0	0	0	0	0	2	[39]
Tipula lateralis	0	0	0	1	1	0	0	0	1	0	1	0	0	0	0	0	0	0	0	0	0	2	[39]
Tipula cf. *limbata*	0	0	0	1	1	0	0	0	0	0	0	0	0	0	0	0	0	0	0	0	0	2	[39]
Tipula luna	0	0	0	1	1	0	0	0	1	0	0	0	0	0	0	0	0	0	0	0	0	2	[39]
Tipula lunata	0	0	0	1	1	1	0	1	1	0	1	0	0	0	0	0	0	0	0	0	0	2	[39]
Tipula cf. *magnicauda*	0	0	0	1	1	0	0	0	0	0	0	0	0	0	0	0	0	0	0	0	0	2	[39]
Tipula maxima	0	0	0	1	1	0	0	0	0	0	0	0	0	0	0	0	0	0	0	0	0	2	[39]
Tipula oleracea/*recondita*	0	0	0	1	1	0	0	0	1	0	1	0	0	0	0	0	0	0	1	0	0	2	[39]
Tipula pagana	0	0	0	1	1	0	0	0	0	0	0	0	0	0	0	0	0	0	0	0	0	2	[39]
Tipula paludosa	0	0	0	1	1	0	0	0	1	0	0	0	0	0	0	0	0	0	0	0	0	2	[39]
Tipula peliostigma	0	0	0	1	1	0	0	0	0	0	0	0	0	0	0	0	0	0	0	0	0	2	[39]
Tipula scripta	0	0	0	1	1	0	0	0	0	0	0	0	0	0	0	0	0	0	1	0	0	2	[39]
Tipula submarmorata	0	0	0	1	1	0	0	0	0	0	0	0	0	0	0	0	0	0	0	0	0	2	[39]
Tipula cf. *truncorum*	0	0	0	1	1	0	0	0	0	0	0	0	0	0	0	0	0	0	0	0	0	2	[39]
Tipula vernalis	0	0	0	0	0	0	0	0	0	0	0	0	0	0	0	0	0	0	1	0	0	2	[39]
Tipula sp. 1	0	0	0	1	1	0	0	0	0	0	0	0	0	0	0	0	0	0	0	0	0	2	[39]
Tipula sp. 2	0	0	0	1	1	0	0	0	0	0	0	0	0	0	0	0	0	0	0	0	0	2	[39]

**MOTU**	Pest (1) vector (2)	Parasitoid (1)	*Unknown*	Closed habitats	Woodlands	Semi-open habitats	Hedgerows	Shrublands	Open habitats	Cultivated lands	Meadows	Lawns	Mountainous areas	Screes	Slopes	Urban areas	Lowlands	Mesophilous areas	Ubiquitous	Dry areas	Diurnal or flightless	Largeness (size)	**References**
Tipula sp. 3	0	0	0	1	1	0	0	0	0	0	0	0	0	0	0	0	0	0	0	0	0	2	[39]
Physiphora alceae	0	0	0	0	0	0	0	0	1	0	1	0	0	0	0	0	0	0	0	0	0	1	[25, 34]
Diptera sp. 1	0	0	1	0	0	0	0	0	0	0	0	0	0	0	0	0	0	0	0	0	0	2	n.a.
Diptera sp. 2	0	0	1	0	0	0	0	0	0	0	0	0	0	0	0	0	0	0	0	0	0	2	n.a.
Diptera sp. 3	0	0	1	0	0	0	0	0	0	0	0	0	0	0	0	0	0	0	0	0	0	2	n.a.
Diptera sp. 4	0	0	1	0	0	0	0	0	0	0	0	0	0	0	0	0	0	0	0	0	0	2	n.a.
Diptera sp. 5	0	0	1	0	0	0	0	0	0	0	0	0	0	0	0	0	0	0	0	0	0	2	n.a.
Diptera sp. 6	0	0	1	0	0	0	0	0	0	0	0	0	0	0	0	0	0	0	0	0	0	2	n.a.
Diptera sp. 7	0	0	1	0	0	0	0	0	0	0	0	0	0	0	0	0	0	0	0	0	0	2	n.a.
Acanthosomatidae sp.	1	0	1	0	0	0	0	0	0	0	0	0	0	0	0	0	0	0	0	0	0	2	n.a.
Tuberculatus querceus	0	0	0	1	1	0	0	0	0	0	0	0	0	0	0	0	0	0	0	0	0	1	[20]
Aphrophora alni	0	0	0	0	0	1	0	1	0	0	0	0	0	0	0	0	0	0	1	0	0	1	[52]
Philaenus spumarius	0	0	0	0	0	0	0	0	1	1	1	0	0	0	0	0	0	0	0	0	0	1	[52]
Iassus lanio	0	0	0	1	1	0	0	0	0	0	0	0	0	0	0	0	0	0	0	0	0	1	[52]
Cicadellidae sp.	0	0	1	0	0	0	0	0	0	0	0	0	0	0	0	0	0	0	0	0	0	1	n.a.
Adelphocoris lineolatus	0	0	0	0	0	0	0	0	1	0	0	0	0	0	0	0	0	0	0	0	0	1	
Adelphocoris seticornis	0	0	0	0	0	0	0	0	1	0	0	0	0	0	0	0	0	0	0	0	0	1	
Lygus cf. *pratensis*	0	0	0	0	0	0	0	0	1	0	1	0	0	0	0	0	0	0	0	0	0	1	[53]
Neolygus viridis	0	0	0	0	0	0	0	0	0	0	0	0	0	0	0	0	0	0	0	0	0	1	[53]
Orthops kalmii	0	0	0	1	1	1	0	0	1	0	0	0	0	0	0	0	0	0	0	0	0	1	[53]
Orthotylus prasinus	0	0	0	1	1	0	0	0	0	0	0	0	0	0	0	0	0	0	0	0	0	1	[53]
Orthotylus viridinervis	0	0	0	1	1	0	0	0	0	0	0	0	0	0	0	0	0	0	0	0	0	1	[53]
Phytocoris dimidiatus	0	0	0	1	1	0	0	0	0	0	0	0	0	0	0	0	0	0	0	0	0	1	[53]
Phytocoris longipennis	0	0	0	1	1	0	0	0	0	0	0	0	0	0	0	0	0	0	0	0	0	1	[53]
Phytocoris tiliae	0	0	0	1	1	0	0	0	0	0	0	0	0	0	0	0	0	0	0	0	0	1	[53]
Pinalitus cervinus	0	1	0	1	1	0	0	0	0	0	0	0	0	0	0	0	0	0	0	0	0	1	[53]
Charmon sp.	0	1	1	0	0	0	0	0	0	0	0	0	0	0	0	0	0	0	0	0	0	1	n.a.
Microplitis sp. 1	0	1	0	0	0	0	0	0	0	0	0	0	0	0	0	0	0	0	1	0	0	1	[54]
Microplitis sp. 2	1	0	0	0	0	0	0	0	0	0	0	0	0	0	0	0	0	0	1	0	0	1	[54]
Braconidae sp. 1	0	1	1	0	0	0	0	0	0	0	0	0	0	0	0	0	0	0	0	0	0	1	n.a.
Braconidae sp. 2	0	1	1	0	0	0	0	0	0	0	0	0	0	0	0	0	0	0	0	0	0	1	n.a.
Andricus quercusradicis	0	1	0	1	1	0	0	0	0	0	0	0	0	0	0	0	0	0	0	0	0	1	[20]
Campoplex rothii	0	0	1	0	0	0	0	0	0	0	0	0	0	0	0	0	0	0	0	0	0	1	n.a.
Diphyus sp.	0	1	1	0	0	0	0	0	0	0	0	0	0	0	0	0	0	0	0	0	0	1	n.a.
Enicospilus ramidulus	0	1	0	1	1	1	1	0	0	0	0	0	0	0	0	0	0	0	0	0	0	2	[34]
Ichneumonidae sp. 1	0	0	1	0	0	0	0	0	0	0	0	0	0	0	0	0	0	0	0	0	0	2	n.a.
Ichneumonidae sp. 2	0	0	1	0	0	0	0	0	0	0	0	0	0	0	0	0	0	0	0	0	0	2	n.a.
Ichneumonidae sp. 3	0	0	1	0	0	0	0	0	0	0	0	0	0	0	0	0	0	0	0	0	0	2	n.a.
Perilampidae sp.	0	0	1	0	0	0	0	0	0	0	0	0	0	0	0	0	0	0	0	0	0	1	n.a.
Pteromalidae sp.	0	0	1	0	0	0	0	0	0	0	0	0	0	0	0	0	0	0	0	0	0	1	n.a.
Periclista pubescens	0	0	0	1	1	0	0	0	0	0	0	0	0	0	0	0	0	0	0	0	0	1	[55]
Polistes dominula	0	0	0	1	1	0	0	0	1	0	0	0	0	0	0	0	0	0	0	0	0	2	[56]
Nematopogon swammerdamella	0	0	0	1	1	0	0	0	0	0	0	0	0	0	0	0	0	0	0	0	0	1	[57]
Blastobasis phycidella	0	0	0	1	1	0	0	0	0	0	0	0	0	0	0	0	0	0	0	0	0	1	[58]
Diurnea fagella	0	0	0	1	1	0	0	0	0	0	0	0	0	0	0	0	0	0	0	0	0	2	[58]
Carcina quercana	0	0	0	1	1	1	1	0	0	0	0	0	0	0	0	0	0	0	0	0	0	1	[58]
Depressaria douglasella	0	0	0	0	0	1	0	0	1	0	0	0	0	0	0	0	0	0	0	0	0	1	[58]
Cymatophorina diluta	0	0	0	1	1	1	0	0	0	0	0	0	0	0	0	0	0	0	0	0	0	2	[59, 54]
Habrosyne pyritoides	0	0	0	0	0	1	1	0	1	0	0	0	0	0	0	0	0	0	0	0	0	2	[59, 54]
Polyploca ridens	0	0	0	1	1	0	0	0	0	0	0	0	0	0	0	0	0	0	0	0	0	2	[59, 54]
Tethea ocularis	0	0	0	1	1	1	0	0	0	0	0	0	0	0	0	0	0	0	0	0	0	2	[59, 54]
Tethea or	0	0	0	1	1	1	0	0	0	0	0	0	0	0	0	0	0	0	0	0	0	2	[59, 54]
Thyatira batis	0	0	0	1	1	1	0	1	0	0	0	0	0	0	0	0	0	0	0	0	0	2	[59, 54]
Aedia funesta	0	0	0	0	1	1	0	0	1	1	0	0	0	0	0	0	0	0	0	0	0	2	[60, 54]
Arctia caja	0	0	0	1	1	1	1	1	0	0	1	0	0	0	0	0	0	0	0	0	0	3	[59, 54]
Catephia alchymista	0	0	0	1	1	0	0	0	1	0	1	0	0	0	0	0	0	0	0	1	0	3	[60, 54]

MOTU	Pest (1) vector (2)	Parasitoid (1)	*Unknown*	Closed habitats	Woodlands	Semi-open habitats	Hedgerows	Shrublands	Open habitats	Cultivated lands	Meadows	Lawns	Mountainous areas	Screes	Slopes	Urban areas	Lowlands	Mesophilous areas	Ubiquitous	Dry areas	Diurnal or flightless	Largeness (size)	**References**
Catocala nupta	0	0	0	1	1	0	0	0	0	0	0	0	0	0	0	0	0	0	0	0	0	3	[60, 54]
Catocala promissa	0	0	0	1	1	0	0	0	0	0	0	0	0	0	0	0	0	0	0	0	0	3	[60, 54]
Catocala sponsa	0	0	0	1	1	0	0	0	0	0	0	0	0	0	0	0	0	0	0	0	0	3	[60, 54]
Coscinia cribraria	0	0	0	0	0	0	0	0	1	0	0	1	0	0	0	0	0	0	0	0	0	2	[59, 54]
Diacrisia sannio	0	0	0	0	0	0	0	0	1	0	1	0	0	0	0	0	0	0	0	0	0	2	[59, 54]
Diaphora mendica	0	0	0	1	1	1	0	0	0	0	1	0	0	0	0	1	0	0	0	0	0	2	[59, 54]
Dysgonia algira	0	0	0	1	1	1	0	0	0	0	0	0	0	0	0	1	0	0	0	0	0	3	[60, 54]
Euplagia quadripunctaria	1	0	0	1	1	1	0	0	0	0	0	0	0	0	0	1	0	0	0	0	0	3	[59, 54]
Euproctis chrysorrhoea	0	0	0	1	1	1	1	0	0	0	0	0	0	0	0	0	0	0	0	0	0	2	[59, 54]
Herminia tarsicrinalis	0	0	0	1	1	0	0	0	1	0	0	0	0	0	0	0	0	0	0	0	0	2	[54, 61]
Herminia tarsipennalis	0	0	0	1	1	0	0	0	0	0	0	0	0	0	0	0	0	0	0	0	0	2	[54, 61]
Hypena proboscidalis	0	0	0	0	0	1	0	0	1	0	0	0	0	0	0	0	0	0	0	0	0	2	[54, 61]
Laspeyria flexula	0	0	0	1	1	0	0	0	0	0	0	0	0	0	0	0	0	0	0	0	0	2	[54, 62]
Lygephila pastinum	0	0	0	0	0	0	0	0	1	0	0	0	0	0	0	0	0	0	0	0	0	3	[60, 54]
Paracolax tristalis	0	0	0	1	1	0	0	0	0	0	0	0	0	0	0	0	0	0	0	0	0	2	[54, 61]
Phragmatobia fuliginosa	0	0	0	1	1	1	0	0	0	0	0	0	0	0	0	0	0	0	0	0	0	2	[59, 54]
Polypogon plumigeralis	0	0	0	1	1	0	0	0	0	0	0	0	0	0	0	0	0	0	0	0	0	2	[54, 61]
Polypogon strigilata	0	0	0	1	1	0	0	0	0	0	0	0	0	0	0	0	0	0	0	0	0	2	[54, 61]
Rivula sericealis	0	0	0	1	1	0	0	0	1	0	0	0	0	0	0	0	0	0	0	0	0	1	[54, 61]
Scoliopteryx libatrix	0	0	0	1	1	1	0	0	0	0	0	0	0	0	0	0	0	0	0	0	0	3	[60, 54]
Spilarctia lutea	0	0	0	0	0	1	0	0	0	0	0	0	0	0	0	0	0	0	1	0	0	2	[59, 54]
Spilosoma lubricipeda	0	0	0	1	1	1	0	0	1	0	0	0	0	0	0	0	0	0	1	0	0	2	[59, 54]
Trisateles emortualis	0	0	0	1	1	1	0	1	0	0	0	0	0	0	0	0	0	0	0	0	0	2	[54, 61]
Tyta luctuosa	0	0	0	0	0	1	0	0	1	0	1	0	0	0	0	0	0	0	0	1	0	1	[60, 54]
Bryotropha terrella	0	0	0	0	0	0	0	0	1	0	0	0	0	0	0	0	0	0	0	0	0	1	[63, 62]
Dichomeris limosella	0	0	0	0	1	1	0	0	1	0	1	0	0	0	0	0	0	0	0	0	0	1	[64]
Gelechia turpella	0	0	0	1	1	1	0	0	0	0	0	0	0	0	0	0	0	0	0	0	0	1	[65]
Abraxas grossulariata	0	0	0	1	1	1	0	1	0	0	0	0	0	0	0	0	0	0	0	0	0	2	[54, 66]
Agriopis leucophaearia	0	0	0	1	1	0	0	0	0	0	0	0	0	0	0	0	0	0	0	0	0	2	[54, 66]
Agriopis marginaria	0	0	0	1	1	0	0	0	0	0	0	0	0	0	0	0	0	0	0	0	0	2	[54, 66]
Alcis repandata	0	0	0	1	1	1	1	0	0	0	0	0	0	0	0	0	0	0	0	0	0	3	[54, 66]
Aplocera plagiata	0	0	0	1	1	1	0	0	0	0	1	0	0	0	0	0	0	0	0	0	0	2	[54, 66]
Apocheima hispidaria	0	0	0	1	1	1	0	0	0	0	0	0	0	0	0	0	0	0	0	0	0	2	[54, 66]
Cabera exanthemata	0	0	0	1	1	1	1	0	0	0	0	0	0	0	0	0	0	0	0	0	0	2	[54, 66]
Cabera pusaria	0	0	0	1	1	0	0	0	1	0	1	0	0	0	0	0	0	0	0	0	0	2	[54, 66]
Campaea margaritata	0	0	0	0	1	1	0	0	0	0	0	0	0	0	0	0	0	0	0	0	0	3	[54, 66]
Camptogramma bilineata	0	0	0	0	0	1	0	1	0	0	0	0	0	0	0	1	0	0	0	0	0	2	[54, 66]
Charissa italohelveticus	0	0	0	0	0	0	0	0	0	0	0	0	1	0	0	0	0	0	0	0	0	2	[67, 54]
Chiasmia clathrata	0	0	0	0	0	1	0	0	1	0	1	0	0	0	0	0	0	0	0	0	0	2	[54, 66]
Chlorissa viridata	0	0	0	0	0	1	0	0	0	0	0	0	0	0	0	0	0	0	0	1	0	1	[54, 66]
Chloroclysta siterata	0	0	0	1	1	1	1	0	0	0	0	0	0	0	0	0	0	0	0	0	0	2	[54, 66]
Colotois pennaria	0	0	0	1	1	1	1	0	0	0	0	0	0	0	0	0	0	0	0	0	0	3	[54, 66]
Crocallis elinguaria	0	0	0	0	1	1	0	1	0	0	0	0	0	0	0	0	0	0	0	0	0	2	[54, 66]
Cyclophora linearia	0	0	0	1	1	0	0	0	0	0	0	0	0	0	0	0	0	0	0	0	0	2	[54, 66]
Cyclophora punctaria	0	0	0	1	1	0	0	0	0	0	0	0	0	0	0	0	0	0	0	0	0	2	[54, 66]
Deileptenia ribeata	0	0	0	1	1	0	0	0	0	0	0	0	0	0	0	0	0	0	0	0	0	3	[54, 66]
Dysstroma truncata	0	0	0	0	1	1	1	0	0	0	0	0	0	0	0	0	0	0	0	0	0	2	[54, 66]
Ectropis crepuscularia	0	0	0	1	1	1	1	0	0	0	0	0	0	0	0	0	0	0	0	0	0	2	[54, 66]
Elophos dilucidaria	0	0	0	0	0	0	0	0	0	0	0	0	1	0	0	0	0	0	0	0	0	2	[54, 66]
Ematurga atomaria	0	0	0	0	1	1	0	0	1	0	1	0	0	0	0	0	0	0	0	1	0	2	[54, 66]
Ennomos quercinaria	0	0	0	1	1	1	0	0	0	0	0	0	0	0	0	0	0	0	0	0	0	2	[54, 66]
Epirrhoe alternata	0	0	0	1	1	1	0	0	0	0	1	0	0	0	0	0	0	0	0	0	0	1	[54, 66]
Epirrita christyi	0	0	0	1	1	1	1	0	0	0	0	0	0	0	0	0	0	0	0	0	0	2	[54, 66]
Erannis defoliaria	0	0	0	1	1	1	1	0	0	0	0	0	0	0	0	0	0	0	0	0	0	2	[54, 66]
Eupithecia abbreviata	0	0	0	1	1	1	1	0	0	0	0	0	0	0	0	0	0	0	0	0	0	1	[54, 66]
Eupithecia dodoneata	0	0	0	0	1	1	0	0	0	0	0	0	0	0	0	0	0	0	0	0	0	1	[54, 66]

MOTU	Pest (1) vector (2)	Parasitoid (1)	*Unknown*	Closed habitats	Woodlands	Semi-open habitats	Hedgerows	Shrublands	Open habitats	Cultivated lands	Meadows	Lawns	Mountainous areas	Screes	Slopes	Urban areas	Lowlands	Mesophilous areas	Ubiquitous	Dry areas	Diurnal or flightless	Largeness (size)	**References**
Eupithecia haworthiata	0	0	0	1	1	1	1	0	0	0	0	0	0	0	0	0	0	0	0	0	0	1	[54, 66]
Eupithecia tantillaria	0	0	0	1	1	1	0	0	0	0	0	0	0	0	0	0	0	0	0	0	0	1	[54, 66]
Gnophos furvata	0	0	0	0	0	1	0	1	1	0	0	0	0	1	1	0	0	0	0	1	0	3	[54, 66]
Hemistola chrysoprasaria	0	0	0	1	1	1	1	0	1	0	0	0	1	0	1	0	0	0	0	0	0	2	[54, 66]
Hemithea aestivaria	0	0	0	1	1	1	1	0	0	0	0	0	0	0	0	0	0	0	0	0	0	1	[54, 66]
Horisme radicaria	0	0	0	0	0	1	1	0	1	0	0	0	0	1	1	0	0	0	0	0	0	2	[54, 66]
Hydriomena furcata	0	0	0	1	1	0	0	0	0	0	0	0	0	0	0	0	0	0	0	0	0	2	[54, 66]
Hypomecis roboraria	0	0	0	1	1	0	0	0	0	0	0	0	0	0	0	0	1	0	0	0	0	3	[54, 66]
Idaea aversata	0	0	0	1	1	1	1	0	0	0	0	0	0	0	0	0	0	0	1	0	0	2	[54, 66]
Idaea dimidiata	0	0	0	1	1	1	1	0	0	0	0	0	0	0	0	0	0	0	0	0	0	1	[54, 66]
Idaea fuscovenosa	0	0	0	0	0	1	1	0	0	0	0	0	0	0	0	1	0	0	0	0	0	1	[54, 66]
Idaea humiliata	0	0	0	0	0	1	0	0	1	0	0	0	0	0	1	0	0	0	0	0	0	1	[54, 66]
Idaea ochrata	0	0	0	0	0	1	0	0	1	0	0	0	0	0	0	0	0	0	0	0	0	1	[54, 66]
Idaea rusticata	0	0	0	1	1	1	1	0	1	0	0	0	0	0	0	0	0	0	0	0	0	1	[54, 66]
Idaea seriata	0	0	0	0	0	1	0	0	0	0	0	0	0	0	0	1	0	0	1	0	0	1	[54, 66]
Idaea straminata	0	0	0	0	1	1	0	0	1	0	0	0	0	0	0	0	0	0	0	0	0	2	[54, 66]
Jodis lactearia	0	0	0	1	1	1	0	0	0	0	0	0	0	0	0	0	0	0	0	0	0	1	[54, 66]
Ligdia adustata	0	0	0	1	1	1	1	1	1	0	0	0	0	0	1	0	0	0	0	1	0	2	[54, 66]
Lobophora halterata	0	0	0	1	1	0	0	0	0	0	0	0	0	0	0	0	0	0	0	0	0	1	[54, 66]
Lomaspilis marginata	0	0	0	1	1	1	1	0	0	0	0	0	0	0	0	0	0	0	0	0	0	1	[54, 66]
Lomographa temerata	0	0	0	1	1	1	1	1	0	0	0	0	0	0	0	0	0	0	0	0	0	2	[54, 66]
Lycia hirtaria	0	0	0	1	1	1	1	0	0	0	0	0	0	0	0	0	0	0	0	0	0	3	[54, 66]
Macaria liturata	0	0	0	1	1	1	0	0	0	0	0	0	0	0	0	0	0	0	0	0	0	2	[54, 66]
Melanthia procellata	0	0	0	0	1	1	1	0	0	0	0	0	0	0	0	0	0	0	0	0	0	2	[54, 66]
Menophra abruptaria	0	0	0	1	1	1	1	0	0	0	0	0	0	0	0	0	0	0	0	0	0	2	[54, 66]
Nychiodes obscuraria	0	0	0	1	1	1	0	0	1	0	0	0	0	1	1	0	0	0	0	0	0	3	[54, 66]
Odontopera bidentata	0	0	0	1	1	0	0	0	0	0	0	0	0	0	0	0	0	0	0	0	0	2	[54, 66]
Operophtera brumata	0	0	0	1	1	1	1	0	1	0	0	0	0	0	0	1	0	0	0	0	0	2	[54, 66]
Operophtera fagata	0	0	0	1	1	1	1	0	0	0	0	0	0	0	0	0	0	0	0	0	0	2	[54, 66]
Opisthograptis luteolata	0	0	0	1	1	1	1	0	0	0	1	0	0	0	0	0	0	0	0	0	0	2	[54, 66]
Pasiphila chloerata	0	0	0	1	1	1	1	0	0	0	0	0	0	0	0	0	0	0	0	0	0	1	[54, 66]
Pasiphila rectangulata	0	0	0	0	1	1	1	0	0	0	0	0	0	0	0	0	0	0	0	0	0	1	[54, 66]
Pelurga comitata	0	0	0	1	1	1	0	0	0	0	0	0	0	0	0	0	0	0	0	0	0	2	[54, 66]
Peribatodes rhomboidaria	0	0	0	0	1	1	1	0	0	0	0	0	0	0	0	1	0	0	0	0	0	2	[54, 66]
Peribatodes secundaria	0	0	0	1	1	0	0	0	1	0	0	0	0	0	1	0	0	0	0	0	0	2	[54, 66]
Plagodis dolabraria	0	0	0	1	1	1	0	0	0	0	0	0	0	0	0	0	0	0	0	0	0	2	[54, 66]
Rhodometra sacraria	0	0	0	0	0	1	0	0	0	0	0	0	0	0	0	0	0	0	0	0	0	1	[54, 66]
Scopula immorata	0	0	0	1	1	1	0	0	1	0	1	0	0	0	0	0	0	0	0	1	0	1	[54, 66]
Scopula immutata	0	0	0	1	1	1	0	0	1	0	1	0	0	0	0	0	0	0	0	0	0	1	[54, 66]
Scopula virgulata	0	0	0	0	1	1	0	0	1	0	0	0	0	0	1	0	0	0	0	0	0	1	[54, 66]
Selenia tetralunaria	0	0	0	1	1	1	0	0	0	0	0	0	0	0	0	0	0	0	0	0	0	2	[54, 66]
Stegania trimaculata	0	0	0	0	0	1	0	0	0	0	0	0	0	0	0	0	0	0	0	0	0	1	[54, 66]
Thalera fimbrialis	0	0	0	0	0	1	0	0	1	0	0	0	0	0	1	0	0	0	0	0	0	2	[54, 66]
Timandra comae	0	0	0	0	0	1	0	0	0	0	1	0	0	0	0	0	0	0	0	0	0	2	[54, 66]
Triphosa dubitata	0	0	0	0	0	1	0	1	1	0	0	0	0	0	1	0	0	0	1	0	0	2	[54, 66]
Xanthorhoe ferrugata	0	0	0	1	1	1	0	0	1	0	1	0	0	0	0	0	0	0	0	0	0	1	[54, 66]
Xanthorhoe fluctuata	0	0	0	0	0	1	0	0	0	0	0	0	0	0	0	1	0	0	0	0	0	1	[54, 66]
Geometridae sp.	0	0	1	0	0	0	0	0	0	0	0	0	0	0	0	0	0	0	0	0	0	2	n.a.
Korscheltellus lupulinus	0	0	0	0	0	1	0	0	1	1	1	0	0	0	0	0	0	0	0	0	0	2	[59, 54]
Triodia sylvina	0	0	0	0	0	1	0	0	1	1	1	0	0	0	0	0	0	0	0	0	0	2	[59, 54]
Lasiocampa quercus	0	0	0	1	1	1	0	0	1	0	1	0	0	0	0	0	0	0	0	0	0	3	[59, 54]
Malacosoma neustria	0	0	0	1	1	1	0	0	0	0	0	0	0	0	0	0	1	0	0	0	0	2	[59, 54]
Apoda limacodes	0	0	0	1	1	0	0	0	0	0	0	0	0	0	0	0	0	0	0	0	0	2	[59, 54]
Abrostola triplasia	0	0	0	0	0	0	0	0	0	0	0	0	0	0	0	0	0	0	1	0	0	2	[60, 54]
Acronicta psi	0	0	0	1	1	1	0	1	0	0	0	0	0	0	0	1	0	1	0	0	0	3	[68, 54]
Acronicta rumicis	0	0	0	1	1	0	0	0	1	0	0	0	0	0	0	1	0	1	0	0	0	2	[68, 54]

**MOTU**	Pest (1) vector (2)	Parasitoid (1)	*Unknown*	Closed habitats	Woodlands	Semi-open habitats	Hedgerows	Shrublands	Open habitats	Cultivated lands	Meadows	Lawns	Mountainous areas	Screes	Slopes	Urban areas	Lowlands	Mesophilous areas	Ubiquitous	Dry areas	Diurnal or flightless	Largeness (size)	**References**
Actinotia polyodon	0	0	0	1	1	1	0	0	1	0	0	1	0	0	0	0	0	1	0	1	0	2	[69, 54]
Agrochola circellaris	0	0	0	1	1	1	0	1	0	0	0	0	0	0	0	1	0	0	0	0	0	2	[70, 54]
Agrochola litura	0	0	0	1	1	1	0	1	1	0	0	0	0	0	0	0	0	0	0	1	0	2	[70, 54]
Agrochola lota	0	0	0	0	0	1	0	0	1	0	0	0	0	0	0	0	0	0	0	0	0	2	[70, 54]
Agrochola lunosa	0	0	0	0	0	0	0	0	1	0	0	0	0	0	0	0	0	0	0	0	0	2	[70, 54]
Agrochola lychnidis	0	0	0	1	1	0	0	0	1	0	0	0	0	0	0	1	0	0	0	0	0	2	[70, 54]
Agrochola pistacinoides	1	0	0	0	0	1	0	1	1	0	0	0	0	0	1	0	0	1	0	0	0	2	[70, 54]
Agrotis bigramma	1	0	0	0	0	1	0	1	1	0	0	0	0	0	0	0	0	0	0	0	0	3	[69, 54]
Agrotis exclamationis	1	0	0	0	0	0	0	0	1	1	0	0	0	0	0	0	0	0	0	0	0	3	[69, 54]
Agrotis ipsilon	1	0	0	0	0	1	0	0	1	1	1	0	0	0	0	0	0	1	0	0	0	3	[69, 54]
Agrotis segetum	0	0	0	0	0	0	0	0	1	0	0	0	0	0	0	0	0	0	1	0	0	2	[69, 54]
Allophyes oxyacanthae	0	0	0	0	1	1	0	1	0	0	0	0	0	0	0	0	0	0	0	0	0	3	[70, 54]
Ammoconia caecimacula	0	0	0	0	1	1	0	0	1	0	0	0	0	0	0	0	0	0	0	0	0	3	[70, 54]
Amphipoea oculea	0	0	0	0	0	0	0	0	1	0	1	0	0	0	0	0	0	0	0	0	0	2	[71, 54]
Amphipyra berbera	0	0	0	0	1	1	0	1	0	0	0	0	0	0	0	0	0	0	0	0	0	3	[68, 54]
Amphipyra pyramidea	0	0	0	0	1	1	0	0	0	0	0	0	0	0	0	1	0	0	0	0	0	3	[68, 54]
Anaplectoides prasina	0	0	0	1	1	0	0	0	1	0	1	0	0	0	0	0	0	0	0	0	0	3	[72, 54]
Anarta trifolii	0	0	0	0	0	0	0	0	1	0	0	0	0	0	0	1	1	0	0	0	0	2	[73, 54, 62]
Anorthoa munda	0	0	0	1	1	1	0	0	0	0	1	0	0	0	0	1	0	0	0	0	0	3	[70, 54]
Apamea aquila	0	0	0	0	1	1	0	0	1	0	0	1	0	0	0	0	0	0	0	0	0	2	[71, 54]
Apamea crenata	0	0	0	1	1	0	0	0	1	0	1	0	0	0	0	0	0	1	0	0	0	2	[71, 54]
Apamea epomidion	0	0	0	1	1	0	0	0	0	0	0	0	0	0	0	0	0	0	0	0	0	3	[71, 54]
Apamea lithoxylaea	0	0	0	0	1	1	0	0	1	0	1	0	0	0	0	1	0	0	0	1	0	3	[71, 54]
Apamea monoglypha	0	0	0	0	0	0	0	0	1	0	0	0	0	0	0	1	0	0	0	0	0	3	[71, 54]
Apamea scolopacina	0	0	0	0	0	1	0	0	1	0	1	0	0	0	0	0	0	1	0	0	0	2	[71, 54]
Apamea sordens	0	0	0	0	0	1	0	0	0	0	1	0	0	0	0	1	0	0	0	0	0	2	[71, 54]
Aporophyla lueneburgensis	0	0	0	0	0	0	0	0	1	0	1	0	0	0	0	0	0	0	0	1	0	2	[70, 54]
Asteroscopus sphinx	0	0	0	1	1	0	0	0	0	0	0	0	0	0	0	0	0	0	0	0	0	3	[74, 54]
Atethmia centrago	0	0	0	1	1	0	0	0	1	0	0	0	0	0	1	1	0	0	0	1	0	2	[70, 54]
Athetis gluteosa	0	0	0	0	0	1	0	0	1	0	1	0	0	0	0	0	0	0	0	0	0	2	[68, 54]
Autographa gamma	0	0	0	0	0	0	0	0	0	0	0	0	0	0	0	0	0	0	1	0	0	3	[60, 54]
Autographa pulchrina	0	0	0	1	1	1	0	0	1	0	1	0	0	0	0	0	0	0	0	0	0	2	[60, 54]
Axylia putris	0	0	0	0	0	0	0	0	1	1	0	0	0	0	0	1	0	0	0	0	0	2	[69, 54]
Bryophila domestica	0	0	0	0	1	1	0	0	1	0	0	1	0	1	1	0	0	0	0	0	0	2	[54, 75]
Calophasia lunula	1	0	0	1	1	1	0	0	1	0	1	1	0	0	0	0	0	0	0	0	0	2	[54, 62]
Caradrina clavipalpis	0	0	0	0	1	1	0	0	1	0	0	0	0	0	0	1	0	0	0	0	0	2	[68, 54]
Charanyca trigrammica	0	0	0	0	0	0	0	0	1	0	1	0	0	0	0	0	0	0	0	0	0	2	[68, 54]
Conistra erythrocephala	0	0	0	1	1	0	0	0	0	0	0	0	0	0	0	0	0	0	0	0	0	2	[70, 54]
Conistra rubiginea	0	0	0	1	1	0	0	0	0	0	0	0	0	0	0	0	0	0	0	0	0	2	[70, 54]
Conistra rubiginosa	0	0	0	1	1	1	0	0	1	1	0	0	0	0	0	1	0	0	1	0	0	2	[70, 54]
Conistra vaccinii	0	0	0	0	1	1	0	0	1	0	0	0	0	0	0	1	0	0	0	0	0	2	[70, 54]
Cosmia affinis	1	0	0	0	1	1	0	0	0	0	0	0	0	0	0	0	1	0	0	0	0	2	[68, 54]
Cosmia pyralina	0	0	0	1	1	0	0	0	0	0	0	0	0	0	0	0	0	0	0	0	0	2	[68, 54]
Cosmia trapezina	0	0	0	1	1	0	0	0	0	0	0	0	0	0	0	1	0	0	0	0	0	2	[68, 54]
Craniophora ligustri	0	0	0	1	1	0	0	0	0	0	0	0	0	0	0	0	1	0	0	0	0	2	[54, 75]
Cryphia algae	0	0	0	1	1	1	0	0	0	0	0	0	0	0	0	0	0	0	0	0	0	1	[54, 75]
Cucullia chamomillae	0	0	0	0	0	1	0	0	1	0	1	0	0	0	0	0	0	1	0	1	0	3	[76, 54]
Cucullia umbratica	0	0	0	0	0	1	0	0	0	0	1	0	0	0	0	0	0	1	0	0	0	3	[76, 54]
Diachrysia stenochrysis	0	0	0	0	0	0	0	0	1	0	0	0	0	0	0	1	0	1	0	0	0	2	[60, 54]
Dichonia aprilina	0	0	0	1	1	0	0	0	0	0	0	0	0	0	0	1	0	0	0	1	0	3	[70, 54]
Dicycla oo	0	0	0	0	1	1	0	0	0	0	0	0	0	0	0	0	0	0	0	0	0	2	[68, 54]
Dryobotodes eremita	0	0	0	1	1	1	0	0	0	0	0	0	0	0	0	1	0	0	0	0	0	2	[70, 54]
Egira conspicillaris	0	0	0	0	1	1	0	0	1	0	1	1	0	0	0	0	0	0	0	0	0	2	[70, 54]
Elaphria venustula	0	0	0	0	1	1	0	0	1	0	0	0	0	0	0	0	0	0	0	0	0	1	[68, 54]
Emmelia trabealis	0	0	0	0	0	1	0	0	1	0	1	0	0	0	0	0	0	0	0	0	0	1	[54, 75]
Episema glaucina	0	0	0	0	0	0	0	0	1	0	0	0	0	0	0	0	0	0	0	1	0	2	[70, 54]

MOTU	Pest (1) vector (2)	Parasitoid (1)	*Unknown*	Closed habitats	Woodlands	Semi-open habitats	Hedgerows	Shrublands	Open habitats	Cultivated lands	Meadows	Lawns	Mountainous areas	Screes	Slopes	Urban areas	Lowlands	Mesophilous areas	Ubiquitous	Dry areas	Diurnal or flightless	Largeness (size)	**References**
Eugnorisma depuncta	0	0	0	1	1	0	0	0	1	0	1	0	0	0	0	0	0	0	0	0	0	2	[69, 54]
Eugnorisma glareosa	0	0	0	1	1	0	0	0	1	0	1	0	0	0	0	0	0	0	0	1	0	2	[72, 54]
Eupsilia transversa	0	0	0	1	1	0	0	0	1	0	0	0	0	0	0	1	0	0	0	0	0	3	[70, 54]
Euxoa aquilina	0	0	0	0	0	0	0	0	1	0	0	1	1	0	1	0	0	0	0	1	0	2	[69, 54]
Euxoa decora	0	0	0	0	0	0	0	0	1	0	0	0	1	0	0	0	0	0	0	0	0	2	[69, 54]
Euxoa recussa	0	0	0	0	0	0	0	0	1	0	0	1	1	0	0	0	0	0	0	0	0	2	[69, 54]
Hadena bicruris	0	0	0	0	0	0	0	0	1	0	0	0	0	0	0	0	0	0	0	0	0	2	[73, 54]
Hadena compta	0	0	0	0	0	1	0	0	1	0	0	1	0	0	1	1	0	0	0	1	0	2	[73, 54]
Hecatera bicolorata	0	0	0	0	0	1	0	0	1	0	0	0	0	0	0	0	0	0	0	0	0	2	[73, 54]
Hecatera dysodea	1	0	0	0	0	1	0	0	0	0	0	0	0	0	0	1	0	0	0	1	0	2	[73, 54]
Helicoverpa armigera	1	0	0	0	0	0	0	0	0	0	0	0	0	0	0	0	0	0	1	0	0	2	[54, 75]
Heliothis peltigera	0	0	0	0	0	0	0	0	1	1	0	0	0	0	0	0	0	0	0	1	0	2	[54, 75]
Hoplodrina ambigua	0	0	0	0	0	0	0	0	1	0	0	0	0	0	0	1	0	0	0	0	0	2	[68, 54, 62]
Hoplodrina blanda	0	0	0	0	0	0	0	0	0	0	0	0	0	0	0	0	0	0	1	0	0	2	[68, 54, 62]
Hoplodrina octogenaria	1	0	0	1	1	0	0	0	1	0	0	1	0	0	0	0	0	0	0	0	0	2	[68, 54]
Lacanobia oleracea	0	0	0	0	0	1	0	0	1	1	0	0	0	0	0	1	0	0	0	0	0	2	[73, 54]
Lacanobia suasa	0	0	0	0	0	0	0	0	1	0	0	0	0	0	0	0	0	0	1	0	0	2	[73, 54]
Lithophane ornitopus	0	0	0	0	1	1	0	0	0	0	0	0	0	0	0	0	0	0	0	1	0	2	[70, 54]
Lithophane semibrunnea	0	0	0	1	1	0	0	0	1	0	0	0	0	0	0	0	0	0	0	0	0	3	[70, 54]
Lithophane socia	0	0	0	1	1	0	0	0	0	0	0	0	0	0	0	0	0	0	0	0	0	3	[70, 54]
Luperina dumerilii	0	0	0	0	0	0	0	0	1	0	1	0	0	0	1	1	0	0	0	0	0	2	[71, 54]
Macdunnoughia confusa	0	0	0	0	1	1	0	0	1	0	0	0	0	0	0	1	0	0	0	0	0	2	[60, 54]
Mamestra brassicae	0	0	0	0	1	1	0	0	1	0	0	0	0	0	0	1	0	0	0	0	0	2	[73, 54]
Melanchra persicariae	0	0	0	1	1	0	0	0	0	0	0	0	0	0	0	1	0	0	0	0	0	2	[73, 54]
Mesapamea secalis/secalella/didyma	0	0	0	0	0	0	0	0	1	0	0	0	0	0	0	1	0	0	0	0	0	2	[71, 54]
Mesoligia furuncula	0	0	0	0	0	0	0	0	1	0	0	0	0	0	0	0	0	0	1	0	0	2	[71, 54]
Mniotype satura	0	0	0	1	1	1	0	1	0	0	0	0	0	0	0	0	0	0	0	0	0	3	[70, 54]
Mormo maura	0	0	0	1	1	1	0	1	0	0	0	0	0	0	0	0	0	0	0	0	0	3	[68, 54]
Mythimna albipuncta	0	0	0	0	0	0	0	0	1	1	0	0	0	0	0	1	0	0	0	0	0	2	[73, 54]
Mythimna conigera	0	0	0	0	0	1	0	0	1	0	1	1	1	0	0	0	0	0	0	0	0	2	[73, 54]
Mythimna ferrago	0	0	0	0	0	0	0	0	1	0	0	0	0	0	0	0	0	0	0	0	0	2	[73, 54]
Mythimna impura	0	0	0	0	0	0	0	0	1	0	1	0	0	0	0	0	0	0	0	0	0	2	[73, 54]
Mythimna l-album	1	0	0	0	0	0	0	0	1	0	1	0	0	0	0	0	0	0	0	0	0	2	[73, 54]
Mythimna loreyi	0	0	0	0	0	0	0	0	1	0	0	0	0	0	0	0	0	0	0	0	0	2	[73, 54]
Mythimna pallens	1	0	0	0	1	1	0	0	1	0	1	0	0	0	0	1	0	0	0	0	0	2	[73, 54]
Mythimna unipuncta	1	0	0	0	0	0	0	0	1	1	0	0	0	0	0	1	0	0	0	0	0	2	[73, 54]
Mythimna vitellina	0	0	0	0	0	0	0	0	1	1	0	0	0	0	0	1	0	0	0	0	0	2	[73, 54]
Noctua comes	0	0	0	1	1	1	0	0	1	0	1	1	0	0	0	1	0	0	0	0	0	3	[72, 54]
Noctua fimbriata	0	0	0	1	1	0	0	0	1	0	0	0	0	0	0	0	0	0	0	0	0	3	[72, 54]
Noctua janthe	0	0	0	0	0	1	0	1	1	0	0	0	0	0	0	1	0	0	0	0	0	3	[72, 54]
Noctua janthina	0	0	0	0	1	1	0	1	1	0	0	0	0	0	0	1	0	0	0	0	0	2	[72, 54]
Noctua orbona	0	0	0	1	1	0	0	0	1	0	0	0	0	0	0	0	0	0	0	0	0	2	[72, 54]
Noctua pronuba	0	0	0	0	0	0	0	0	0	0	0	0	0	0	0	1	0	0	1	0	0	3	[72, 54]
Ochropleura plecta	0	0	0	0	0	0	0	0	1	0	0	0	0	0	0	0	0	0	1	0	0	2	[69, 54]
Oligia latruncula	0	0	0	0	0	0	0	0	1	0	0	0	0	0	0	1	0	0	0	0	0	2	[71, 54]
Oligia strigilis	0	0	0	0	0	0	0	0	1	0	0	0	0	0	0	0	0	0	0	1	0	2	[71, 54]
Oligia versicolor	0	0	0	1	1	0	0	0	1	0	1	0	0	0	0	1	0	0	0	0	0	2	[71, 54]
Orthosia cerasi	0	0	0	1	1	0	0	0	0	0	0	0	0	0	0	1	0	0	0	0	0	2	[70, 54]
Orthosia gothica	0	0	0	1	1	1	0	0	1	0	1	1	0	0	0	0	0	0	0	0	0	2	[70, 54]
Orthosia gracilis/opima	0	0	0	0	0	0	0	0	0	0	0	0	0	0	0	0	0	0	0	0	0	2	[70, 54]
Orthosia incerta	0	0	0	1	1	0	0	0	1	0	1	0	0	0	0	0	0	0	0	0	0	2	[70, 54]
Parastichtis ypsillon	0	0	0	1	1	1	0	0	0	0	0	0	0	0	0	1	0	0	0	0	0	2	[68, 54]
Peridroma saucia	0	0	0	0	0	0	0	0	0	0	0	0	0	0	0	1	0	0	1	0	0	3	[72, 54]
Phlogophora meticulosa	0	0	0	1	1	0	0	0	1	0	0	0	0	0	0	1	0	0	1	0	0	3	[68, 54]
Polia nebulosa	0	0	0	0	1	1	0	1	0	0	0	0	0	0	0	0	0	0	0	0	0	3	[73, 54]
Polyphaenis sericata	1	0	0	1	1	1	0	1	1	0	0	0	0	0	0	0	0	0	0	0	0	2	[68, 54]

MOTU	Pest (1) vector (2)	Parasitoid (1)	*Unknown*	Closed habitats	Woodlands	Semi-open habitats	Hedgerows	Shrublands	Open habitats	Cultivated lands	Meadows	Lawns	Mountainous areas	Screes	Slopes	Urban areas	Lowlands	Mesophilous areas	Ubiquitous	Dry areas	Diurnal or flightless	Largeness (size)	**References**
Protodeltote pygarga	0	0	0	0	1	1	0	0	1	0	1	0	0	0	0	0	0	1	0	0	0	2	[54, 75]
Pyrrhia umbra	0	0	0	0	1	1	0	0	1	0	1	0	0	0	0	1	0	0	0	0	0	2	[54, 75]
Rusina ferruginea	0	0	0	1	1	1	0	0	0	0	0	0	1	0	0	0	0	0	0	0	0	2	[68, 54]
Sideridis reticulata	1	0	0	0	0	0	0	0	1	0	0	1	0	0	1	0	0	0	0	0	0	2	[73, 54]
Spodoptera exigua	0	0	0	0	0	0	0	0	1	1	0	0	0	0	0	0	1	0	0	0	0	2	[68, 54]
Thalpophila matura	0	0	0	0	0	1	0	0	1	0	1	1	0	0	0	0	0	0	0	0	0	3	[68, 54]
Tholera cespitis	0	0	0	0	0	1	0	0	1	0	1	1	0	0	0	0	0	1	0	1	0	2	[73, 54]
Tholera decimalis	0	0	0	0	0	0	0	0	1	0	0	0	1	0	1	0	0	0	0	0	0	2	[73, 54]
Tiliacea aurago	0	0	0	0	1	1	0	0	0	0	0	0	0	0	0	1	0	0	0	1	0	2	[70, 54]
Tiliacea citrago	0	0	0	0	1	1	0	0	0	0	0	0	0	0	0	0	0	0	0	1	0	2	[70, 54]
Trachea atriplicis	1	0	0	0	1	1	0	0	1	0	0	0	0	0	0	1	0	0	0	0	0	3	[68, 54]
Trichoplusia ni	0	0	0	0	0	0	0	0	0	0	0	0	0	0	0	0	0	0	1	0	0	2	[60, 54]
Xanthia gilvago	0	0	0	1	1	0	0	0	0	0	0	0	0	0	0	1	0	0	0	0	0	2	[70, 54]
Xanthia icteritia	0	0	0	1	1	0	0	0	0	0	0	0	0	0	0	1	0	0	0	0	0	2	[70, 54]
Xanthia ocellaris	0	0	0	0	1	1	0	0	0	0	0	0	0	0	0	0	0	0	0	0	0	2	[70, 54]
Xestia baja	0	0	0	1	1	0	0	0	1	0	1	0	0	0	0	0	0	0	0	0	0	3	[72, 54]
Xestia castanea	1	0	0	0	1	1	0	0	1	0	0	0	0	0	1	0	0	0	0	1	0	2	[72, 54]
Xestia c-nigrum	0	0	0	0	0	0	0	0	0	0	0	0	0	0	0	1	0	0	1	0	0	2	[72, 54]
Xestia rhomboidea	0	0	0	1	1	0	0	0	1	0	1	0	0	0	0	0	0	0	0	0	0	2	[72, 54]
Xestia sexstrigata	0	0	0	0	0	1	0	0	1	0	1	1	0	0	0	0	0	0	0	0	0	2	[72, 54]
Xestia xanthographa	0	0	0	0	0	0	0	0	0	0	0	0	0	0	0	1	0	0	1	0	0	2	[72, 54]
Xylena sp.	0	0	0	1	1	0	0	0	0	0	0	0	0	0	0	0	0	0	0	0	0	3	[70, 54]
Noctuidae sp. 1	0	0	1	0	0	0	0	0	0	0	0	0	0	0	0	0	0	0	0	0	0	2	n.a.
Noctuidae sp. 2	0	0	1	0	0	0	0	0	0	0	0	0	0	0	0	0	0	0	0	0	0	2	n.a.
Noctuidae sp. 3	0	0	1	0	0	0	0	0	0	0	0	0	0	0	0	0	0	0	0	0	0	2	n.a.
Noctuidae sp. 4	0	0	1	0	0	0	0	0	0	0	0	0	0	0	0	0	0	0	0	0	0	2	n.a.
Noctuidae sp. 5	0	0	1	0	0	0	0	0	0	0	0	0	0	0	0	0	0	0	0	0	0	2	n.a.
Noctuidae sp. 6	0	0	1	0	0	0	0	0	0	0	0	0	0	0	0	0	0	0	0	0	0	2	n.a.
Noctuidae sp. 7	0	0	1	0	0	0	0	0	0	0	0	0	0	0	0	0	0	0	0	0	0	2	n.a.
Bena bicolorana	0	0	0	0	1	1	0	0	0	0	0	0	0	0	0	0	0	0	0	0	0	3	[54, 75]
Meganola strigula	0	0	0	0	1	1	0	0	0	0	0	0	0	0	0	0	0	0	0	0	0	1	[54, 75]
Pseudoips prasinana	0	0	0	0	1	1	0	0	0	0	0	0	0	0	0	0	0	0	0	0	0	2	[54, 75]
Drymonia dodonaea	0	0	0	1	1	0	0	0	0	0	0	0	0	0	0	0	0	0	0	0	0	2	[59, 54]
Drymonia ruficornis	0	0	0	1	1	0	0	0	0	0	0	0	0	0	0	0	0	0	0	0	0	2	[59, 54]
Furcula furcula	0	0	0	1	1	1	0	1	0	0	0	0	0	0	0	0	0	0	0	0	0	2	[59, 54]
Harpyia milhauseri	0	0	0	1	1	0	0	0	0	0	0	0	0	0	0	0	0	0	0	0	0	3	[59, 54]
Phalera bucephala	0	0	0	1	1	1	0	0	0	0	0	0	0	0	0	0	0	0	0	0	0	3	[59, 54]
Pheosia tremula	0	0	0	1	1	0	0	0	0	0	0	0	0	0	0	0	0	0	0	0	0	3	[59, 54]
Pterostoma palpina	0	0	0	1	1	0	0	0	0	0	0	0	0	0	0	0	0	0	0	0	0	3	[59, 54]
Ptilodon capucina	0	0	0	1	1	1	0	0	0	0	0	0	0	0	0	0	0	0	0	0	0	3	[59, 54]
Stauropus fagi	1	0	0	1	1	0	0	0	0	0	0	0	0	0	0	0	0	0	0	0	0	3	[59, 54]
Thaumetopoea pityocampa	1	0	0	1	1	0	0	0	0	0	0	0	0	0	0	0	0	0	0	0	0	2	[59, 54]
Thaumetopoea processionea	0	0	0	1	1	0	0	0	0	0	0	0	0	0	0	0	0	0	0	0	0	2	[59, 54]
Batia lunaris	0	0	0	0	0	0	0	0	0	0	0	0	0	0	0	0	0	0	1	0	0	1	[58]
Epicallima formosella	1	0	0	1	1	0	0	0	0	0	0	0	0	0	0	0	0	0	0	0	0	1	[77, 58]
Hofmannophila pseudospretella	0	0	0	1	1	1	1	0	1	0	0	0	0	0	0	0	0	0	0	0	0	1	[58]
Metalampra italica	0	0	0	1	1	0	0	0	0	0	0	0	0	0	0	0	0	0	0	0	0	1	[78]
Prays fraxinella	0	0	0	1	1	0	0	0	0	0	0	0	0	0	0	0	0	0	0	0	0	1	[79]
Emmelina monodactyla	0	0	0	0	0	1	1	0	0	0	0	0	0	0	0	0	0	0	0	0	0	1	[80, 81]
Acrobasis advenella	0	0	0	1	1	1	1	0	0	0	0	0	0	0	0	0	0	0	0	0	0	1	[82]
Acrobasis repandana	0	0	0	1	1	1	0	0	0	0	0	0	0	0	0	0	0	0	0	0	0	1	[82]
Acrobasis tumidana	0	0	0	1	1	1	1	0	0	0	0	0	0	0	0	0	0	0	0	0	0	1	[82]
Aglossa pinguinalis	0	0	0	0	0	1	0	0	1	0	0	0	0	0	0	0	0	0	0	0	0	2	[82]
Agriphila geniculea	0	0	0	0	0	0	0	0	1	0	1	1	0	0	0	0	0	0	0	0	0	1	[83]
Agriphila inquinatella	0	0	0	0	0	1	0	0	0	0	0	1	0	0	0	0	0	0	0	0	0	1	[83]
Agriphila selasella	0	0	0	1	1	1	0	0	0	0	0	0	0	0	0	0	0	0	0	0	0	2	[83]

MOTU	Pest (1) vector (2)	Parasitoid (1)	*Unknown*	Closed habitats	Woodlands	Semi-open habitats	Hedgerows	Shrublands	Open habitats	Cultivated lands	Meadows	Lawns	Mountainous areas	Screes	Slopes	Urban areas	Lowlands	Mesophilous areas	Ubiquitous	Dry areas	Diurnal or flightless	Largeness (size)	**References**
Agriphila straminella	0	0	0	0	0	0	0	0	1	0	1	1	0	0	0	0	0	0	0	0	0	1	[83]
Agriphila tristella	0	0	0	0	0	0	0	0	1	0	1	1	0	0	0	0	0	0	0	0	0	2	[83]
Anania coronata	0	0	0	1	1	1	1	0	0	0	0	0	0	0	0	0	0	0	0	0	0	1	[83]
Anania hortulata	0	0	0	0	0	1	1	0	0	0	0	0	0	0	0	0	1	0	0	0	0	2	[83]
Aphomia sociella	0	0	0	0	0	0	0	0	0	0	0	0	0	0	0	0	0	0	1	0	0	2	[84, 82]
Calamotropha paludella	0	0	0	0	0	0	0	0	1	0	0	0	0	0	0	0	0	0	0	0	0	2	[83]
Catoptria falsella	0	0	0	1	1	1	0	1	0	0	0	0	0	0	0	0	0	0	0	0	0	1	[85, 83]
Catoptria mytilella	0	0	0	1	1	0	0	0	0	0	0	0	0	0	0	0	0	0	0	0	0	1	[83]
Catoptria permutatellus	0	0	0	0	1	1	0	0	0	0	0	0	0	0	0	0	0	0	0	0	0	2	[83]
Catoptria verellus	0	0	0	1	1	0	0	0	0	0	0	0	0	0	0	0	0	0	0	0	0	1	[83]
Chrysoteuchia culmella	0	0	0	0	0	0	0	0	1	0	1	1	0	0	0	0	0	0	0	0	0	1	[83]
Crambus perlella	0	0	0	0	0	1	0	0	1	0	1	1	0	0	0	0	0	0	0	0	0	1	[83]
Crambus pratella	1	0	0	0	0	0	0	0	1	0	1	0	0	0	0	0	0	0	0	1	0	1	[83]
Cydalima perspectalis	0	0	0	1	1	1	0	0	1	1	0	0	0	0	0	1	0	0	0	0	0	2	[83, 86]
Dioryctria abietella	0	0	0	1	1	0	0	0	0	0	0	0	0	0	0	0	0	0	0	0	0	2	[82]
Duponchelia fovealis	0	0	0	0	0	1	0	1	0	0	0	0	0	0	0	0	0	0	0	0	0	1	[83]
Endotricha flammealis	1	0	0	1	1	1	1	0	0	0	0	0	0	0	0	0	0	0	0	0	0	1	[82]
Eudonia delunella	0	0	0	1	1	1	0	0	0	0	0	0	0	0	0	0	0	0	0	0	0	1	[87, 88]
Eudonia lacustrata	0	0	0	1	1	1	0	0	1	0	0	0	0	0	0	0	0	0	0	0	0	1	[87, 88]
Eudonia mercurella	0	0	0	1	1	0	0	0	1	0	0	0	0	0	0	0	0	0	0	0	0	1	[87, 88]
Galleria mellonella	0	0	0	0	0	0	0	0	0	0	0	0	0	0	0	0	0	0	1	0	0	1	[84, 82]
Homoeosoma sinuella	0	0	0	0	0	0	0	0	1	0	0	0	0	0	1	0	0	0	0	0	0	1	[82]
Hypsopygia costalis	0	0	0	1	1	1	1	0	0	0	0	0	0	0	0	0	0	0	0	0	0	1	[82]
Nephopterix angustella	0	0	0	0	0	1	1	0	0	0	0	0	0	0	0	0	0	0	0	0	0	1	[82]
Nomophila noctuella	0	0	0	0	0	0	0	0	1	0	0	0	0	0	0	0	0	0	0	0	0	2	[83]
Oncocera semirubella	0	0	0	0	0	0	0	0	1	0	0	0	0	0	1	0	0	0	0	0	0	1	[82]
Ostrinia nubilalis	0	0	0	1	1	1	0	0	0	0	0	0	0	0	0	1	0	0	0	0	0	2	[83]
Paracorsia repandalis	0	0	0	0	0	1	0	0	1	0	0	0	0	0	1	0	0	0	0	0	0	1	[83]
Pediasia contaminella	0	0	0	0	0	0	0	0	1	0	0	0	0	0	0	0	0	0	0	1	0	2	[83]
Phycita roborella	0	0	0	1	1	1	0	0	0	0	0	0	0	0	0	0	0	0	0	0	0	1	[82]
Phycitodes inquinatella	0	0	0	0	0	0	0	0	0	0	0	0	0	0	0	0	0	0	1	0	0	1	[82]
Pleuroptya ruralis	1	0	0	1	1	1	0	0	1	0	1	0	0	0	0	0	0	0	0	0	0	2	[83]
Pyralis farinalis	0	0	0	0	0	0	0	0	0	0	0	0	0	0	0	1	0	0	0	0	0	1	[82]
Pyrausta despicata	0	0	0	1	1	1	0	0	1	0	1	0	0	0	0	1	0	0	0	0	0	1	[83]
Pyrausta purpuralis	0	0	0	1	1	1	0	0	1	0	1	0	0	0	0	0	0	0	0	0	0	1	[83]
Sciota rhenella	0	0	0	0	0	0	0	0	1	0	0	0	0	0	1	0	0	0	0	0	0	1	[82]
Scoparia ambigualis/basistrigalis	0	0	0	1	1	1	0	0	0	0	0	0	0	0	0	0	0	0	0	0	0	1	[83]
Scoparia conicella	0	0	0	1	1	0	0	0	0	0	0	0	0	0	0	1	0	0	0	0	0	1	[83]
Scoparia pyralella	0	0	0	0	0	1	0	0	1	0	0	0	0	0	0	0	0	0	0	0	0	1	[87]
Selagia spadicella	0	0	0	0	0	0	0	0	1	0	0	1	0	0	1	0	0	0	0	1	0	2	[82]
Sitochroa palealis	0	0	0	0	0	1	0	0	0	0	0	0	0	0	0	0	0	0	0	0	0	2	[83]
Sitochroa verticalis	0	0	0	1	1	0	0	0	1	0	1	0	0	0	0	0	0	0	0	0	0	2	[83]
Synaphe punctalis	0	0	0	0	0	0	0	0	1	0	0	0	0	0	1	0	0	0	0	1	0	1	[82]
Udea ferrugalis	0	0	0	0	0	0	0	0	0	0	0	0	0	0	0	0	0	0	1	0	0	1	[83]
Pyralidae sp. 1	0	0	1	0	0	0	0	0	0	0	0	0	0	0	0	0	0	0	0	0	0	1	n.a.
Pyralidae sp. 2	0	0	1	0	0	0	0	0	0	0	0	0	0	0	0	0	0	0	0	0	0	1	n.a.
Pyralidae sp. 3	0	0	1	0	0	0	0	0	0	0	0	0	0	0	0	0	0	0	0	0	0	1	n.a.
Pyralidae sp. 4	0	0	1	0	0	0	0	0	0	0	0	0	0	0	0	0	0	0	0	0	0	1	n.a.
Deilephila porcellus	0	0	0	0	1	1	0	0	1	0	1	0	0	0	1	0	0	0	0	0	0	3	[59, 54]
Hyloicus pinastri	0	0	0	1	1	0	0	0	0	0	0	0	0	0	0	0	0	0	0	0	0	3	[59, 54]
Laothoe populi	0	0	0	1	1	1	1	0	0	0	0	0	0	0	0	0	0	0	0	0	0	3	[59, 54]
Macroglossum stellatarum	0	0	0	0	0	1	1	0	0	0	1	0	0	0	0	0	0	0	0	0	0	3	[59, 54]
Mimas tiliae	0	0	0	1	1	0	0	0	0	0	0	0	0	0	0	0	0	0	0	0	0	3	[59, 54]
Montescardia tessulatellus	1	0	0	1	1	0	0	0	0	0	0	0	0	0	0	0	0	0	0	0	0	2	[89, 90]
Nemapogon granella	1	0	0	1	1	0	0	0	0	0	0	0	0	0	0	0	0	0	0	0	0	1	[91, 92, 90]
Acleris cristana	0	0	0	1	1	1	0	0	0	0	0	0	0	0	0	0	0	0	0	0	0	1	[93, 94]

MOTU	Pest (1) vector (2)	Parasitoid (1)	*Unknown*	Closed habitats	Woodlands	Semi-open habitats	Hedgerows	Shrublands	Open habitats	Cultivated lands	Meadows	Lawns	Mountainous areas	Screes	Slopes	Urban areas	Lowlands	Mesophilous areas	Ubiquitous	Dry areas	Diurnal or flightless	Largeness (size)	**References**
Acleris hastiana	1	0	0	0	0	1	0	1	0	0	0	0	0	0	0	0	0	0	0	0	0	1	[93, 95, 94]
Ancylis achatana	0	0	0	0	0	1	0	0	0	0	0	0	0	0	0	0	0	0	0	0	0	1	[96]
Archips crataegana	0	0	0	1	1	1	0	0	0	0	0	0	0	0	0	0	0	0	0	0	0	1	[94]
Archips oporana	1	0	0	1	1	0	0	0	0	0	0	0	0	0	0	0	0	0	0	0	0	1	[94]
Archips rosana	0	0	0	0	0	1	0	0	0	0	0	0	0	0	0	0	0	0	0	0	0	1	[93, 94]
Archips xylosteana	1	0	0	1	1	1	0	0	0	0	0	0	0	0	0	0	0	0	0	0	0	1	[94]
Celypha rivulana	0	0	0	0	0	1	0	1	0	0	0	0	0	0	0	0	0	0	0	0	0	1	[96]
Celypha striana	0	0	0	1	1	1	0	0	1	0	0	0	0	0	0	0	0	0	0	0	0	1	[96, 63]
Cnephasia asseclana	0	0	0	0	0	0	0	0	0	0	0	0	0	0	0	0	0	0	1	0	0	1	[94]
Cnephasia communana	0	0	0	0	0	0	0	0	1	1	0	0	0	0	0	0	0	0	0	0	0	1	[93, 94]
Cnephasia cupressivorana	1	0	0	0	0	0	0	0	1	0	1	0	0	0	0	0	0	0	0	0	0	1	[94]
Cnephasia incertana	1	0	0	0	0	1	0	0	1	1	0	0	0	0	0	0	0	0	0	0	0	1	[93, 94]
Cnephasia pasiuana	0	0	0	0	0	0	0	0	1	1	0	0	0	0	0	0	0	0	0	0	0	1	[94]
Cnephasia stephensiana	1	0	0	1	1	1	0	0	1	0	1	1	0	0	0	0	0	0	0	1	0	1	[95, 94]
Cydia fagiglandana	1	0	0	1	1	0	0	0	0	0	0	0	0	0	0	0	0	0	0	0	0	1	[96]
Cydia pomonella	1	0	0	0	0	1	0	0	0	0	0	0	0	0	0	0	0	0	0	0	0	1	[96]
Cydia splendana	0	0	0	1	1	0	0	0	0	0	0	0	0	0	0	0	0	0	0	0	0	1	[96]
Epinotia tenerana	1	0	0	1	1	0	0	0	0	0	0	0	0	0	0	0	0	0	0	0	0	1	[96]
Eudemis porphyrana	0	0	0	0	0	1	0	0	0	0	0	0	0	0	0	0	0	0	0	0	0	1	[96]
Eudemis profundana	0	0	0	1	1	0	0	0	0	0	0	0	0	0	0	0	0	0	0	0	0	1	[96]
Gypsonoma dealbana	1	0	0	1	1	0	0	0	0	0	0	0	0	0	0	0	0	0	0	0	0	1	[96]
Hedya nubiferana	1	0	0	0	0	1	0	0	0	0	0	0	0	0	0	0	0	0	0	0	0	1	[96]
Pandemis cerasana	1	0	0	1	1	1	0	0	0	0	0	0	0	0	0	0	0	0	0	0	0	1	[94]
Rhyacionia buoliana	0	0	0	1	1	0	0	0	0	0	0	0	0	0	0	0	0	0	0	0	0	1	[96]
Tortrix viridana	0	0	0	1	1	0	0	0	0	0	0	0	0	0	0	0	0	0	0	0	0	1	[94]
Zeiraphera griseana	0	0	0	1	1	0	0	0	0	0	0	0	0	0	0	0	0	0	0	0	0	1	[96]
Zeiraphera isertana	0	0	0	1	1	0	0	0	0	0	0	0	0	0	0	0	0	0	0	0	0	1	[96]
Yponomeuta sp.	0	0	0	0	0	0	0	0	0	0	0	0	0	0	0	0	0	0	1	0	0	1	[79]
Ypsolopha parenthesella	0	0	0	1	1	0	0	0	0	0	0	0	0	0	0	0	0	0	0	0	0	1	[79]
Ypsolopha ustella	0	0	0	1	1	0	0	0	0	0	0	0	0	0	0	0	0	0	0	0	0	1	[79]
Lepidoptera sp. 1	0	0	1	0	0	0	0	0	0	0	0	0	0	0	0	0	0	0	0	0	0	2	n.a.
Lepidoptera sp. 2	0	0	1	0	0	0	0	0	0	0	0	0	0	0	0	0	0	0	0	0	0	2	n.a.
Lepidoptera sp. 3	0	0	1	0	0	0	0	0	0	0	0	0	0	0	0	0	0	0	0	0	0	2	n.a.
Lepidoptera sp. 4	0	0	1	0	0	0	0	0	0	0	0	0	0	0	0	0	0	0	0	0	0	2	n.a.
Lepidoptera sp. 5	0	0	1	0	0	0	0	0	0	0	0	0	0	0	0	0	0	0	0	0	0	2	n.a.
Lepidoptera sp. 6	0	0	1	0	0	0	0	0	0	0	0	0	0	0	0	0	0	0	0	0	0	2	n.a.
Lepidoptera sp. 7	0	0	1	0	0	0	0	0	0	0	0	0	0	0	0	0	0	0	0	0	0	2	n.a.
Lepidoptera sp. 8	0	0	1	0	0	0	0	0	0	0	0	0	0	0	0	0	0	0	0	0	0	2	n.a.
Lepidoptera sp. 9	0	0	1	0	0	0	0	0	0	0	0	0	0	0	0	0	0	0	0	0	0	2	n.a.
Lepidoptera sp. 10	0	0	1	0	0	0	0	0	0	0	0	0	0	0	0	0	0	0	0	0	0	2	n.a.
Lepidoptera sp. 11	0	0	1	0	0	0	0	0	0	0	0	0	0	0	0	0	0	0	0	0	0	2	n.a.
Lepidoptera sp. 12	1	0	1	0	0	0	0	0	0	0	0	0	0	0	0	0	0	0	0	0	0	2	n.a.
Panorpa germanica	0	0	0	1	1	1	1	0	1	0	1	0	0	0	0	0	0	0	0	0	0	1	n.a.
Chrysoperla carnea	0	0	0	1	1	0	0	0	0	0	0	0	0	0	0	0	0	0	0	0	0	1	[97]
Chrysotropia ciliata	0	0	0	1	1	0	0	0	0	0	0	0	0	0	0	0	0	0	0	0	0	1	[97]
Nineta flava	0	0	0	1	1	0	0	0	0	0	0	0	0	0	0	0	0	0	0	0	0	1	[97]
Nothochrysa capitata	0	0	0	1	1	0	0	0	0	0	0	0	0	0	0	0	0	0	0	0	0	1	[97]
Nothochrysa fulviceps	0	0	0	1	1	0	0	0	0	0	0	0	0	0	0	0	0	0	0	0	0	1	[97]
Peyerimhoffina gracilis	0	0	0	1	1	0	0	0	0	0	0	0	0	0	0	0	0	0	0	0	0	1	[97]
Pseudomallada flavifrons	0	0	0	1	1	0	0	0	0	0	0	0	0	0	0	0	0	0	0	0	0	1	[97]
Pseudomallada prasinus	0	0	0	1	1	0	0	0	0	0	0	0	0	0	0	0	0	0	0	0	0	1	[97]
Chrysopidae sp.	0	0	0	1	1	0	0	0	0	0	0	0	0	0	0	0	0	0	0	0	0	1	[97]
Drepanepteryx phalaenoides	0	0	0	1	1	0	0	0	0	0	0	0	0	0	0	0	0	0	0	0	0	2	[97]
Hemerobius humulinus	0	0	0	1	1	0	0	0	0	0	0	0	0	0	0	0	0	0	0	0	0	1	[97]
Hemerobius micans	0	0	0	1	1	0	0	0	0	0	0	0	0	0	0	0	0	0	0	0	0	1	[97]
Hemerobius pini	0	0	0	1	1	0	0	0	0	0	0	0	0	0	0	0	0	0	0	0	0	1	[97]

MOTU	Pest (1) vector (2)	Parasitoid (1)	*Unknown*	Closed habitats	Woodlands	Semi-open habitats	Hedgerows	Shrublands	Open habitats	Cultivated lands	Meadows	Lawns	Mountainous areas	Screes	Slopes	Urban areas	Lowlands	Mesophilous areas	Ubiquitous	Dry areas	Diurnal or flightless	Largeness (size)	**References**
Distoleon tetragrammicus	0	0	0	1	1	0	0	0	0	0	0	0	0	0	0	0	0	0	0	0	0	2	[97]
Chorthippus parallelus	0	0	0	0	0	0	0	0	1	0	1	0	0	0	0	0	0	0	0	0	1	3	[98]
Leptophyes punctatissima	0	0	0	0	0	1	0	1	0	0	0	0	0	0	0	0	0	0	0	0	1	3	[98]
Meconema thalassinum	0	0	0	0	0	1	1	0	0	0	0	0	0	0	0	0	0	0	0	0	1	3	[98]
Caecilius fuscopterus	0	0	0	1	1	0	0	0	0	0	0	0	0	0	0	0	1	0	0	0	1	1	[99]
Valenzuela flavidus	0	0	0	1	1	1	0	1	0	0	0	0	0	0	0	0	0	0	0	0	1	1	[99]
Loensia pearmani	0	0	0	1	1	0	0	0	0	0	0	0	0	0	0	0	0	0	0	0	1	1	[100]
Metylophorus nebulosus	0	0	0	1	1	1	0	1	0	0	0	0	0	0	0	0	0	0	0	0	1	1	[100]
Psococerastis gibbosa	0	0	0	1	1	0	0	0	0	0	0	0	0	0	0	0	0	0	0	0	1	1	[100]
Subilla confinis	0	0	0	1	1	0	0	0	0	0	0	0	0	0	0	0	0	0	0	0	0	1	[101]
Hydropsyche instabilis	0	0	0	0	0	0	0	0	1	0	0	0	0	0	0	0	0	0	0	0	0	1	[102]
Hydropsyche pellucidula	0	0	0	0	0	0	0	0	1	0	0	0	0	0	0	0	0	0	0	0	0	1	[102]
Ceraclea annulicornis	0	0	0	0	0	0	0	0	1	0	0	0	0	0	0	0	0	0	0	0	0	1	[102]
Stenophylax mitis	0	0	0	0	0	0	0	0	1	0	0	0	0	0	0	0	0	0	0	0	0	1	[102]
Plectrocnemia conspersa	0	0	0	0	0	0	0	0	1	0	0	0	0	0	0	0	0	0	0	0	0	1	[102]
Rhyacophila dorsalis	0	0	0	0	0	0	0	0	1	0	0	0	0	0	0	0	0	0	0	0	0	1	[102]
Oniscus asellus	0	0	0	1	1	0	0	0	0	0	0	0	0	0	0	0	0	0	0	0	1	3	[103]
Porcellio scaber	0	0	0	0	0	0	0	0	0	0	0	0	0	0	0	0	0	0	0	0	1	3	[103]
Porcellio spinicornis	0	0	0	0	0	0	0	0	0	0	0	0	0	0	0	0	0	0	0	1	1	3	[103]
Isopoda sp.	0	0	1	0	0	0	0	0	0	0	0	0	0	0	0	0	0	0	0	0	1	3	n.a.

1. British Arachnological Society 2018. Spider and Harvestman Recording Scheme website. http://srs.britishspiders.org.uk/
2. Nentwig, W., Blick, T., Gloor, D., Hänggi, A. & Kropf, C. 2017. Spiders of Europe. *University of Bern*, https://araneae.unibe.ch/
3. Fjellberg, A. 2007. Entomobryomorpha and Symphypleona. Fauna Entomologica Scandinavica, vol. 42, *E.J. Brill/Scandinavian Science Press, Leiden*, 264 pp.
4. Harz, K. 1957. Die Geradflügler Mitteleuropas. *Gustav Fischer, Jena*, 494 pp.
5. Jeannel, R. 1942. Coléoptères Carabiques. Deuxième partie. Faune de France, vol. 40, *Paris*, 1163 pp.
6. Jeannel, R. 1941. Coléoptères Carabiques. Première partie. Faune de France, vol. 39, *Paris*, 571 pp.
7. Bílý, S. 1989. Longhorn beetles (Coleoptera, Cerambycidae) of Fennoscandia and Denmark. Fauna Entomologica Scandinavica, vol. 22, *E.J. Brill/Scandinavian Science Press, Leiden*, 203 pp.
8. du Chatenet, G. 2000. Coléoptères phytophages d'Europe. *N.A.P., Vitry-sur-Seine*, 359 pp.
9. Hoffmann, A. 1954. Coléoptères Curculionides. Deuxième partie. Faune de France, vol. 59, *Paris*, 1208 pp.
10. Wallace, J. 2009. Elateridae of the British Isles. http://elateridae.co.uk/, accessed on 17 Jan 2019
11. de Laclos, E. 2006. Les Coléoptères associés aux scolytes du chêne en forêt domaniale de Cîteaux (département de la Côte-d'Or). *Bulletin mensuel de la Société linnéenne de Lyon* 75: 67–82.
12. Constantin, R. & Liberti, G. 2011. Coléoptères Dasytidae de France. *Musée des confluences, Centre de Conservation et d'Étude des Collections, Lyon*, 143 pp.
13. Gambrell, F. L. 1946. The European Chafer *Amphimallon majalis* and its control in lawns. *Journal of Economic Entomology* 39: 168–173. doi: 10.1093/jee/39.2.168.
14. Gryisco, G., Whitecomb, W., Burrage, R., Logothetis, C. & Schwardt, H. 1954. Biology of the European chafer *Amphimallon majalis* Razoumowsky (Scarabaeidae). *Memoirs. Cornell University Agricultural Experiment Station* 228: 1–35.
15. Barbalat, S. 1998. Importance of forest structures on four beetle families (Col.: Buprestidae, Cerambycidae, Lucanidae and phytophagous Scarabaeidae) in the Areuse Gorges (Neuchatel, Switzerland). *Revue suisse de Zoologie* 105: 569–580. doi: 10.5962/bhl.part.80051.
16. Alford, D. V. 2018. Pests of ornamental trees, shrubs and flowers. *Taylor & Francis Group,* 2nd ed., 480 pp.
17. Andrzej, M., Boháč, J. & Matějíček, J. 2015. Occurrence of species of the genus *Eusphalerum* Kr. (Col., Staphylinidae, Omaliinae) in the Giant Mountains area, *In:* Štursa, J., Mazurski, K. R., Palucki, A. & Potocka, J. (ed). Geoekologické problémy Krkonoš, *Opera Corcontica*, vol. 41, 287–300 pp. doi: 10.13140/RG.2.1.2094.0002.
18. Albouy, V. & Caussanel, C. 1990. Dermaptères ou Perce-oreilles. Faune de France, vol. 75, *Paris*, 245 pp.
19. Barnard, P. C. 2011. The Royal Entomological Society Book of British Insects. *Wiley-Blackwell*, 600 pp.
20. Ellis, W. N. 2018. Plant Parasites of Europe - leafminers, galls and fungi. https://bladmineerders.nl/, accessed on 17 Jan 2019
21. Faval, A., Fèvre, E., Coutin, R., Minost, C. & Laporte, V. 2018. Encyclopédie des ravageurs européens. http://www7.inra.fr/hyppz/, accessed on 17 Jan 2019
22. Grzywacz, A. & Jankowski, A. 2014. The report of *Hydrophoria ruralis* (Meigen, 1826) (Diptera: Anthomyiidae) in Poland. *Dipteron* 30: 19–23.
23. Séguy, E. 1923. Diptères Anthomyides. Faune de France, vol. 6, *Paris*, 393 pp.
24. Séguy, E. 1940. Diptères Nématocères (Fungivoridae, Lycoriidae, Hesperinidae, Bibionidae, Scatopsidae, Phrynidae, Pachyneuridae, Blepharoceridae). Faune de France, vol. 36, *Paris*, 365 pp.
25. Rognes, K. 1998. Family Calliphoridae, *In:* Papp, L. & Darvas, B. (ed). Volume 3: Higher Brachycera. Contributions to a manual of Palaearctic Diptera, *Science Herald, Budapest*, 880 pp.
26. Falk, S. 2016. Draft key to British Calliphoridae and Rhinophoridae. 86 pp. http://www.stevenfalk.co.uk/files/21577/testkeytobritishblowflies132016.pdf (accessed on 17 Jan 2019).
27. Luhken, R., Kiel, E., Steinke, S. & Fladung, R. 2015. Topsoil conditions correlate with the emergence rates of *Culicoides chiopterus* and *Culicoides dewulfi* (Diptera: Ceratopogonidae) from cowpats. *Parasitology Research* 114: 1113–1117. doi: 10.1007/s00436-014-4284-z.
28. Lods-Crozet, B. 1992. Étude des larves de Chironomidae (Diptera) du Léman : 1. systématique et faunistique *Mitteilungen der Schweizerischen Entomologischen Gesellschaft* 65: 5–23. doi: 10.5169/seals-402467.

29. Moller Pillot, H. K. M. 2014. Biology and Ecology of the Aquatic Orthocladiinae. Chironomidae Larvae of the Netherlands and Adjacent Lowlands, vol. 3, *Brill, Leiden*, 316 pp.
30. Cranston, P. 2010. Chironomid Taxa. http://chirokey.skullisland.info/, accessed on 17 Jan 2019
31. Séguy, E. 1934. Diptères (Brachycères) (Muscidae Acalypterae et Scatophagidae). Faune de France, vol. 28, *Paris*, 832 pp.
32. Chvála, M. 1994. The Empidoidea (Diptera) of Fennoscandia and Denmark III: genus *Empis*. Fauna Entomologica Scandinavica, vol. 29, *E.J. Brill/Scandinavian Science Press, Leiden*, 192 pp.
33. Niesiołowski, S. 2006. Morphology, biology, phenology and occurrence of the genus *Empis* Linnaeus (Empididae, Diptera) in Poland. *Fragmenta Faunistica* 49: 1–39.
34. Lindsey, J. K. 2011. Ecology of Commanster. http://www.commanster.eu/commanster/Insects, accessed on 4 Jul 2017
35. Helyer, N., Cattlin, N. D. & Brown, K. C. 2014. Biological control in plant protection: A colour handbook, Second edition. *CRC Press*, 276 pp.
36. Fränzle, O., Kappen, L., Blume, H.-P. & Dierssen, K. 2008. Ecosystem organization of a complex landscape : long-term research in the Bornhöved Lake District, Germany. Ecological studies, vol. 202, *Springer, Berlin*, 391 pp.
37. Pont, A. C. 2000. Family Fanniidae, *In:* Papp, L. & Darvas, B. (ed). Appendix. Contributions to a manual of Palaearctic Diptera, *Science Herald, Budapest*, 604 pp.
38. Hwang, C. & Turner, B. D. 2005. Spatial and temporal variability of necrophagous Diptera from urban to rural areas. *Medical and Veterinary Entomology* 19: 379–391. doi: 10.1111/j.1365-2915.2005.00583.x.
39. Pierre, C. 1924. Diptères : Tipulidae. Faune de France, vol. 8, *Paris*, 159 pp.
40. Brinkmann, R. 1991. Zur Habitatpräferenz und Phänologie der Limoniidae, Tipulidae und Cylindrotomidae (Diptera) im Bereich eines norddeutschen Tieflandbaches. *Faunistisch-ökologische Mitteilungen / Supplement* 11: 1–156.
41. Grzywacz, A. & Pape, T. 2014. Larval morphology of *Atherigona orientalis* (Schiner) (Diptera: Muscidae) —A species of sanitary and forensic importance. *Acta Tropica* 137: 174–184. doi: 10.1016/j.actatropica.2014.05.018.
42. Cuny, R. 1978. Muscidae und Calliphoridae (Insecta: Diptera) der Lägern (Schweiz: Jura). *Mitteilungen der Schweizerischen Entomologischen Gesellschaft* 51: 377–394.
43. Økland, B., Götmark, F. & Nordén, B. 2008. Oak woodland restoration: testing the effects on biodiversity of mycetophilids in southern Sweden. *Biodiversity and Conservation* 17: 2599. doi: 10.1007/s10531-008-9325-4.
44. Pape, T. 1987. The Sarcophagidae (Diptera) of Fennoscandia and Denmark. Fauna Entomologica Scandinavica, vol. 19, *E.J. Brill/Scandinavian Science Press, Leiden*, 203 pp.
45. Drees, M. 2014. Die Fleischfliegen des Hagener Raumes (Diptera: Sarcophagidae). *Entomologische Zeitschrift, Stuttgart* 124: 169–181.
46. Heller, K. 1999. Trauermucken (Diptera: Sciaridae) von Gonnersdorf (Kr. Daun). Beitrage zur Insektenfauna der Eifeldorfer 20. *Dendrocopos* 26: 249–262.
47. Séguy, E. 1926. Diptères (Brachycères) (Stratiomyiidae, Erinnidae, Coenomyiidae, Rhagionidae, Tabanidae, Codidae, Nemestrinidae, Mydaidae, Bombyliidae, Therevidae, Omphralidae). Faune de France, vol. 13, *Paris*, 308 pp.
48. Speight, M. C. D. 2014. Species accounts of European Syrphidae (Diptera) 2014. Syrph the Net, the database of European Syrphidae, vol. 78, *Dublin*, 321 pp.
49. Tschorsnig, H.-P. & Herting, B. 1994. The Tachinids (Diptera: Tachinidae) of Central Europe: identification keys for the species and data on distribution and ecology. Translated by Rotraud Rayner & Chris Raper, 2001. *State Museum of Natural Science, Stuttgart*.
50. Pohjoismäki, J. 2013. Observations of Tachinidae (Diptera) in the surroundings of Friedberg (Hessen, Germany) with notes on some interesting species. *Tachinid Times* 26: 39–45.
51. Andersen, S. 1996. The Siphonini (Diptera: Tachinidae) of Europe. Fauna Entomologica Scandinavica, vol. 33, *E.J. Brill/Scandinavian Science Press, Leiden*, 148 pp.
52. Ossiannilsson, F. 1981. The Auchenorrhyncha (Homoptera) of Fennoscandia and Denmark, Volume 2: Families Cicadidae, Cercopidae, Membracidae and Cicadellidae (Excl. Deltocephalinae). Fauna Entomologica Scandinavica, vol. 7, part 2, *E.J. Brill/Scandinavian Science Press, Leiden*, 222 pp.
53. Wagner, E. & Weber, H. H. 1964. Hétéroptères Miridae. Faune de France, vol. 67, *Paris*, 589 pp.
54. Robineau, R., Bachelard, P., Bérard, R., Colomb, C., Demerges, D., Doux, Y., Fournier, F., Gibeaux, C., Maechler, J., Schmit, P. & Tautel, C. 2007. Guide des papillons nocturnes de France. *Delachaux et Niestlé, Paris*, 287 pp.
55. Berland, L. 1947. Hyménoptères Tenthredoïdes. Faune de France, vol. 47, *Paris*, 496 pp.
56. Buck, M., Marshall, S. A. & Cheung, D. K. B. 2008. Identification Atlas of the Vespidae (Hymenoptera, Aculeata) of the northeastern Nearctic region. *Canadian Journal of Arthropod Identification*. doi: 10.3752/cjai.2008.05.
57. Heath, J. 1976. Volume 1: Micropterigidae to Heliozelidae. The moths and butterflies of Great Britain and Ireland, *Harley Books, London*, 343 pp.
58. Emmet, A. M. & Langmaid, J. R. 2002. Volume 4 (Part 1): Oecophoridae to Scythrididae (excluding Gelechiidae). The moths and butterflies of Great Britain and Ireland, *Harley Books, London*, 326 pp.
59. Leraut, P. 2006. Volume 1: Bombyx, Sphynx, Écailles. Papillons de nuit d'Europe, *NAP Editions, Verrières le Buisson*, 387 pp.
60. Ronkay, L. & Fibiger, M. 2003. Catocalinae & Plusiinae. Noctuidae Europaeae, vol. 10, *Entomological Press, Sorø*, 452 pp.
61. Fibiger, M. 2010. Rivulinae, Boletobiinae, Hypenodinae, Araeopteroninae, Eublemminae, Herminiinae, Hypeninae, Phytometrinae, Euteliinae, and Micronoctuidae, including Supplement to volumes 1–11. Noctuidae Europaeae, vol. 12, *Entomological Press, Sorø*, 451 pp.
62. Kimber, I. 2018. UKMoths. https://www.ukmoths.org.uk/, accessed on Aug. 3rd 2018
63. Manley, C. 2015. British Moths: second Edition. A Photographic Guide to the Moths of Britain and Ireland. *Bloomsbury Publishing*, 448 pp.
64. Elsner, G., Huemer, P. & Tokár, Z. 1999. Die Palpenmotten (Lepidoptera, Gelechiidae) Mitteleuropas : Bestimmung, Verbreitung, Flugstandort, Lebensweise der Raupen. *František Slamka, Bratislava*, 208 pp.
65. Huemer, P. 1999. Gelechiidae I (Gelechiinae: Teleiodini, Gelechiini). Microlepidoptera of Europe, vol. 3, *Apollo Books, Stenstrup*, 356 pp.
66. Leraut, P. 2009. Volume 2: Géomètres. Papillons de nuit d'Europe, *NAP Editions, Verrières le Buisson*, 795 pp.
67. Rézbányai-Reser, L. 1986. *Gnophos italohelveticus* sp. nova, eine bisher übersehene Spannerart aus den Südalpen, und über den *crenulatus*-Formenkreis Südwesteuropas (Lepidoptera, Geometridae). *Nota Lepidopterologica* 9: 99–142.
68. Fibiger, M. & Hacker, H. 2007. Amphipyrinae, Condicinae, Eriopinae, Xyleninae (part). Noctuidae Europaeae, vol. 9, *Entomological Press, Sorø*, 410 pp.
69. Fibiger, M. 1990. Noctuinae I. Noctuidae Europaeae, vol. 1, *Entomological Press, Sorø*, 208 pp.
70. Ronkay, L., Yela, J. L. & Hreblay, M. 2001. Hadeninae II. Noctuidae Europaeae, vol. 5, *Entomological Press, Sorø*, 452 pp.
71. Zilli, A., Ronkay, L. & Fibiger, M. 2005. Apameini. Noctuidae Europaeae, vol. 8, *Entomological Press, Sorø*, 323 pp.
72. Fibiger, M. 1993. Noctuinae II. Noctuidae Europaeae, vol. 2, *Entomological Press, Sorø*, 230 pp.
73. Hacker, H., Ronkay, L. & Hreblay, M. 2002. Hadeninae I. Noctuidae Europaeae, vol. 4, *Entomological Press, Sorø*, 419 pp.
74. Ronkay, G. & Ronkay, L. 1995. Cuculliinae II. Noctuidae Europaeae, vol. 7, *Entomological Press, Sorø*, 224 pp.
75. Ronkay, L., Steiner, A., Zilli, A. & Fibiger, M. 2009. Pantheinae, Dilobinae, Acronictinae, Eustrotiinae, Nolinae, Bagisarinae,

Acontiinae, Metoponiinae, Heliothinae, and Bryophilinae. Noctuidae Europaeae, vol. 11, *Entomological Press, Sorø*, 504 pp.
76. Ronkay, G. & Ronkay, L. 1994. Cuculliinae I. Noctuidae Europaeae, vol. 6, *Entomological Press, Sorø*, 282 pp.
77. Lvovskii, A. 1981. Oecophoridae family. Insect Key for the European part of USSR, vol. 4(2), *Leningrad*.
78. Tokár, Z. 2005. Die Oecophoridae s. l. (Lepidoptera) Mitteleuropas : Bestimmung, Verbreitung, Habitat, Bionomie. *Františ‌ek Slamka, Bratislava*, 120 pp.
79. Emmet, A. M. 1996. Volume 3: Yponomeutidae to Elachistidae. The moths and butterflies of Great Britain and Ireland, *Harley Books, London*, 452 pp.
80. Gielis, C. 1996. Pterophoridae. Microlepidoptera of Europe, vol. 1, *Apollo Books, Stenstrup*, 222 pp.
81. Hoskins, A. 2017. Learn about butterfly, Common Brown Plume moth. http://www.learnaboutbutterflies.com/Britain%20-%20Emmelina%20monodactyla.htm, accessed on 17 Jan 2019
82. Leraut, P. 2014. Volume 4: Pyrales 2. Papillons de nuit d'Europe, *NAP Editions, Verrières le Buisson*, 440 pp.
83. Leraut, P. 2012. Volume 3: Zygènes, Pyrales 1 et Brachodides. Papillons de nuit d'Europe, *NAP Editions, Verrières le Buisson*, 599 pp.
84. Slamka, F. 2006. Pyralinae, Galleriinae, Epipaschiinae, Cathariinae & Odontiinae. Pyraloidea (Lepidoptera) of Europe : identification, distribution, habitat, biology, vol. 1, *František Slamka, Bratislava*, 138 pp.
85. Slamka, F. 2008. Crambinae & Schoenobiinae. Pyraloidea (Lepidoptera) of Europe : identification, distribution, habitat, biology, vol. 2, *František Slamka, Bratislava*, 224 pp.
86. Leuthardt, F. & Kenis, M. 2015. *Cydalima perspectalis* (box tree moth). Invasive Species Compendium https://www.cabi.org/isc/datasheet/118433, accessed on 17 Jan 2019
87. Goater, B., Nuss, M. & Speidel, W. 2005. Pyraloidea I (Crambidae: Acentropinae, Evergestinae, Heliothelinae, Schoenobiinae, Scopariinae). Microlepidoptera of Europe, vol. 4, *Apollo Books, Stenstrup*, 304 pp.
88. Pritchard, T. 2017. The Moths of Suffolk. http://www.suffolkmoths.org.uk, accessed on 17 Jan 2019
89. Jonko, C. 2018. *Montescardia tessulatellus* (Zeller, 1846). Lepidoptera Mundi https://lepidoptera.eu/, accessed on 17 Jan 2019
90. Rennwald, E. & Rodeland, J. 2018. Lepiforum: Bestimmung von Schmetterlingen (Lepidoptera) und ihren Präimaginalstadien. http://www.lepiforum.de, accessed on 17 Jan 2019
91. Heath, J. 1985. Volume 2: Cossidae to Heliodinidae. The moths and butterflies of Great Britain and Ireland, *Harley Books, London*, 460 pp.
92. Gaedike, R. 2015. Tineidae I (Dryadaulinae, Hapsiferinae, Euplocaminae, Scardiinae, Nemapogoninae and Meessiinae). Microlepidoptera of Europe, vol. 7, *Brill, Leiden*, 308 pp.
93. Balachowsky, A. S. 1966. Entomologie appliquée à l'agriculture, Tome II, vol. 1. Lépidoptères ('Hepialoidea', 'Stigmelloidea', 'Incurvarioidea', 'Cossoidea', 'Tineoidea', 'Tortricoidea'). *Masson et Cie., Paris*, 1057 pp.
94. Razowski, J. 2002. Tortricidae (Lepidoptera) of Europe. 1: Tortricinae and Chlidanotinae, *František Slamka, Bratislava* 247 pp.
95. Razowski, J. 2001. Die Tortriciden (Lepidoptera, Tortricidae) Mitteleuropas : Bestimmung, Verbreitung, Flugstandort, Lebensweise der Raupen. *František Slamka, Bratislava*, 319 pp.
96. Razowski, J. 2003. Tortricidae (Lepidoptera) of Europe. 2: Olethreutinae, *František Slamka, Bratislava* 301 pp.
97. Séméria, Y. & Berland, L. 1988. Atlas des névroptères de France et d'Europe : mégaloptères, raphidioptères, névroptères planipennes, mécoptères. *Paris : Société nouvelle des éditions Boubée*, Nouvelle éd. revue et augm., 190 pp.
98. Holst, K. T. 1986. The Saltatoria (bush-crickets, crickets and grasshoppers) of Northern Europe. Fauna Entomologica Scandinavica, vol. 16, *E.J. Brill/Scandinavian Science Press, Leiden*, 127 pp.
99. Lienhard, C. 1998. Psocoptères euro-méditerranéens. Faune de France, vol. 83, *Paris*, 517 pp.
100. Saville, R. & Alexander, K. 2017. National Barkfly Recording Scheme (Britain and Ireland). https://www.brc.ac.uk/schemes/barkfly/homepage.htm, accessed on 17 Jan 2019
101. Chinery, M. 1988. Insectes de France et d'Europe occidentale. *Arthaud, Paris*, 319 pp.
102. Mosely, M. E. 1939. The British caddis flies (Trichoptera): a collector's handbook. *London*, 320 pp.
103. Vandel, A. 1962. Isopodes terrestres. Deuxième partie. Faune de France, vol. 66, *Paris*, 417 pp.

Appendix 3. Predator traits table

Matrix summarizing the taxonomic identity and the habitat composition of the surroundings for the 9 sampled colonies of *Plecotus* bats.

Colony	*P. auritus*	*P. austriacus*	*P. macrobullaris*	Urbanized environment	Wetlands, water	Lawns, isolated trees	Grasslands, prairies	Forest, shrub	Farmland
1. Satigny	1	0	0	12.44	4.09	21.93	5.73	17.31	38.50
2. Butin	1	0	0	19.25	8.98	32.45	2.06	11.43	25.84
3. Choulex	1	0	0	10.46	19.31	24.55	2.92	12.85	29.90
4. Presinge	1	0	0	10.85	3.04	25.61	7.19	21.23	32.09
5. Sappey	1	0	0	3.56	1.50	5.40	29.54	41.69	18.30
6. Collex	0	1	0	9.57	18.98	18.43	5.98	17.34	29.70
7. Hermance	0	1	0	4.33	40.94	11.17	4.15	16.94	22.48
8. Cartigny	0	0	1	6.35	3.54	10.71	6.08	22.91	50.41
9. Léaz	0	0	1	4.31	3.40	5.83	14.62	54.32	17.52

Appendix 4. Prey composition for community samples (one colony for each bat species: Satigny, Collex and Cartigny)

(a) Venn diagram representing the number of shared or specific prey species detected among the three bat species. (b) Weighted proportions of arthropod orders found in the diet of bats throughout the sampled period (Spr: spring; Sum: summer; Aut: autumn).

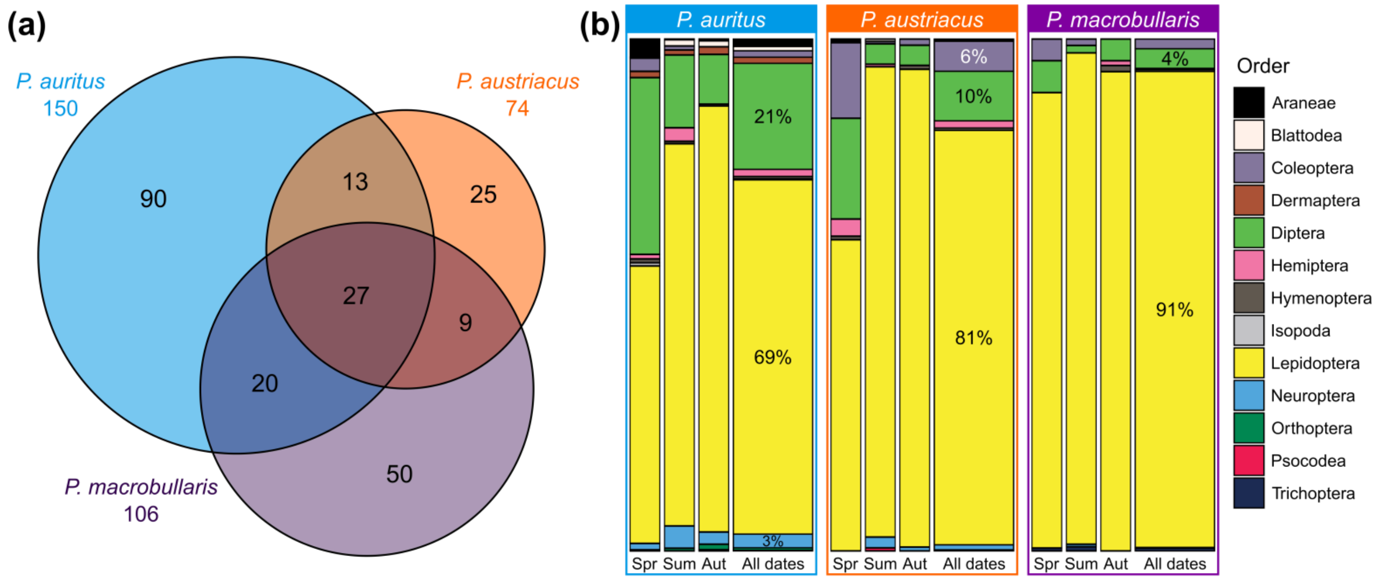

Appendix 5. Principal component analysis of long-eared bats' diet reported in various studies.

The analysis relied on the diet composition of the three long-eared bat species described at the ordinal level (Lepidoptera, Diptera, etc.) as percentage frequency (total=100%, see Vaughan, 1997). Values were transformed following Aitchison (1982) before applying principal component analysis to compositional data. The diet composition as recovered by our approach lie amongst previously published ones. Studies based on the same metabarcoding methodology than ours were excluded.

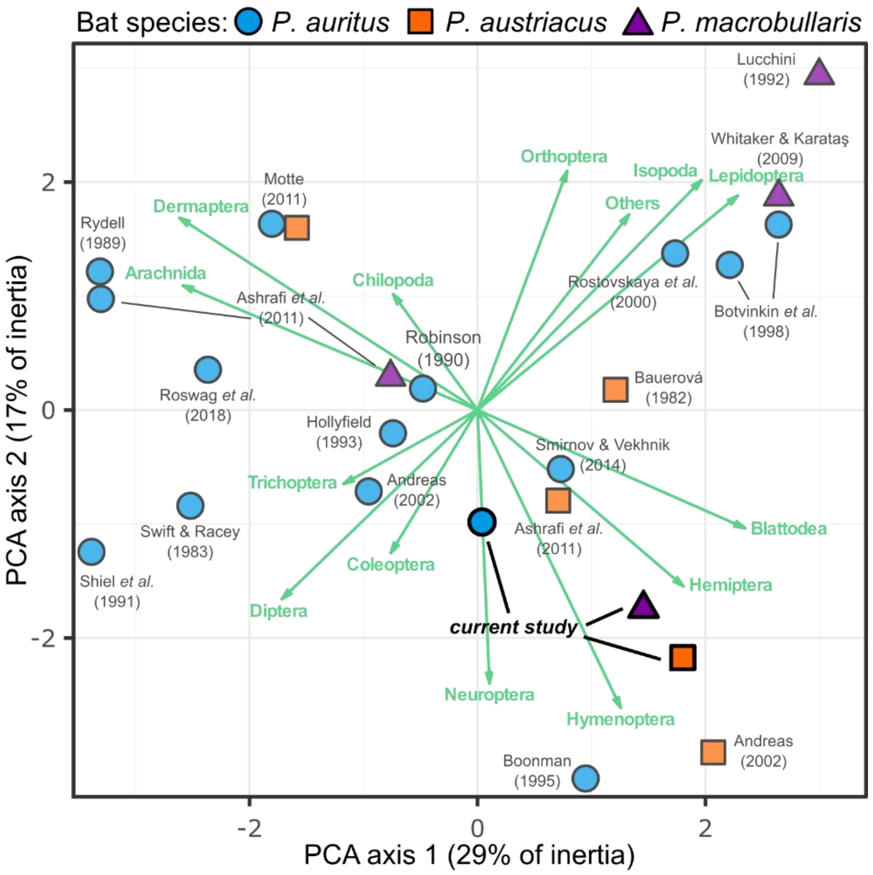

Aitchison, J. 1982. The statistical analysis of compositional data. *Journal of the Royal Statistical Society. Series B (Methodological)*, 44: 139–177. doi: 10.1111/j.2517-6161.1982.tb01195.x

Andreas, M. 2002. Potravní ekologie netopýrů Středomoří [Feeding ecology of bats in the Mediterranean]. PhD Thesis. *Institute of Applied Ecology, Czech Agriculture University*, Prague, 163 pp.

Ashrafi, S., Beck, A., Rutishauser, M., Arlettaz, R. & Bontadina, F. 2011. Trophic niche partitioning of cryptic species of long-eared bats in Switzerland: implications for conservation. European *Journal of Wildlife Research* 57: 843–849. doi: 10.1007/s10344-011-0496-z.

Bauerová, Z. 1982. Contribution to the trophic ecology of the grey long-eared bat, *Plecotus austriacus*. *Folia Zoologica* 31: 113-122.

Boonman, M. 1995. Voedselkeuze grootoorvleermuis. *Zoogdier* 6: 19–22.

Botvinkin, A.D., Shilenkova, Yu. V. & Shilenkov, V.G. 1998. Comparison of the diet of the brown long-eared bat (*Plecotus auritus* L.) in ecologically contrasting habitats of the eastern Siberia. *Plecotus et al.* 1: 27–34.

Hollyfield, A. M. 1993. Diet in relation to prey availability and the directionality and design of echolocation calls in three species of British bats. PhD Thesis. *University of Bristol*.

Lucchini, G. 1992. Une mangeoire de *Plecotus auritus* L. (Mammalia, Chiroptera). Régime alimentaire et comportement. Bachelor Thesis. Université de Neuchâtel, Neuchâtel, 40 pp.

Motte, G. 2011. Étude comparée de l'écologie de deux espèces jumelles de Chiroptères (Mammalia : Chiroptera) en Belgique: l'oreillard roux (*Plecotus auritus*) (Linn., 1758) et l'oreillard gris (*Plecotus austriacus*) (Fischer, 1829). PhD Thesis. *Université de Liège*, 123 pp.

Robinson, M. F. 1990. Prey selection by the brown long–eared bat, *Plecotus auritus*. *Myotis* 28: 5–18.

Rostovskaya, M.S., Zhukova, D.V., Illarionova, A.E., Ustyugova, S.V., Borissenko, A.V. & Sviridov, A.V., 2000. Insect prey of the long-eared bat *Plecotus auritus* (L.) (Chiroptera: Vespertilionidae) in central Russia. *Russian entomological Journal* 9: 185–189.

Roswag, A., Becker, N. I. & Encarnação, J. A. 2018. Isotopic and dietary niches as indicators for resource partitioning in the gleaner bats *Myotis bechsteinii*, *M. nattereri*, and *Plecotus auritus*. *Mammalian Biology* 89: 62–70. doi: 10.1016/j.mambio.2017.12.006.

Rydell, J. 1989. Food habits of northern (*Eptesicus nilssoni*) and brown long-eared (*Plecotus auritus*) bats in Sweden. *Ecography* 12: 16–20. doi: 10.1111/j.1600-0587.1989.tb00817.x.

Shiel, C. B., McAney, C. M. & Fairley, J. S. 1991. Analysis of the diet of Natterer's bat *Myotis nattereri* and the common long-eared bat *Plecotus auritus* in the West of Ireland. *Journal of Zoology* 223: 299–305. doi: 10.1111/j.1469-7998.1991.tb04766.x.

Smirnov, D.G. & Vekhnik, V.P. 2014. Ecology of nutrition and differentiation of the trophic niches of bats (Chiroptera: Vespertilionidae) in floodplain ecosystems of the Samara Bend. *Biology Bulletin* 41: 60–70. doi: 10.1134/S1062359014010105.

Swift, S. M. & Racey, P. A. 1983. Resource partitioning in two species of Vespertilionid bats (Chiroptera) occupying the same roost. *Journal of Zoology* 200: 249–259. doi: 10.1111/j.1469-7998.1983.tb05787.x.

Vaughan, N. 1997. The diets of British bats (Chiroptera). *Mammal Review* 27: 77–94. doi: 10.1111/j.1365-2907.1997.tb00373.x.

Whitaker, J. O. & Karataş, A. 2009. Food and feeding habits of some bats from Turkey. *Acta Chiropterologica* 11: 393–404. doi: 10.3161/150811009X48561.

Appendix 6. Analysis of variance, using residual randomization permutation procedure

For each test, 10’000 permutations were carried. Estimation method: Ordinary Least Squares; Sums of Squares and Cross-products: Type I; Effect sizes (Z) based on F distributions.

1. Dataset consisting of “community replicates”

Factor	Df	SS	MS	R^2	F	Z	*p*-value	Significance
**Bat species**	2	39.05	19.524	0.02086	1.0143	0.27628	0.391361	
**Date**	10	207.20	20.720	0.11071	1.0764	3.13979	0.001300	**
**Bat species*Date**	20	412.57	20.628	0.22044	1.0716	2.36070	0.009199	**
**Residuals**	63	1212.75	19.250	0.64798				
**Total**	95	1871.57						

2. Dataset consisting of “smaller samples”

Factor	Df	SS	MS	R^2	F	Z	*p*-value	Significance
**Bat species**	2	33.61	16.803	0.01530	1.4404	7.2453	9.999e-05	***
**Date**	10	131.21	13.121	0.05973	1.1247	4.9629	9.999e-05	***
**Bat species*Date**	18	223.62	12.423	0.10180	1.0649	3.5054	4.00e-04	***
**Residuals**	155	1808.17	11.666	0.82317				
**Total**	185	2196.60						

Appendix 7. Principal component analysis of seasonal prey composition of diet

Principal component analysis based on the prey composition of fecal samples from different colonies and seasons. Each bat species is represented by a different color and each season by a different shape.

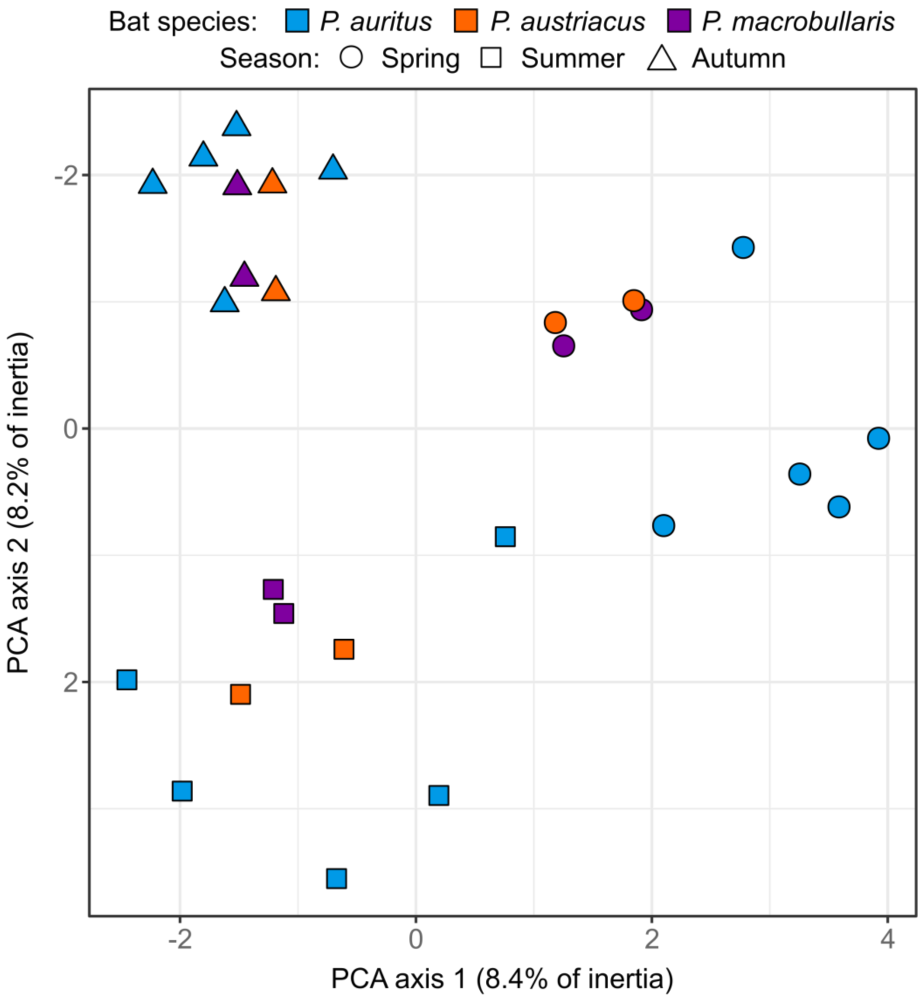

The first PCA axis positively correlates with the spring season; the second axis separates samples collected in summer (negative values) from those collected in autumn (positive values). The following table provides the PCA loadings for prey species correlating the most with the two first axes, either positively or negatively.

Prey species	PCA1 loading
Helicoverpa armigera	-0.1983
Mesapamea secalis/secalella/didyma	-0.1854
Nomophila noctuella	-0.1829
Hoplodrina ambigua	-0.1769
Charanyca trigrammica	0.1497
Tipula vernalis	0.1712
Nephrotoma appendiculata	0.1806
Rhizotrogus aestivus	0.1959

Prey species	PCA2 loading
Cosmia trapezina	-0.2128
Hoplodrina blanda	-0.1807
Apamea monoglypha	-0.1642
Macdunnoughia confusa	-0.1627
Conistra vaccinii	0.1057
Aporophyla lueneburgensis	0.1137
Triodia sylvina	0.1259
Xestia xanthographa	0.1811

Appendix 8. RLQ analysis of prey habitat traits and their occurrence in the diet of the different *Plecotus* species

Summary of the the joint structure among the three tables: R, containing characteristics of samples (here the bat species to which a sample was associated); Q, containing species traits (here ecological data about over 600 prey species); and the linking table L, containing prey species distribution across the samples (here the prey composition for each bat species considered as a sample).

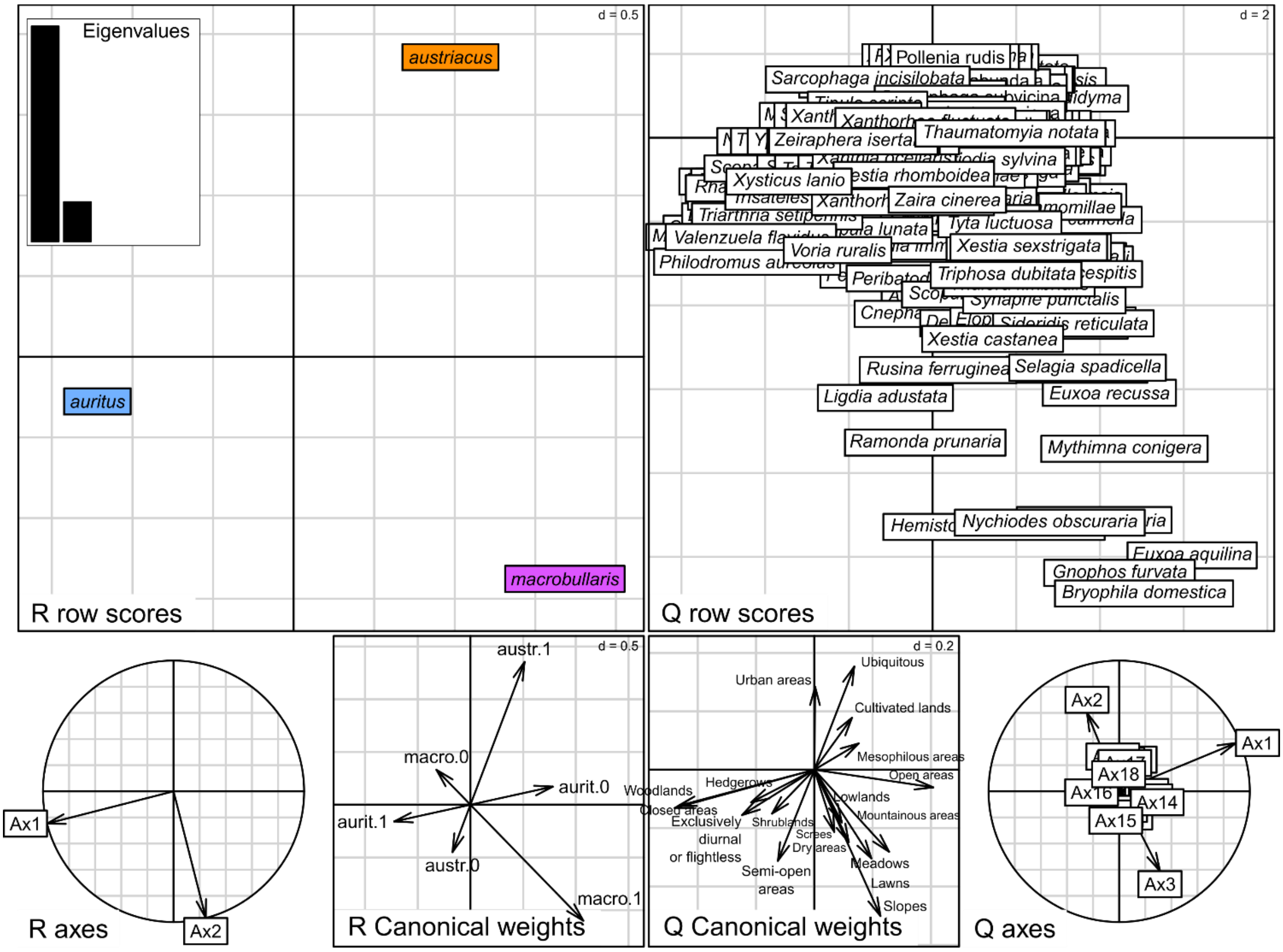

The first axis negatively correlates with prey species consumed by *P. auritus* and ecological traits related to closed habitats, diurnality and flightlessness; the second axis mostly correlates with prey of *P. austriacus* and traits such as ubiquiteness and urban areas (positive values) vs. prey of *P. macrobullaris* and traits relative to mountainous areas (negative values).
